# Disorder-Mediated Ionic Conductivity in Irreducible Solid Electrolytes

**DOI:** 10.1021/jacs.5c02784

**Published:** 2025-05-26

**Authors:** Victor Landgraf, Mengfu Tu, Wenxuan Zhao, Anastasia K. Lavrinenko, Zhu Cheng, Jef Canals, Joris de Leeuw, Swapna Ganapathy, Alexandros Vasileiadis, Marnix Wagemaker, Theodosios Famprikis

**Affiliations:** Faculty of Applied Sciences, 2860 Delft University of Technology, 2629JB Delft, The Netherlands

## Abstract

Solid-state batteries currently receive extensive attention due to their potential to outperform lithium-ion batteries in terms of energy density when featuring next-generation anodes such as lithium metal or silicon. However, most highly conducting solid electrolytes decompose at the low operating voltages of next-generation anodes leading to irreversible lithium loss and increased cell resistance. Such performance losses may be prevented by designing electrolytes which are thermodynamically stable at low operating voltages (anolytes). Here, we report on the discovery of a new family of *irreducible* (i.e., *fully reduced*) electrolytes by mechanochemically dissolving lithium nitride into the Li_2_S antifluorite structure, yielding highly conducting crystalline Li_2+*x*_S_1–*x*_N_*x*_ phases reaching >0.2 mS cm^–1^ at ambient temperature. Combining impedance spectroscopy experiments and *ab initio* density functional theory calculations we clarify the mechanism by which the disordering of the sulfide and nitride ions in the anion sublattice boosts ionic conductivity in Li_2+*x*_S_1–*x*_N_*x*_ phases by a factor 10^5^ compared to the Li_2_S host structure. This advance is achieved through a novel theoretical framework, leveraging percolation analysis with local-environment-specific activation energies and is widely applicable to disordered ion conductors. The same methodology allows us to rationalize how increasing nitrogen content in Li_2+*x*_S_1–*x*_N_*x*_ antifluorite-like samples leads to both increased ionic conductivity and lower conductivity-activation energy. These findings pave the way to understanding disordered solid electrolytes and eliminating decomposition-induced performance losses on the anode side in solid-state batteries.

## Graphical abstract

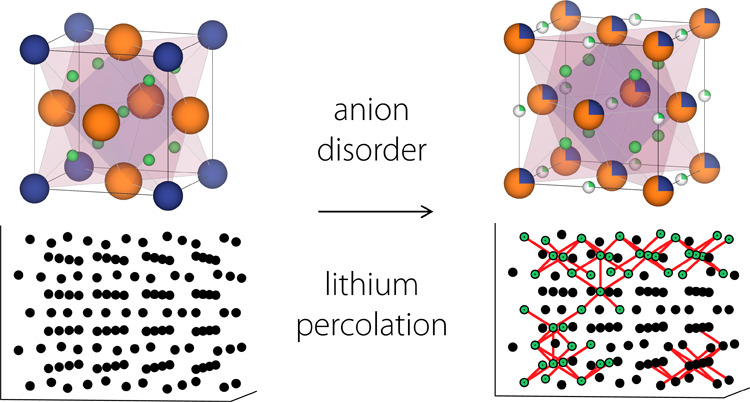

## Introduction

Solid-state batteries (SSBs) are recently receiving considerable attention in the scientific community because of their potential to outperform conventional lithium-ion batteries.^1,2^ The potential advantages of SSBs originate from their solid nature and single-ion conductivity, improved safety and the possibility of efficient cell stacking (*bipolar stacking*).^1^ SSBs will likely only overcome conventional lithium-ion batteries in terms of energy density, if they feature low-potential, high-energy-density anodes such as silicon (ref 3) or lithium-metal anodes.^4,5^ Still, most highly conducting solid electrolytes decompose at the low potentials of silicon and lithium metal anodes.^6–8^ The decomposition into a solid electrolyte interphase (SEI) entails irreversible lithum loss—particularly an issue for industrially attractive *zero-Li-excess* battery cells.^9^ Irreversible lithium loss from SEI formation may be mediated in three ways: (i) by limiting the contact area between the solid electrolyte and anode,^10,11^ (ii) by adding sacrificial lithium agents such as for instance Li_3_N to the cathode^12,13^ or (iii) by designing solid electrolytes which are thermodynamically stable at the operating potentials of low-potential anodes.

*Irreducible* or *fully reduced* phases are thermodynamically stable against lithium metal and are thus inherently irreducible against low-potential anodes. *Irreducible* refers to all elements (except for Li) in the material being in their lowest possible formal oxidation state (i.e., *fully reduced)* and thus not further reducible. Examples of irreducible phases are lithium binaries (e.g., Li_2_S, LiCl, LiBr, Li_2_O, Li_3_N), lithium-rich antiperovskites (e.g., Li_3_OCl, Li_3_OBr; see refs 14,15 ), Li_5_NCl_2_ (refs 16,17 ) and the recently discovered Li_2+*x*_S_1–*x*_P_*x*_ (0 < *x* < 0.75) solid solution.^18^ An issue with irreducible phases thus far has been that their ambient-temperature conductivities do not typically reach values above 0.05 mS cm^–1^—Li_3_N and Li_2.75_S_0.25_P_0.75_ are exceptions with RT conductivities of 0.5 mS cm^–1^ (ref 19) and ∼0.25 mS cm^–1^ (ref 18), respectively. The latter Li_2+*x*_S_1–*x*_P_*x*_ (0 < *x* < 0.75) solid solution^18^ is characterized by a disordered anion lattice, yet the effect of the structural disorder on ion conductivity has not yet been clarified.

Structural disorder on the atomic scale—often occupational (i.e., characterized by multiple partially occupied cation positions) and/or compositional (i.e., characterized by mixed occupation of framework sites by multiple different atoms)—is in fact a common feature of most highly conductive solid electrolytes. Both of these types of disorder feature, for example, in the well-studied argyrodite family of ion conductors with the archetypical formula Li_6_PS_5_X (X: Cl, Br). Nevertheless, the correlation between disorder and ionic conductivity remains a qualitative one. Zeng et al. argued in a recent study that the (often) enhanced conductivity in compositionally disordered solid electrolytes originates from the increased energy-overlap between individual carrier-ion (Li, Na, ···) sites, enabling low-energy percolation paths through solid-electrolyte crystallites—without explicitly considering the energetics of ion hops but instead based on the assumption that sites similar in energy are connected by low activation barriers.^20^

In the present study, we report the discovery of a new family of irreducible solid electrolytes with the general formula Li_2+*x*_S_1–*x*_N_*x*_ (0 < *x* < 0.55) reaching high conductivities above 0.2 mS cm^–1^. These are metastable phases, accessible by mechanochemistry and feature a disordered face-centered-cubic arrangement of nitride and sulfide anions. We further develop a widely applicable methodology to investigate the effect of disorder on conductivity that explains the often observed conductivity increase with increased structural disorder. The herein developed methodology comprises the analysis of ion-hop activation energies from molecular dynamics (MD) as a function of local environments and their connectivity via percolation analysis.

We leverage this MD-percolation methodology to rationalize the conductivity boost in the disordered Li_2+*x*_S_1–*x*_N_*x*_ phases. We find that the disordered N/S anion arrangement in Li_2+*x*_S_1–*x*_N_*x*_ electrolytes is causally related to their vastly increased ionic conductivity compared to the structurally and chemically related anion-ordered Li_2_S and Li_9_S_3_N (refs 21,22 ), by allowing low-activation-energy ion jumps through locally nitrogen-rich bottlenecks. We show how the MD-percolation methodology may be applied to other disordered solid electrolytes which we demonstrate on the example of the Li_6_PS_5_Br argyrodite.

## Results and Discussion

### Synthesis of Disordered-Li_9_S_3_N

Previous investigations on the Li_2_S–Li_3_N tieline identified the anion-ordered Li_9_S_3_N phase accessible by conventional solid-state synthesis.^21,22^ After reproducing said synthesis (SI Figure S1 and Table S1), we attempted to synthesize Li_9_S_3_N mechanochemically, through milling stoichiometric amounts of the precursors (Li_2_S and Li_3_N). The X-ray and neutron diffraction patterns of the resulting product did not show any leftover precursors (Figures 1 and S3) and we verified through diffraction that no significant amorphous fraction or amorphous impurities are present in samples synthesized with this approach (see Supporting Note 1).

**Figure 1:**
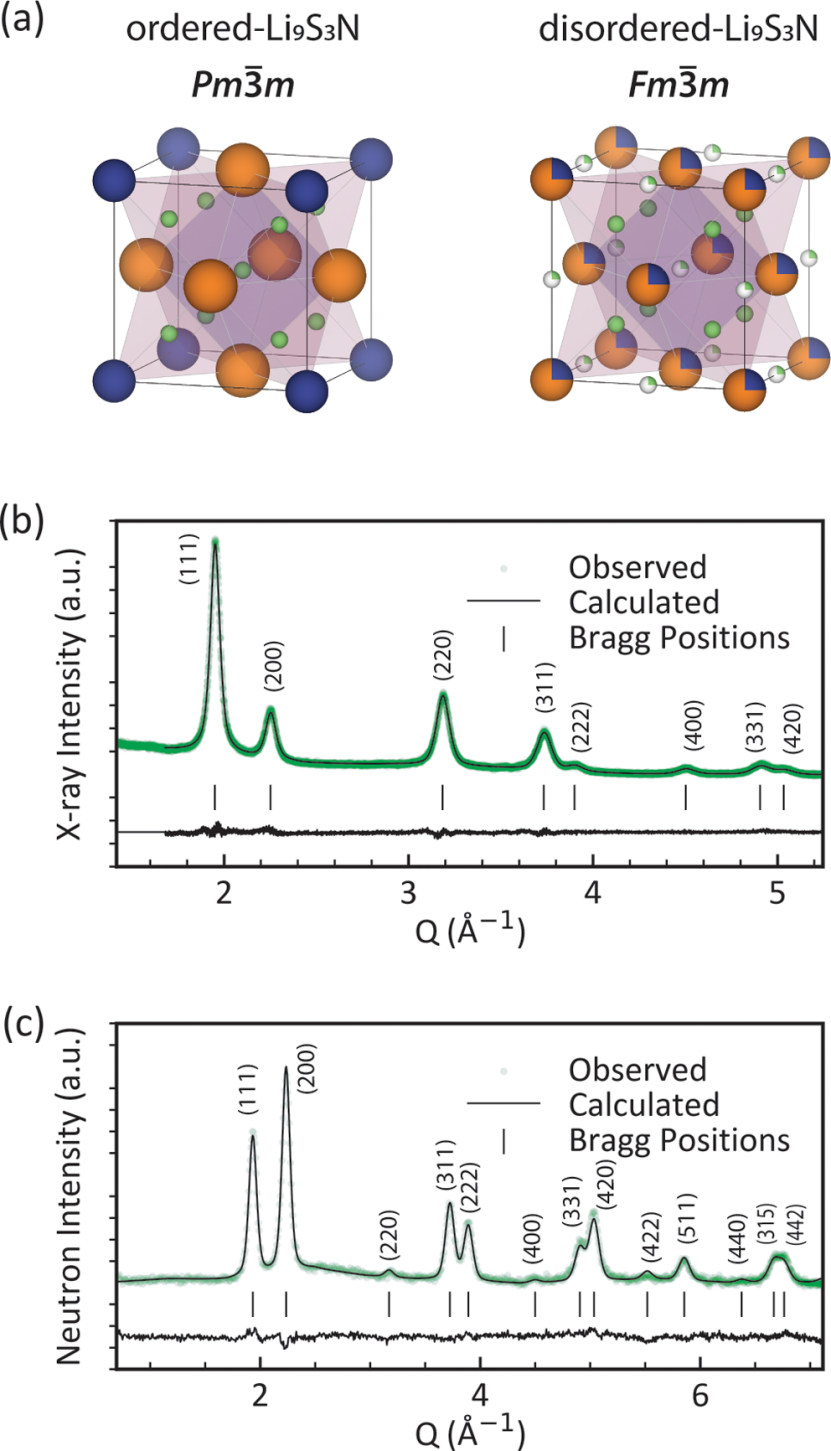
Neutron and X-ray powder diffraction on mechanochemically synthesized anion-disordered Li_9_S_3_N. (a) Unit cell of anion-ordered and anion-disordered Li_9_S_3_N. (b) X-ray- and (c) neutron diffraction patterns of mechanochemically synthesized anion-disordered Li_9_S_3_N along with Rietveld refinements in the *F* *m*3̅*m* space group (see also Figure S3 and Table S3).

Interestingly, poor Rietveld refinements were obtained when attempting to fit the same *Pm*3̅*m*-Li_9_S_3_N structure solution proposed by Marx et al.^21^ to the neutron and X-ray diffraction patterns of the mechanochemically prepared product (SI Figure S2). The *Pm*3̅*m*-Li_9_S_3_N structure solution proposed by Marx et al.^21^ (inset Figure 1a, and SI Table S1) is closely related to the antifluorite (*Fm*3̅*m*) structure of Li_2_S but the face-centered symmetry is broken by the ordered arrangement of sulfur and nitrogen and an additional Li yielding a unit cell with a lower-symmetry *Pm*3̅*m* space group and more non-zero-intensity diffraction peaks.

The absence of certain diffraction peaks in the measured diffraction pattern of mechanochemically synthesized Li_9_S_3_N suggests that the crystal structure of mechanochemically prepared Li_9_S_3_N has a higher symmetry than the *Pm*3̅*m* structure that Marx et al.^21^ proposed for ampule-synthesized Li_9_S_3_N. The increased symmetry originates from a mechanochemically induced disordering of the S and N atoms. Based on neutron and X-ray Rietveld refinements (SI Figure S3) we propose the following structure for mechanochemically prepared Li_9_S_3_N (SI Table S3): Cubic *Fm*3̅*m* with S and N sharing occupation of the Wyckoff 4a (0,0,0) position in a 3:1 proportion as imposed by the Li_9_S_3_N stoichiometry. The tetrahedral interstitial on the Wyckoff 8c (0.25, 0.25, 0.25) position is fully occupied by Li and the octahedral interstitial on the Wyckoff 4b (0.5, 0.5, 0.5) position is partially occupied (25%) by Li (Figure 1a). In that sense the structure can be considered as a lithium-rich antifluorite, i.e., intermediate between the antifluorite (only tetrahedral sites fully occupied; e.g., Na_2_O, Li_2_S) and Li_3_Bi (both tetrahedral and octahedral sites fully occupied) archetypical structures based on interstitial-filling of ccp-like arrangement of anions.

The proposed lithium-rich anion-disordered antifluorite (*Fm*3̅*m*) structure for disordered-Li_9_S_3_N is directly analogous to that of the Li_2+*x*_S_1–*x*_P*_x_* (0 < *x* < 0.75) phases^18^ and closely related to the lithium-deficient anion-disordered antifluorite lithium-nitride-halide Li_1+2*x*_Cl_1–*x*_N_*x*_ (refs 16,23 ) as well as the cation-disordered lithium-rich antifluorites ω*-*Li_9_TrP_4_ (Tr = Al, Ga, In)^24^ and Li_14_SiP_6_ (ref 25).

We thus discovered a new material which can be interpreted as a disordered polymorph of the previously known *Pm*3̅*m* phase.^21^ Based on our structure solution we will from now on refer to the mechanochemically synthesized, anion-disordered (***Fm*** 3̅ ***m***) Li_9_S_3_N as *disordered*-Li_9_S_3_N and to solid-state-synthesized, anion-ordered (*Pm*3̅*m*) Li_9_S_3_N as *ordered*-Li_9_S_3_N.

The crystallographic relationship between ordered and disordered Li_9_S_3_N can be formally expressed as a group-subgroup relationship (Bärnighausen tree) as shown in SI Figure S4. This relationship implies the possibility of an order–disorder phase transition at elevated temperature, meaning it might also be possible to stabilize disordered-Li_9_S_3_N at ambient temperature through quenching, as an alternative to the mechanochemical route reported here, as has been reported e.g., for the structurally related ω-Li_9_AlP_4_ (ref 24) and Li_14_SiP_6_ (ref 25).

We note that the structure solution we propose for disordered-Li_9_S_3_N (Figure 1a and SI Table S3) features large thermal parameters on the Li-sites (*U*_iso_ > 0.07 Å^2^). An in-depth structure analysis supported by molecular dynamics simulations (Supporting Note 2) indicates that these large *U*_iso_ values likely originate from displacive relaxations of lithium ions off their ideal positions correlated to the specific local N/S coordination. We also propose an alternative structure solution, in which the octahedral lithium positions are further resolved via site-splitting in combination with lower *U*_iso_ values. This feature of large *U*_iso_ on the octahedral side resolvable through site-splitting was also observed in the isostructural Li_2+*x*_S_1–*x*_P_*x*_ compounds.^18^ Still, the simple structure solution presented in Figure 1a and SI Table S3 captures all the essential features to describe the disordered-Li_9_S_3_N phase for all the following discussions.

### Effect of S/N Disordering on the Conductivity in Disordered-Li_9_S_3_N

To compare the ionic conductivities of ordered- and the newly discovered disordered-Li_9_S_3_N we performed variable-temperature impedance spectroscopy experiments on pelletized powder samples (Figure 2). Interestingly, we found an activation energy reduced by 80 meV and a significant ambient-temperature conductivity increase by a factor 30 for disordered-Li_9_S_3_N (0.064 mS cm^–1^), compared to ordered-Li_9_S_3_N (0.0018 mS cm^–1^). Next, we occupy ourselves with the underlying mechanism that enabled the 30-fold conductivity increase and the reduced activation energy in disordered-Li_9_S_3_N compared to ordered-Li_9_S_3_N.

**Figure 2:**
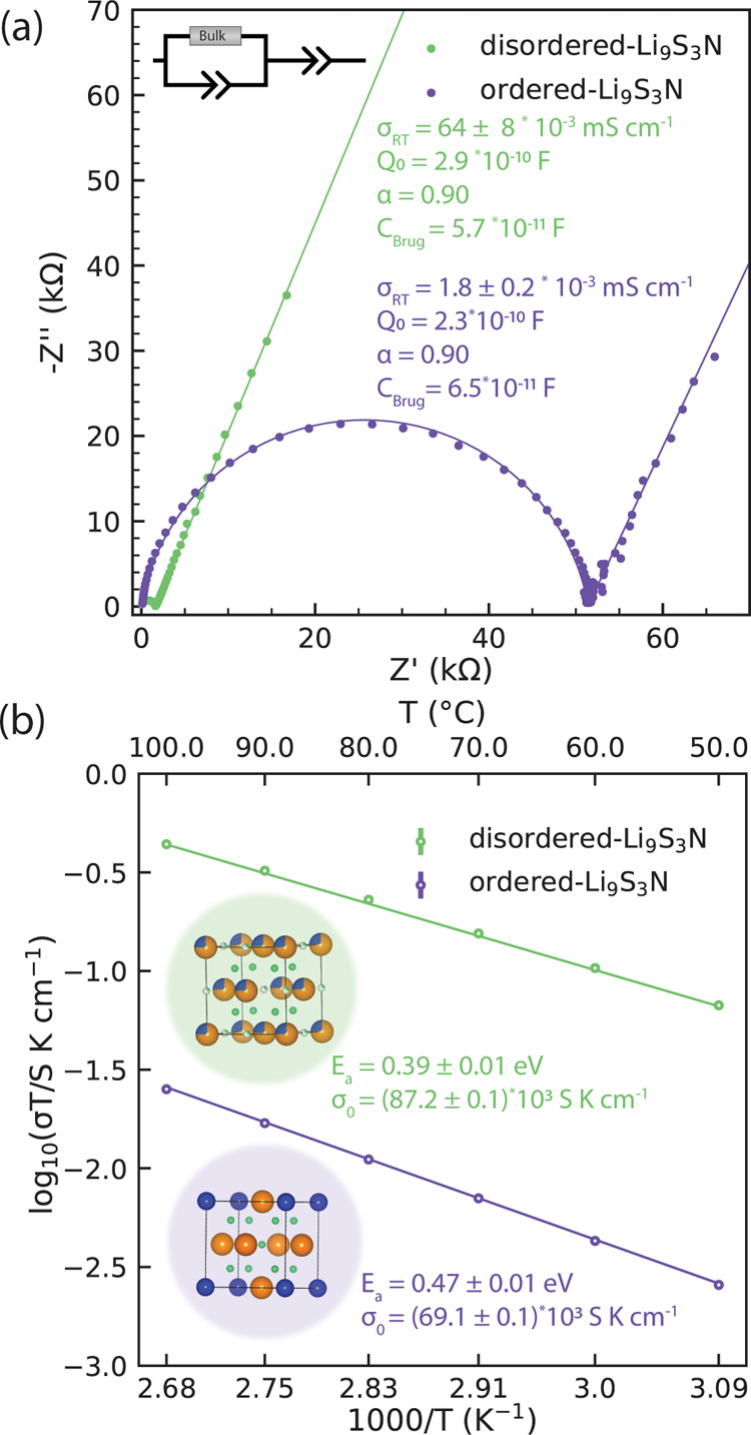
Impedance spectroscopy results for ordered and disordered Li_9_S_3_N. (a) Room-temperature impedance spectra fitted with the equivalent circuit inset. (b) Arrhenius conductivity plots and fits. All data points obtained from at least two measurements. Error bars often smaller than marker symbol. Inset: structural models with Li, S, N in green, orange, blue, respectively.

Ordered- and disordered-Li_9_S_3_N feature the same face-centered-cubic anion framework and the same lithium (and thus, vacancy) content and so the change in conductivity cannot be ascribed to the overall concentration of charge carriers. Thus, we hypothesize that the vastly different ionic conductivity observed in Figure 2 should originate in changes to the relative mobility of Li^+^ ions as a function of the different local structure. To probe this hypothesis we initiated a series of *ab initio* molecular dynamics (AIMD) simulations with ordered-Li_9_S_3_N and disordered-Li_9_S_3_N supercells. In disordered supercells the Wyckoff 4a position was randomly decorated with N and S, respecting the 1:3 ratio imposed by the Li_9_S_3_N stoichiometry. The Wyckoff 4b position was also randomly decorated with Li atoms and vacancies respecting the overall stoichiometry. As done in previous studies we dissected our AIMD simulations into individual jump events.^16,26–29^ In-depth analysis of the AIMD simulations shows that well-defined sites exist in (dis)­ordered Li_9_S_3_N and that jumps between these sites occur mostly independently (i.e., *no* evidence of correlated ion jumps, correlated “cascades” of jumps or correlated “strings” of jumps is found which have been reported in other high-conducting solid electrolytes such as Li_6_PS_5_Cl (ref 30) and Li_10_GeP_2_S_12_ (ref 31), see Supporting Note 3).

From the frequency of jumps between two sites (ν_A→B_) we calculate so-called jump-activation energies (jump-*E*_a_) by using eq 1

1 $$jump‐E_{a,A\to B}=-k_{b}T\cdot \ln\left(\frac{\nu_{A\to B}}{\nu_{0}}\right)$$

where jump-*E*_a,A→B_ the jump-activation energy of a jump event from site A to site B, *k*_b_ the Boltzmann constant, *T* the temperature in K, ν_A→B_ the observed frequency of jumps between sites A and B and ν_0_ the attempt frequency, which we assume to be 10^13^ Hz. The latter is a widely accepted approximation for ceramic ion conductors^32–35^ and we additionally verified that this approximation applies for the Li_9_S_3_N system (see Supporting Note 4).

The jump-*E*_aA→B_ is a rescaled jump frequency that we interpret as a proxy for the time-averaged local ion hop activation energies and thus the ease of the ion jump from site A to site B. We note that, while both quantify the ease of migration, energy barriers obtained from nudged-elastic-band calculations and the jump-*E*_a_ values from AIMD used here are conceptually different and not necessarily equivalent as explained in Supporting Note 5.

Adopting the above-described approach, we could assign an individual jump-*E*_a_ values to each different jump type based on local coordination and bottleneck composition. Irrespective of local anion ordering, three general families of jumps are observed through the face-centered anion arrangement in Li_9_S_3_N. (i) tetrahedron­(8*c*)-to-octahedron­(*4b*) (tet-oct), (ii) octahedron­(*4b*)-to-tetrahedron­(*8c*) (oct-tet) and (iii) tetrahedron­(*8c*)-to-tetrahedron­(*8c*) (tet-tet) jumps. Tetrahedral sites are connected to adjacent octahedral sites via triangular bottlenecks composed of three anions, whereas two tetrahedral sites are connected through linear bottlenecks composed of two anions. This observation of ion conduction proceeding through hops between tetrahedral and octahedral interstitials is consistent with previous understanding of Li-ion conduction in antifluorite-like materials^23,25,36^

The possible lithium coordination environments in lattice sites as well as bottlenecks are shown schematically in Figure 3 for ordered- and disordered-Li_9_S_3_N. We categorize a jump event by its start-site, its end-site and the bottleneck connecting the two sites and use a *start-end­(bottleneck)* notation. For example, a S_3_N–S_6_(SSS) jump is a tet-oct jump which starts at a tetrahedral-Li site where the corners of the tetrahedron are occupied by three sulfide and one nitride ion for which we use the notation S_3_N_1_. From there on the jump path proceeds through a triangular bottleneck consisting of three sulfide ions for which we use the notation SSS. The end-point of this jump is an octahedral Li site where the corners of the octahedron are all occupied by sulfide ions for which we use the notation S_6_.

**Figure 3:**
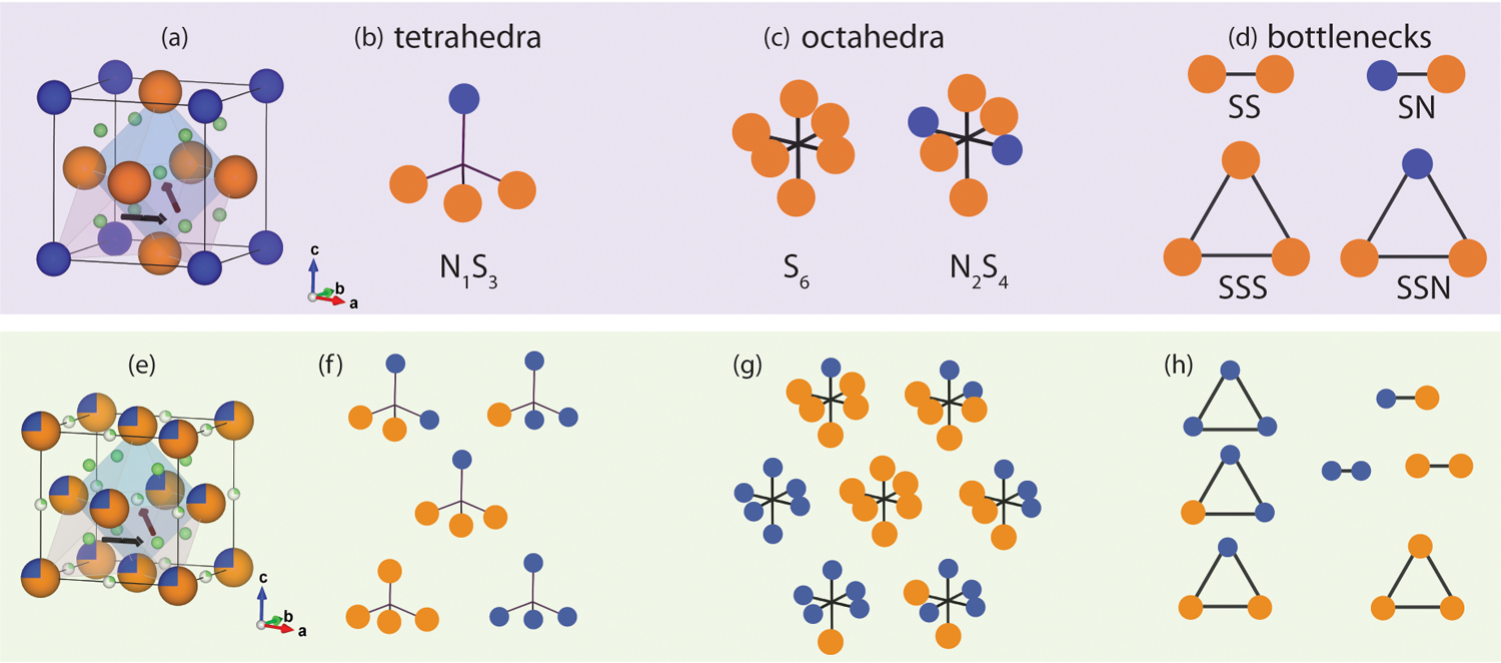
Local environments in ordered- and disordered-Li_9_S_3_N. (a) unit cell of ordered-Li_9_S_3_N (*Pm*3̅*m*). Example, tetrahedral and octahedral lithium positions in pink and blue, respectively. Black arrows indicate tet-tet and tet-oct jump paths. (b) single tetrahedral site, S_3_N_1_ present in ordered-Li_9_S_3_N. (c) Two octahedral sites present in ordered-Li_9_S_3_N: S_6_ and S_4_N_2_. (d) Two linear bottlenecks (SS, NS) and two triangular bottlenecks (SSS, SSN) present in ordered-Li_9_S_3_N. (e) unit cell of disordered-Li_9_S_3_N (*Fm*3̅*m*). (f) All five possible tetrahedral compositions in disordered-Li_9_S_3_N. (g) all seven possible octahedral coordinations in disordered-Li_9_S_3_N. (h) all three linear and four triangular bottlenecks possible in disordered-Li_9_S_3_N. Li, S, N in green, orange, blue, respectively.

Figure 3a,3e schematically illustrate the tet-tet and oct-tet jump paths through the linear and triangular bottlenecks for the crystal structure of ordered-Li_9_S_3_N and disordered-Li_9_S_3_N, respectively. Figure 3 shows that the disorderly arrangement of S/N in disordered-Li_9_S_3_N enables a large manifold of different jump types (91 jump-types, SI Table S5), as a result of the possible permutations between starting site, ending site and bottleneck compositions. For instance, taking the example of triangular bottlenecks, in disordered-Li_9_S_3_N, SSS, NSS, NNS and NNN bottlenecks may exist whereas in ordered-Li_9_S_3_N only SSS and NSS bottlenecks exist (Figure 3d,h).

To enable high conductivity, SEs should feature low-energy percolating paths consisting of a series of connected Li jumps with low jump-activation energies. Figure 4a shows the jump-*E*_a_ values of individual jump types segregated by local environment and determined by eq 1 from the MD trajectories. Because of its ordered S/N arrangement, ordered-Li_9_S_3_N merely features 6 discrete jumps which are shown as discrete points in Figure 4a. The uncertainty on individual jump-*E*_a_ values is in the range of 10–30 meV and comprises uncertainty associated with convergence as further explored in Supporting Note 6. The tet-oct jumps being generally higher in energy than the oct-tet jumps is coherent with the fact that the octahedral sites are generally higher in energy compared to the tetrahedral ones (SI Figure S5), which in turn is coherent in the crystallographic model of full tetrahedral- and only partial octahedral Li occupation.

**Figure 4:**
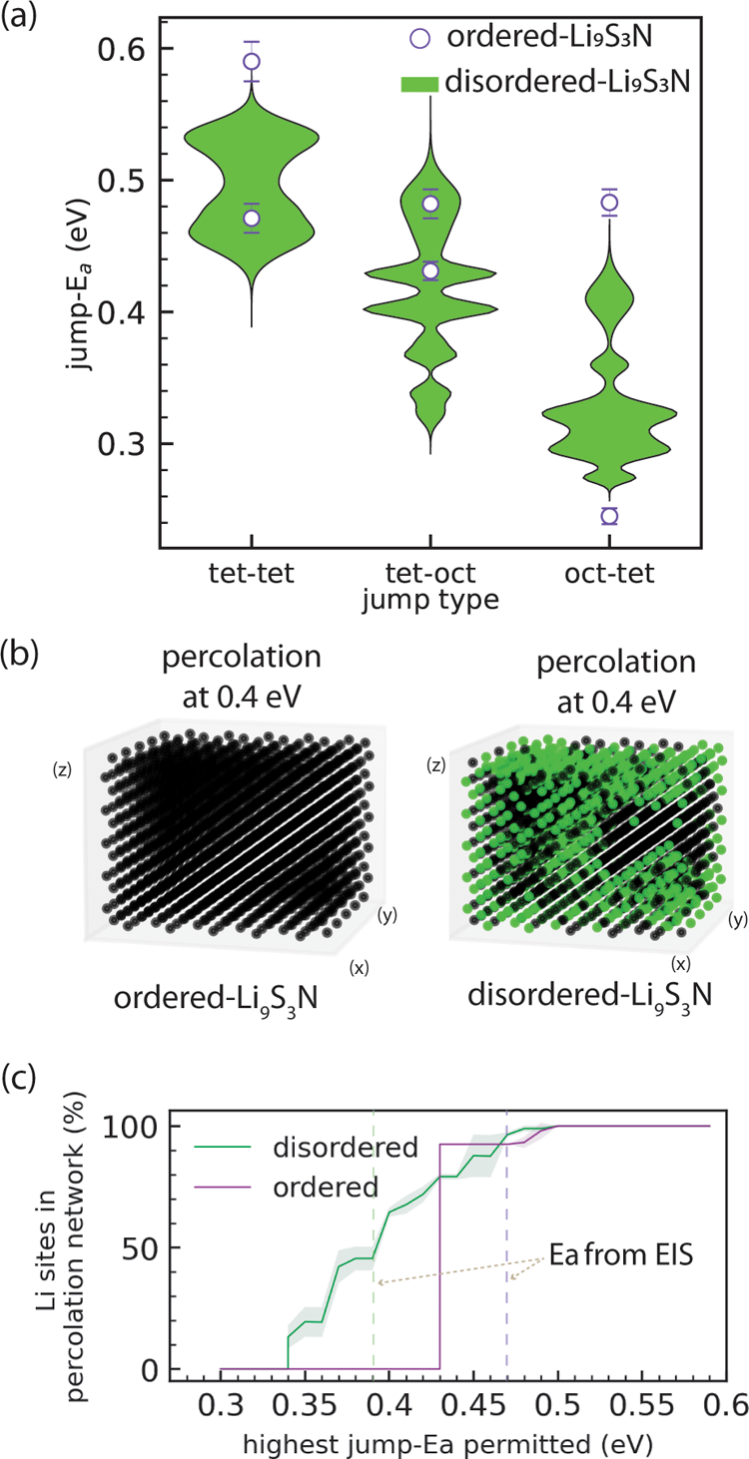
Effect of different jump-types existing in ordered and disordered Li_9_S_3_N. (a) Comparison of the observable jump-activation energies in ordered and disordered-Li_9_S_3_N. Purple markers indicate jump-*E*_a_ of six distinct jump types possible in ordered-Li_9_S_3_N, labeled with the *start-end­(bottleneck)* notation explained in the main text. In disordered-Li_9_S_3_N 91 different jumps are observable, listed in SI Table S5 and shown here as density plots (*violins*). The horizontal scale of the violins represent the relative occurrence of jump types at that energy. (b) Lattice of 1500 lithium sites for ordered and disordered Li_9_S_3_N (5 × 5 × 5) supercells. Sites highlighted in green are part of percolating networks with an energy threshold ≤0.4 eV. (c) Percolation-energy diagram showing the fraction of Li sites that are connected to percolating networks (averaged over 50 disordered 5 × 5 × 5 supercells) for ordered- and disordered-Li_9_S_3_N. Shading is the standard deviation over 50 supercells. The vertical lines indicate the experimental activation energy *E*_a,EIS_ obtained experimentally from EIS for ordered- and disordered-Li_9_S_3_N (see Figure 2).

In both phases, ordered- and disordered-Li_9_S_3_N, long-range bulk diffusion occurs along *tet-oct-tet* or *tet-tet* diffusion paths. To illustrate the effect of the determined jump-*E*_a_ values on Li diffusion we take the (arbitrary) example of a jump-*E*_a_ threshold of 0.4 eV: Ordered-Li_9_S_3_N does not feature tet-oct or tet-tet jumps with a jump-*E*_a_ < 0.4 eV. Thus, *tet-oct-tet* or *tet-tet*diffusion paths—necessary for percolation—where each individual jump has a jump-*E*_a_ < 0.4 eV cannot exist and thus no percolation is possible with an overall activation-energy threshold <0.4 eV.

In contrast to ordered-Li_9_S_3_N, the disordering of S/N in disordered-Li_9_S_3_N enables a large manifold of 91 different jump types, of which the distribution of jump-activation energies are shown in Figure 4 as density (*violin*) plots. In contrast to the ordered case, the disordering of anions creates tet-oct and tet-tet jumps with jump-*E*_a_’s < 0.4 eV. Thus, *tet*-*oct-tet* and *tet*-*tet* diffusion paths where each individual jump has a jump-*E*_a_ < 0.4 eV are conceivable—and thus percolation with an overall activation-energy threshold <0.4 eV is possible. From a comparison of the same jump types in ordered and disordered Li_9_S_3_N supercells it becomes apparent that the long-range anion ordering in ordered-Li_9_S_3_N has a Li-diffusion hampering effect in addition to the local N/S occupation of polyhedra and bottlenecks as further explored in Supporting Note 7.

In summary, the above analysis presented in Figures 3 and 4 suggests that the improved conductivity of disordered-Li_9_S_3_N originates from the disorder-induced numerous possibilities of octahedral and tetrahedral local coordination environments which introduce new jump-types with low jump-activation energies which enable lower-energy percolating paths that cannot exist in the ordered case.

To consolidate the hypothesis that percolating lower-energy diffusion paths exist in disordered-Li_9_S_3_N we designed a percolation model. In this percolation model, 5 × 5 × 5 supercells are considered, containing 125 formula units of Li_9_S_3_N, and 1500 lithium sites (Figure 4b). Each Li site is related to its neighbors based on the jump-activation energies determined previously. A connection is made between two Li sites if the jump-*E*_a_ for both the forward and the backward jump are below a defined jump-*E*_a_ cutoff value. If—for a defined jump-*E*_a_ cutoff value—a connected path can be found spanning the supercell, then the path is *percolating*, provided that the end-point of percolation is itself a starting-point of a percolating path (as illustrated in SI Figure S6).

The results of our percolation analysis are demonstrated in Figure 4b,4c. Figure 4b shows that for ordered-Li_9_S_3_N no percolating path exists when the jump-*E*_a_ cutoff is set to 0.4 eV. In contrast, for a disordered-Li_9_S_3_N supercell with the same jump-*E*_a_ cutoff of 0.4 eV a clear percolating network is obtained. Our model also determines the number of Li sites that are connected to the percolating network. As the example in Figure 4b demonstrates, even if a percolating network exists, a fraction of Li sites may still be disconnected which may lead to a fraction of Li sites which do not (or significantly more slowly) participate in Li-ion diffusion than Li sites in the percolating network. Li sites in disordered-Li_9_S_3_N may thus be segregated in *active* and *inactive* sites with regard to long-range lithium diffusion[Fn fn1]. In other words, the majority of jump events involves only a subset of sites that predominantly contribute to the diffusivity (*active*) while the rest remain invariantly vacant or occupied throughout much of the simulation (*inactive*); as may be directly observed from the frequency of occupation change in our AIMD simulations (SI Figure S7).

Figure 4c is a percolation-energy diagram and shows the fraction of Li sites in percolating networks for ordered-Li_9_S_3_N and disordered-Li_9_S_3_N (average of 50, 5 × 5 × 5 supercells) as a function the highest jump-*E*_a_ value allowed in the percolation network. The onset of percolation—that is the lowest activation energy for which a percolation network can exist—is markedly lower in the disordered case (0.34 eV) compared to the ordered case (0.43 eV). This is a direct reflection of the lower energy tet-tet and tet-oct jumps available in the disordered case shown in Figure 4a but additionally highlights that their connectivity is sufficiently likely to enable percolation paths at lower energy thresholds. The lower energy of percolation onset of disordered-Li_9_S_3_N suggests that long-range diffusion can be sustained more easily in disordered-Li_9_S_3_N than in ordered-Li_9_S_3_N. The presence of diffusion at lower energy thresholds is indeed experimentally reflected in the lower activation energy of disordered- Li_9_S_3_N (0.39 eV) compared to ordered- Li_9_S_3_N (0.47 eV, Figure 2b). In both cases the conductivity-activation energy is 0.04–0.05 eV higher than the simulated percolation-onset energy.

The fact that the experimental *conductivity-activation* energy values are slightly higher than the simulated *percolation-onset* energy values is consistent with the expectation that percolation networks at higher energies than the percolation onset also contribute to the overall diffusion (see Supporting Note 8).

We conclude at this stage that the increased conductivity of disordered-Li_9_S_3_N is a consequence of the disordered anionic sublattice which enables numerous octahedral and tetrahedral lithium coordination by combinations of sulfide and nitride ions. Instead of only having S_6_, S_4_N_2_ and N_1_S_3_ polyhedra like in ordered-Li_9_S_3_N, disordered-Li_9_S_3_N features a wide manifold of polyhedra (N_2_S_2_, S_3_N_3_, S_2_N_4_, S_3_N_1_ etc.). The diverse configurations of the polyhedra in disordered-Li_9_S_3_N create new sites and bottlenecks which are simply not present in ordered-Li_9_S_3_N. Some among these new sites and low-energy bottlenecks enable lower-energy percolation and thus the increased conductivity in disordered-Li_9_S_3_N. The presented mechanism for disorder-induced conductivity enhancement and the analysis approach developed here for Li_9_S_3_N are widely applicable to other solid electrolytes as we demonstrate using the example of the entirely different Li_6_PS_5_Br argyrodite system in Supporting Note 9.

The presented analysis of local jump environments and percolation also enables to optimize ion diffusion by identifying diffusion-promoting and diffusion-hampering local environments. Subsequently, ion diffusion may be optimized by tuning the phase composition to increase the occurrence of diffusion-promoting environments as presented in the following sections.

### Understanding Diffusion Bottlenecks in Disordered-Li_9_S_3_N

Next we explore the relationship between bottleneck composition and local jump-activation energy in disordered-Li_9_S_3_N. It is expected that the composition of the bottlenecks affects the bottleneck size through the different sizes of the sulfide and nitride anions. The empty space available for Li^+^ in the bottlenecks for different oct-tet ion jumps may be estimated from geometrical considerations by determining the diameter of the circle inscribed in the triangle spanned by the surrounding anions (taking into account their anionic radii) as shown in Figure 5a (SI Table S7 summarizes the ionic radii used for the following considerations, SI Figure S8 shows how the bottleneck diameter is analogously obtained for linear bottlenecks between tet-tet jumps). The average bottleneck size and its standard deviation were calculated from 50 DFT-relaxed (2 × 2 × 2) disordered Li_9_S_3_N supercells (>9000 bottlenecks) to account for local distortions which may not be present in long-range averaged crystallographic unit cells.

**Figure 5:**
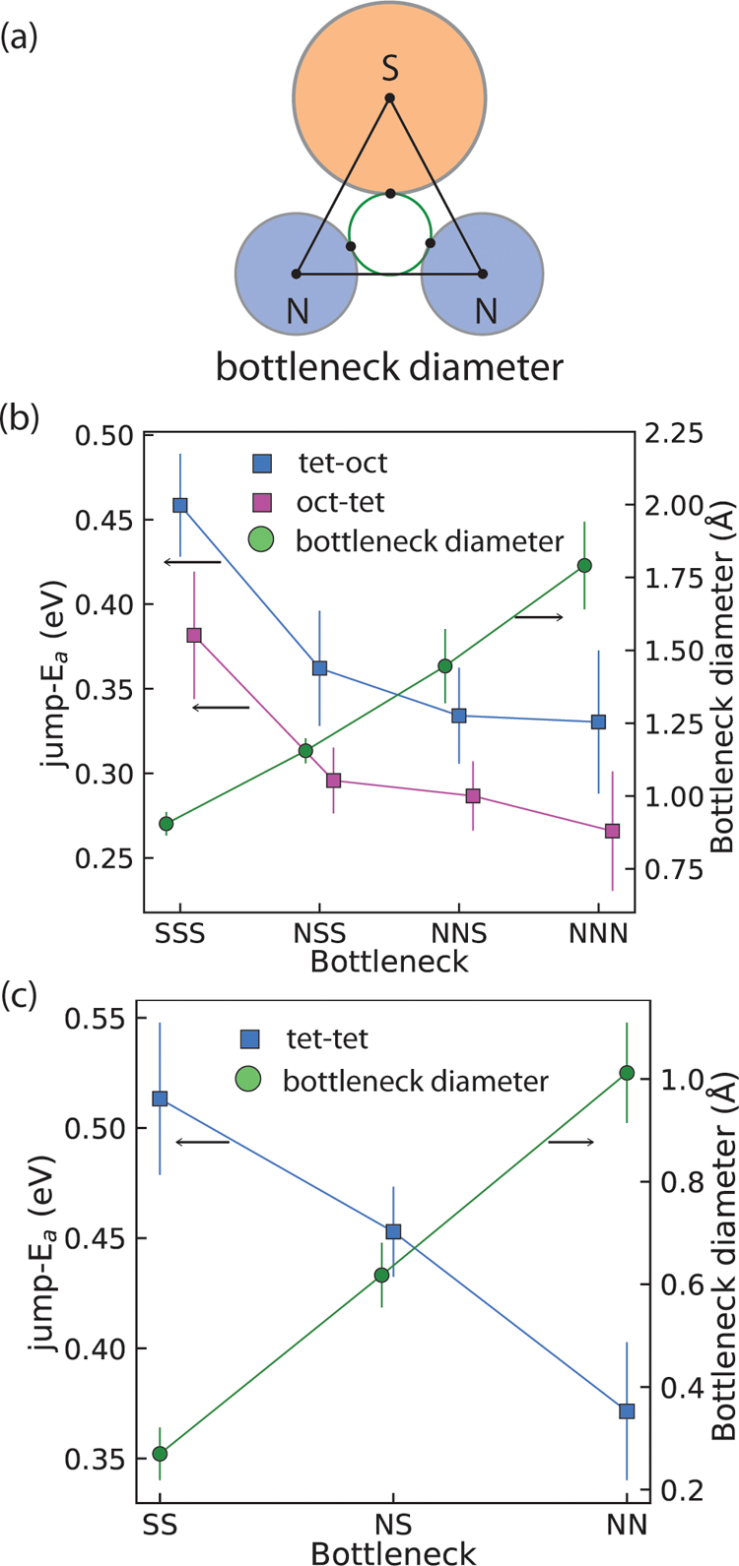
Relation between jump activation energies and bottleneck sizes in disordered-Li_9_S_3_N. (a) Illustration of bottleneck definition based on ionic radii of peripheral anions for tet-oct and oct-tet jumps. Analogous definition for tet-tet bottlenecks in SI Figure S8 (b) Average jump-*E*_a_ and bottleneck diameter for tet-oct and oct-tet jumps segregated by bottleneck composition. (c) Average jump-*E*_a_ and bottleneck diameter for tet-tet jumps segregated by bottleneck composition. Error bars represent standard deviations from the multiple occurrences of each bottleneck considered.

Figure 5b shows the bottleneck size and average jump-*E*_a_ for tet-oct and oct-tet jumps in disordered-Li_9_S_3_N as a function of the different possible jump bottlenecks. This analysis shows that the more nitrogen the bottleneck contains the larger the bottleneck which can be rationalized based on the small ionic radius of N^3–^ (1.46 Å, ref 37) compared to S^2–^ (1.84 Å, ref 37). Further, the more nitrogen a bottleneck contains the lower the jump-*E*_a_. The same trends hold for tet-tet jumps, shown in Figure 5c.

We thus observe a correlation between bottleneck size and jump-*E*_a_. In order to rationalize this observation, a useful benchmark is to compare the bottleneck sizes to the diameter of Li^+^ at about 1.18 Å. As the bottleneck size approaches the size of Li^+^, the jump-activation decreases, presumably because of the lessened energy penalty associated with an anion–cation approach smaller than the sum of their ionic radii. This is observed for the tet-tet cases (Figure 5c) and partly for the tet-oct cases (Figure 5b). For the latter, there is a stark effect in going from an SSS bottleneck (diameter: 0.90 Å) to NSS (1.15 Å) resulting in a decrease in jump-*E*_a_ of approximately 100 meV.

As the bottleneck size reaches and surpasses the size of Li^+^—that is, in the case of NSS, NNS and NNN triangular bottlenecks—the effect of bottleneck-diameter widening on decreasing jump-*E*_a_ is lessened and the corresponding jump-activation energies plateau. We quantify the amount of time lithium ions are in unfavorable proximity to the anions (defined as closer than the sum of their respective radii) in SI Figure S9, and show indeed that the lower jump-*E*_a_ correlates with less time spent too close to the anions.

We have thus established that the jump-*E*_a_ generally decreases the more nitrogen the bottleneck contains and we thus identified diffusion-promoting local environments. Based on this observation, we hypothesize that introducing more nitrogen into disordered-Li_9_S_3_N would increase the number of low-energy nitrogen-containing bottlenecks, thus increasing the number of lower-energy percolation paths, in turn leading to more facile ion conduction.

### Solid Solution between Li_3_N and Li_2_S: Lithium-Rich Disordered Antifluorite Phases Li_2+*x*_S_1–*x*_N_*x*_

To probe the hypothesis that nitrogen content controls ionic conductivity in the sulfide-nitride antifluorites, we synthetically explored compositions on the tie line between Li_2_S and Li_3_N. The two Li_9_S_3_N phases lie on the (1 – *x*)­Li_2_S-*x*Li_3_N tie line with *x* = 0.25. Our findings so far suggest that nitrogen-richer Li_2+*x*_S_1–*x*_N_*x*_ antifluorite phases—if existing—would likely have even higher conductivities than the disordered-Li_9_S_3_N (i.e., Li_2.25_S_0.75_N_0.25_) because of the higher occurrence of low-energy, nitrogen-rich bottlenecks.

Figure 6 shows the results of our synthetic exploration of the Li_2_S–Li_3_N tie line via mechanochemistry. For samples of overall stoichiometry (1 – *x*)­Li_2_S-*x*Li_3_N with 0 < *x* < 0.55, a single (*Fm*3̅*m*) antifluorite-like phase was observed in the diffractograms (shown in SI Figure S10), indicating that Li_3_N dissolves in the antifluorite structure of Li_2_S to form a Li_2+*x*_S_1–*x*_N_*x*_ solid solution of anion-disordered phases illustrated in Figure 6a. In the solid-solution range (0 < *x* < 0.55) the lattice parameters of Li_2+*x*_S_1–*x*_N_*x*_ phases decrease linearly with *x*, consistent with the smaller ionic radius of N^3–^ compared to S^2–^ and in accordance with Vegard’s law (Figure 6b).

**Figure 6:**
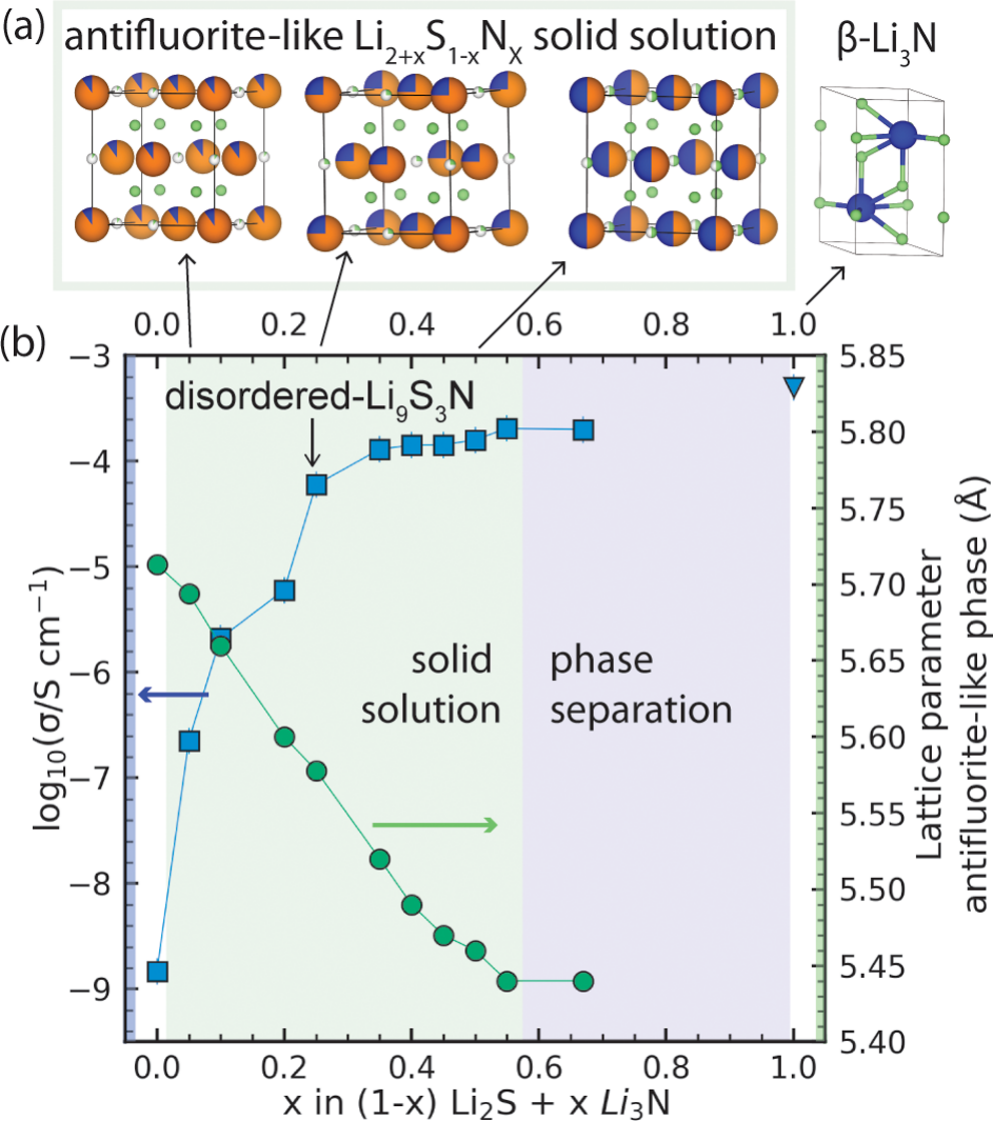
Conductivity of the antifluorite-like solid solution on the (1 – *x*) Li_2_S-*x* Li_3_N tie line (0 < *x* < 0.55). (a) Schematic illustration of the Li_2+*x*_S_1–*x*_N_*x*_ (0 < *x* < 0.55) solid solution and hexagonal β-Li_3_N (b) ionic conductivity and lattice parameter (of antifluorite-like phase) for (1 – x) Li_2_S-*x* Li_3_N samples. Green and blue shading indicates antifluorite-like solid solution (0 < *x* < 0.55) and phase separation (0.55 < *x* < 1) regions, respectively. Li_2_S (*x* = 0) and Li_3_N (*x* = 1) samples were treated with the same ball-milling protocol as the Li_2+*x*_S_1–*x*_N_*x*_ samples for direct comparison. Lines are guides to the eye.

Beyond *x* = 0.55 the lattice parameter ceases to decrease with increasing nitrogen content. The diffraction pattern of an attempted synthesis with nominal stoichiometry 0.33 Li_2_S-0.67 Li_3_N (i.e., *x* = 0.67) showed a phase mixture of Li_3_N and an antifluorite Li_2+*x*_S_1–*x*_N_*x*_ phase (SI Figure S11) with the same lattice parameter as Li_2.55_S_0.45_N_0.55_ indicating that the solubility limit of Li_3_N in Li_2_S is reached at *x* ≈ 0.55. The nitrogen-richest phase in the Li_2+*x*_S_1–*x*_N_*x*_ antifluorite solid solution is thus Li_2.55_S_0.45_N_0.55_.

Figure 6b shows that the conductivity increases with increasing nitrogen content in antifluorite-like Li_2+*x*_S_1–*x*_N_*x*_ (0 < *x* < 0.55). For antifluorite Li_2_S, ball-milled without Li_3_N (i.e., *x* = 0), we measured a room-temperature conductivity of 10^–9^ S cm^–1^. Dissolving a small fraction (*x* = 0.05) of nitrogen into the Li_2_S host structure already improves the room-temperature conductivity by more than 2 orders of magnitude to 2.2 × 10^–7^ S cm^–1^. The conductivity then steadily increases with increasing nitrogen content reaching a high conductivity of 0.22 mS cm^–1^ near the solubility limit at *x* = 0.55. Li_3_N can thus be dissolved in Li_2_S leading to a series of fully reduced solid electrolytes with high ionic conductivities. The conductivity of β-Li_3_N ball-milled in the same way (0.5 mS cm^–1^) is also shown in Figure 6b for comparison—though we note that β-Li_3_N is structurally distinct from the antifluorite-like Li_2+*x*_S_1–*x*_N_*x*_ solid solution.

We measured the conductivity-activation energy of several synthesized Li_2+*x*_S_1–*x*_N_*x*_ phases (*x* = 0.05, 0.1, 0.2, 0.25, 0.45) via impedance spectroscopy at varying temperatures. Figure 7a shows that the experimental activation energy of Li_2+*x*_S_1–*x*_N_*x*_ phases decreases with increasing nitrogen content, suggesting lithium diffusivity at lower energy thresholds. The observation of higher room-temperature conductivity and lower conductivity-activation energy is consistent with our expectation from the analysis of jump-*E*_a_ values and their dependence on the bottleneck composition.

**Figure 7:**
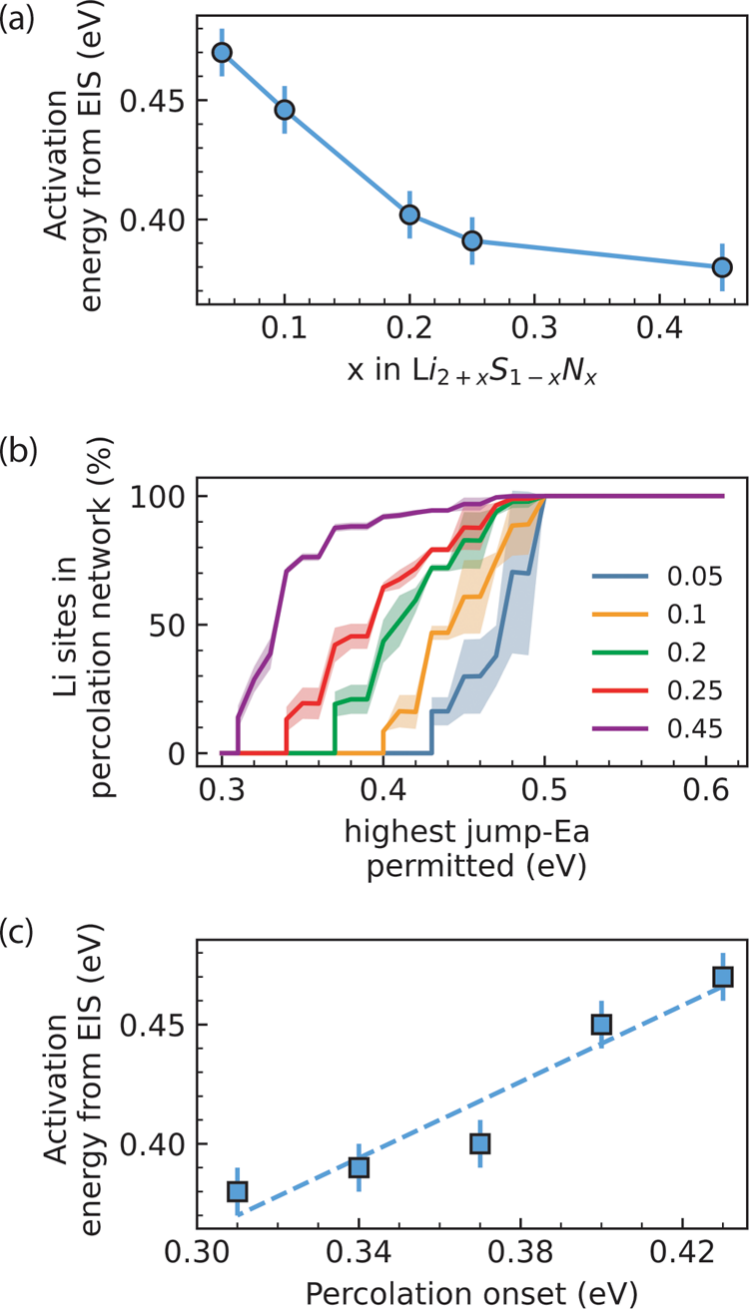
Rationalizing the decreasing activation energy for increased nitrogen content in Li_2+*x*_S_1–*x*_N_*x*_ (0 < *x* < 0.55) antifluorite-like phases. (a) Activation energy obtained from impedance spectroscopy at varying temperatures for phases of the Li_2+*x*_S_1–*x*_N_*x*_ solid solution (i.e., *x* = 0.05, 0.1, 0.2, 0.25, 0.45) (b) Percolation-energy diagram for different phases in the Li_2+*x*_S_1–*x*_N_*x*_ solid-solution. (c) Correlation between the energy of percolation onset and the activation energies from impedance spectroscopy experiments.

We further calculated the corresponding percolation-energy diagrams for the Li_2+*x*_S_1–*x*_N_*x*_ (*x* = 0.05, 0.1, 0.2 0.25, 0.45) phases (Figure 7b) which show increasingly lower energies of percolation onset demonstrating that indeed higher nitrogen content enables diffusion at lower energy thresholds. Taken together with the experimental conductivity results, we arrive at a coherent picture of how nitrogen content in the disordered Li_2+*x*_S_1–*x*_N_*x*_ (0 < *x* < 0.55) solution modulates lithium-ion diffusion by controlling the energy and distribution of local transition states.

Figure 7c highlights the correlation between low-energy percolation onsets and the ability of phases to feature diffusion at low-energy thresholds which is reflected in low experimental conductivity-activation energies. This correlation between the atomistic (Å/nm) simulation results and the macroscopic (mm) experimental results underlines the applicability of the MD-percolation approach to rationalize property-composition relationships in disordered systems.

In conclusion, we demonstrate here a previously unknown partial solid solution in the (1 – *x*)­Li_2_S-*x*Li_3_N tieline, spanning 0 < *x* < 0.55, metastable but accessible by mechanochemistry and crystallizing in antifluorite-like *Fm*3̅*m*. The increasing conductivity with increasing nitrogen content in antifluorite-like Li_2+*x*_S_1–*x*_N_*x*_ (0 < *x* < 0.55) phases can be rationalized by the increased number of low-energy N-rich bottlenecks enabling more percolating lithium-diffusion paths with lower energy thresholds.

### Perspectives for Disordered, Fully Reduced Antifluorite Solid Electrolytes

Solid electrolytes should feature high ionic conductivity and (electro-)­chemical stability against both electrodes. The Li_2+*x*_S_1–*x*_N_*x*_ phases presented here reach ∼0.2 mS cm^–1^ and further improvements of the ionic conductivity may be achieved by further compositional modifications which are very likely possible based on reports nitride-chloride^16,17,23,38^ and phosphide-sulfide^18^ phases with similar antifluorite-like structures, suggesting a large chemical space remaining to be investigated. The Li_2+*x*_S_1–*x*_N_*x*_ phases are structurally analogous to the recently discovered Li_2+*x*_S_1–*x*_P_*x*_ phases which highlights the possibility to substitute phosphide P^3–^ anions (*r* ≈ 1.89 Å, SI Table S7) by significantly smaller N^3–^ anions (*r* ≈ 1.46 Å, SI Table S7). For a given pnictide content (*x*) the Li_2+*x*_S_1–*x*_N_*x*_ phases feature higher conductivities than the Li_2+*x*_S_1–*x*_P_*x*_ phases (see SI Figure S11) possibly because the smaller N^3–^ radii increases the bottleneck diameter.

Regarding (electro-)­chemical stability against electrodes, due to their irreducible nature Li_2+*x*_S_1–*x*_N_*x*_ phases are thermodynamically stable at low potentials down to 0 V vs Li/Li^+^ (see also Supporting Note 11) and thus intrinsically inert to reduction in contact with low-voltage next-generation anodes such as lithium metal or silicon. Indeed we demonstrate in Figure 8a stable lithium stripping/deposition in Li/Li_2.55_S_0.45_N_0.55_/Li cells over hundreds of hours.

**Figure 8:**
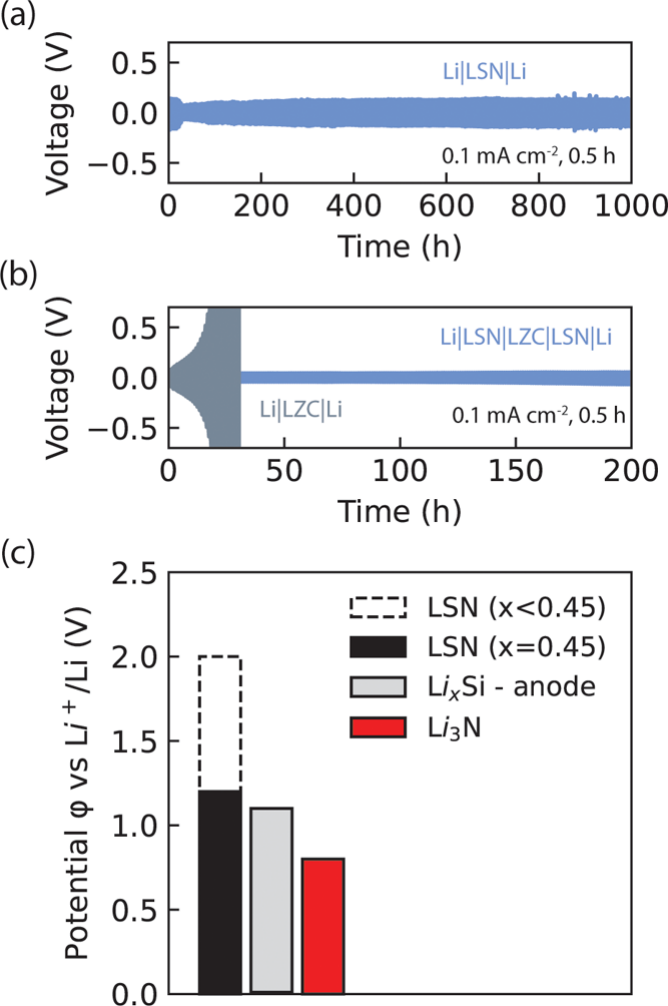
Electrochemical characterization of Li_2+*x*_S_1–*x*_N_*x*_ (0 < *x* < 0.55) phases. (a) Symmetric Li/Li_2.45_S_0.45_N_0.55_/Li cell cycled at 0.1 mA cm^–2^, 0.5 h of plating and stripping. (b) Symmetric Li/Li_2.45_S_0.45_N_0.55_/Li_2_ZrCl_6_/Li_2.45_S_0.45_N_0.55_/Li and symmetric Li/Li_2_ZrCl_6_/Li cells. Catastrophic voltage increase of Li/Li_2_ZrCl_6_/Li cells is inhibited by protection against Li-metal with a Li_2+*x*_S_1–*x*_N_*x*_ phase. (c) Black: Stability window of the of Li_2+*x*_S_1–*x*_N_*x*_ phases, increasing with nitrogen content. Red: Stability window of Li_3_N. Gray: Potential window over which Li_*x*_Si (*silicon*) anodes operate.

While irreducible, based on our preliminary linear-sweep voltammetry experiments shown in SI Figure S12 the Li_2+*x*_S_1–*x*_N_*x*_ phases can be oxidized at modest voltages. For low nitrogen content the anodic limit lies close to 2 V (vs Li/Li^+^) which is close to the anodic limit of Li_2_S. With increasing nitrogen content, while the ionic conductivity increases, the anodic limit decreases to ∼1.25 V (for *x* = 0.45). These low anodic limits are still significantly higher than the anodic limit of Li_3_N of ca. 0.8 V vs Li/Li^+^ (SI Figure S12). While equally inert to reductive decomposition, the increased oxidative stability may be a key advantage of antifluorite-like Li_2+*x*_S_1–*x*_N_*x*_ (0 < *x* < 0.55) phases over Li_3_N to enable next-generation anodes.

For example, considering that the operation window of Li_*x*_Si anodes ranges from 0.01 to 1.1 V, Li_3_N would oxidize against Li_*x*_Si anodes due to its low anodic limit of ∼0.8 V vs Li (Figure 8c). In contrast, antifluorite-like Li_2+*x*_S_1–*x*_N_*x*_ phases would be more suitable protection layers against Li_*x*_Si anodes as they would be inert to reduction **and** oxidation against Li_*x*_Si anodes. We thus envisage that Li_2+*x*_S_1–*x*_N_*x*_ and related irreducible electrolytes could be applied as anolytes or protective layers against low-potential anodes, in conjunction with a catholyte. As a proof of concept, we demonstrate in Figure 8b and in Supporting Note 11 that Li_2+*x*_S_1–*x*_N_*x*_ (0 < *x* < 0.55) phases may be used to prevent catastrophic decomposition of the Li_2_ZrCl_6_ solid electrolyte against lithium metal electrodes. These examples demonstrate that inertness to reduction is not the sole suitability criterion of anolytes and highlights the potential of new highly conducting irreducible phases for enabling next-generation solid-state batteries.

## Conclusions

In this study we report the discovery of a new family of irreducible solid electrolytes by dissolving lithium nitride into the antifluorite Li_2_S resulting in crystalline Li_2+*x*_S_1–*x*_N_*x*_ (0 < *x* < 0.55) lithium-rich antifluorite phases reaching high conductivities >0.2 mS cm^–1^ at room temperature. Leveraging a thorough diffusion-percolation analysis, we develop a widely applicable analysis approach and clarify the mechanism by which compositional disorder unlocks high conductivities in these solid electrolytes.

Using the examples of ordered and disordered-Li_9_S_3_N we demonstrate how the rich diversity of coordination-environment compositions creates new lithium sites and bottlenecks, enabling low-energy-percolating diffusion pathways. In particular, we identify the composition of the bottlenecks as highly correlated with the local jump-activation energy, with nitride-rich bottlenecks favoring diffusion. Based on this observation we endeavor to maximize nitrogen content, in the process discovering a solid solution of Li_2+*x*_S_1–*x*_N_*x*_ (0 < *x* < 0.55) antifluorite electrolytes reaching 0.2 mS cm^–1^ for the maximum nitrogen composition of Li_2.55_S_0.45_N_0.55_. Through our combined experimental­(EIS)-computational­(AIMD-percolation) analyses on Li_2+*x*_S_1–*x*_N_*x*_ we establish that nitrogen-richer compositions indeed show higher conductivities and lower conductivity-activation energies that can be rationalized by lower percolation onset energies due to the increased occurrence of low jump-activation energy, nitrogen-rich bottlenecks.

The new Li_2+*x*_S_1–*x*_N_*x*_ (0 < *x* < 0.55) electrolytes we discovered are irreducible, thus thermodynamically stable against all known next-generation low-potential anode materials for batteries. Their high conductivities and stability at low potentials make these new electrolytes a natural choice for much needed anolytes and protection layers combination with low-potential, high-capacity anodes such as silicon or lithium-metal. Most relevantly, our results shed light on the mechanism by which structural disorder can affect ionic conductivity in solids.

Empirical correlations between structural disorder and ion conduction in solid electrolytes have long been observed but these correlations have been system dependent and their causality has been hardly understood on a local/atomistic level. The mantra of “the more disorder the better” has led to recent research trends into ever more compositionally complex (so-called *high-entropy)* solid electrolytes. While this strategy might be a way to improve ionic conductivity by way of structural frustration, we show that high compositional complexity is not necessary to access disorder-mediated improvements in ionic conductivity. Instead, by comparing disordered Li_9_S_3_N and Li_6_PS_5_Br argyrodite to their ordered counterparts of the same composition, we demonstrate how stabilizing metastable configurations at a fixed stoichiometry of compositionally simple (*low entropy*) materials—e.g., through mechanochemistry, quenching or soft-chemical approaches—can be sufficient to effect dramatic ionic conductivity improvements without invoking compositional complexity.

We expect that the methodology we introduce here will prove useful in rationalizing the diffusivity in other systems exhibiting structural disorder—both positional as demonstrated here but also orientational in the case of orientational glasses and rotor phases.

## Methodology

### Synthesis

#### Ordered-Li_9_S_3_N

We closely followed the procedure described by Miara and co-workers.^22^ A planetary ball mill jar with 10 mm ZrO_2_ balls and a ball/powder ratio of 30 at 550 rpm for 99 (5 min-milling-5 min-pause) cycles was used to mix the precursors Li_2_S and Li_3_N. (Here the ball-milling step is utilized to intimately mix the reagents and does not lead to anion disorder in the final product after annealing). Subsequently the powder mixture was transferred to tungsten crucibles and sealed into quartz glass ampules under 200 mbar of argon. The ampules were then heated (100 °C/h) to 600 °C held at this temperature for 24 h and then slowly (over the course of 24 h) cooled down to RT. All preparation steps were performed in an argon atmosphere (H_2_O < 1 ppm, O_2_ < 1 ppm).

#### Disordered Li_2+*x*_S_1–*x*_N_*x*_ Phases

The synthesis precursors were Li_2_S (Sigma-Aldrich, 99%) and Li_3_N (Sigma-Aldrich, >99.5%). Stoichiometric amounts of the precursors were milled in a planetary ball mill (Jar: ZrO_2_, 45 mL) with 10 mm ZrO_2_ balls and a ball:powder mass ratio of 30 at 550 rpm for 99 (5 min milling-5 min-pause) cycles. The powdered materials were used for further characterization as milled.

### Electrochemical Characterization

Electrochemical impedance spectroscopy (EIS): Pellets (diameter = 10 mm) of the Li_2+*x*_S_1–*x*_N_*x*_ probes were pressed (3.2 tons) in custom-made solid-state lab cells. These lab cells consist of an alumina tube and two stainless steel plungers. Solid electrolyte powder is filled in the alumina tube and compressed on both sides with the stainless steel plungers. The cell configuration used was SS| Li_2+*x*_S_1–*x*_N_*x*_ |SS (SS = stainless steel). AC impedance was performed with a Metrohm Autolab (AUT86298) in the frequency range 10 MHz to 0.1 Hz with a voltage amplitude of 10 mV. Linear sweep voltammetry (LSV): LSV measurements were also performed with a Metrohm Autolab (AUT86298). To measure the anodic limit of Li_2+*x*_S_1–*x*_N_*x*_ phases, Li| Li_2+*x*_S_1–*x*_N_*x*_ | Li_2+*x*_S_1–*x*_N_*x*_ –C cells were used. To make the Li_2+*x*_S_1–*x*_N_*x*_–C composite cathode a mixture of Li_2+*x*_S_1–*x*_N_*x*_:Super P with a weight ratio of 0.7:0.3 was milled in a planetary ball mill (Jar: ZrO_2_, 45 mL) with 10 mm ZrO_2_ balls and a ball/powder ratio of 30 at 400 rpm for 2 h (5 min milling; 5 min pause). Li| Li_2+*x*_S_1–*x*_N_*x*_ | Li_2+*x*_S_1–*x*_N_*x*_–C cells were assembled by pressing a Li_2+*x*_S_1–*x*_N_*x*_ pellet (130 mg, 3.2 tons) and subsequently the Li_2+*x*_S_1–*x*_N_*x*_–C composite (15 mg, 3.2 tons) on top of it. Finally, a Li disk was placed on the opposite side of the Li_2+x_S_1–*x*_N_*x*_ pellet. The LSV scanning rate was 0.01 mV s^–1^. Conductivity measurements at different temperatures for Arrhenius fits*:* SS| Li_2+*x*_S_1–*x*_N_*x*_|SS cells were kept at 30 °C for 1h, then heated in 5 min to 50 °C and kept at this temperature for 30 min followed by heating to 60 °C in 5 min and maintaining the temperature for 30 min. This procedure was continued up to 100 °C. The EIS obtained at the end of the 30 min temperature-plateaus were used for Arrhenius fits.

### X-ray Diffraction

Powder diffraction patterns were collected using Cu Kα X-rays (1.54 Å) on a PANalytical X’Pert Pro X-ray diffractometer in Bragg–Brentano (*reflection*) geometry up to a 2θ_max_ ≈ 80° (*q*_max_ ≈ 5.2 Å^–1^). The air sensitive Li_2+*x*_S_1–*x*_N_*x*_ probes were loaded into airtight holders in an Ar-filled glovebox prior to the measurements. GSAS-II^39^ and FullProf^40^ (through the user interface implemented in the “Match!” software) were used for LeBail and Rietveld refinements.

### Neutron Diffraction

Neutron powder diffraction data were collected on the PEARL neutron powder diffractometer at the research reactor of TU Delft.^41^ Approximately 4 g of samples were loaded on 6 mm diameter cylindrical vanadium holders and sealed using indium wire under Ar atmosphere. Measurements were collected of the powder samples at room temperature with a neutron wavelength of 1.667 Å selected using the 533 reflection of a Ge monochromator, in transmission geometry up to a 2θ_max_ ≈ 155° (*q*_max_ ≈ 7.3 Å^–1^).

### Computational Details

All DFT calculations were performed with the Vienna ab initio simulation package (VASP) with computational settings consistent with those used in the Materials Project database.^42^ For the generation and analysis of supercells the calculations were done on 2 × 2 × 2 Li_2+*x*_S_1–*x*_N_*x*_ supercells. Because of the shared site occupations and partial occupancies in Li_2+*x*_S_1–*x*_N_*x*_ phases different atomic arrangements were generated by random decoration of the Wyckoff 4a (0,0,0) position with nitrogen and sulfur and the 4b (0.5,0.5,0.5) positions were randomly decorated with Li and vacancies. The Wyckoff 8c (0.25,0.25,0.25) position was fully occupied with Li for all stoichiometries. For the generation and analysis of supercells the pymatgen package was used.^43^ For the AIMD simulations the Li pseudopotential was changed from Li_sv (which was used for relaxations) to Li as this enables the use of a lower energy cutoff. The simulation time was >200 ps for every AIMD simulation. The AIMD simulations were executed at 900 K. The dissection of AIMD simulations into individual jump events and subsequent analysis of jump frequencies and individual *E*_a,Jump_ values was done as first described by de Klerk and Wagemaker;^26^ a comprehensive account can be found in ref 26 but crucial aspects for the understanding of the reported data is presented here: Partitioning of the supercell volume into site and nonsite voxels: The lithium site centers are obtained from crystallography and the site radii are set to the average vibrational amplitude of the Li-ions as described in ref 26, which amounts to ca. 0.44 Å for the present simulations. Given the site center and site radius, spherical sites are defined around the site-center. Calculation of *E*_a,jump_ values between two sites: The sites are defined around the 0 K equilibrium positions of the Li ions. At every simulation step it is recorded in which site each Li ion is located or whether it is currently between two sites. From this information the jump frequency between two site v_A→B_ can be calculated according to eq 2

2 $$v_{A\to B}=\frac{N_{A\to B}}{\tau_{A}}$$

where v_A→B_ is the jump frequency for jumps from site A to site B, *N*_A→B_ is the number of recorded jumps from A to B, and τ_A_ is the time of occupation of site A. *E*_a,jump_ is then obtained from eq 1. The uncertainty on the average jump-*E*_a_ value for a jump type can be obtained from the standard deviation of the mean (ε_mean_) and the uncertainty associated with convergence (ε_convergence_, as further detailed in Supporting Note 6) so that the total uncertainty on average jump-*E*_a_ values is ε_jump‑ea_ = ε_mean_ + ε_convergence_ and is typically on the range of 10–30 meV. This whole analysis is strongly supported by the gemdat (ref 44) python package currently developed in our group.

Percolation model: We performed AIMD simulations on 8 selected supercells that in sum contained all jump events present in the disordered Li_2+*x*_S_1–*x*_N_*x*_ phases (incl. disordered-Li_9_S_3_N); this enabled the construction of a *jump library* with an average jump-*E*_a_ value for each jump event, shown in SI Tables S5 and S6. The jump events for ordered-Li_9_S_3_N were obtained from an AIMD simulation of and ordered-Li_9_S_3_N supercell. Subsequently a percolation analysis could be performed on 50 (5 × 5 × 5) supercells for each of the different Li_2+*x*_S_1–*x*_N*_x_* stoichiometries (*x* = 0.05,0.1, 0.2, 0.25,0.3, 0.4, 0.5, 0.6, 0.7, 0.8, 0.9). The percolation analysis works as follows: An activation energy cutoff is defined. Two sites A and B are connected if a randomly picked element in the range [jump-*E*_a(A→B)_ – uncertainty, jump-*E*_a(A→B)_ + uncertainty] and a randomly picked element in the range [jump-*E*_a(B→A)_ -uncertainty, jump-*E*_a(B→A)_ + uncertainty] are below the activation energy cutoff. In this way a graph can be constructed which we did using the rustworkX package ref 45 If a path extends throughout the supercell the path is considered percolating, provided that the end point of the percolation path is equally a starting point of a percolating path (see SI Figure S6). For a given supercell and a given energy cutoff the analysis needs to be repeated until the average fraction of sites in the percolation network converges. In cases where only a subset of supercells were percolating at a given cutoff the average fraction of percolating sites was obtained from percolating supercells. The standard deviation of the distribution of fractions at one cutoff energy was taken as the uncertainty on the fraction of active Li sites. Bottleneck size calculations: 50 disordered Li_9_S_3_N supercells were relaxed (containing >9000 bottlenecks) to account for local distortions which may not be present in long-range averaged crystallographic unit cells. The three atoms at the vertices of triangular bottlenecks connecting sites were identified and the inner-circle diameter using the sympy Triangle package. For the calculation of bottleneck diameters the biangle line (line that “cuts an angle in half”) was followed by the distance of the ion-radius of the ion located at the vertex. This was done at all three vertices so that a new triangle is formed. The outer-circle diameter of this new triangle is determined by the sympy (ref 46) Triangle package and is the bottleneck diameter. For each type of bottleneck (i.e., NSS, NNS···) the average diameter is determined and the standard deviation of the distribution of diameters is shown as the error bar.

## Supplementary Material



## Data Availability

The data that support the findings of this study and the code to reproduce the results shown in the paper are openly available in 4TU.ResearchData at 10.4121/f3632023-c54e-4c95-848b-3e4db819bbf7. We used python version 3.10 and the following python packages: numpy,^47^ gemdat,^44^ matplotlib,^48^ pymatgen,^43^ rustworkx,^45^ sympy^46^

## Footnotes

1 We note that the “active” versus “inactive” nomenclature used here, is conceptually comparable to the ‘accessible’ and ‘inaccessible’ nomenclature used in the disordered rock-salt cathode literature.^77^

## Notes

Additional experimental details, analysis and results, including powder diffractograms and associated analysis; tabulated jump-activation energies used in percolation analysis; linear sweep voltammetry data; estimation of the amorphous fraction in mechanochemically synthesized samples; analysis of the lithium distribution in disordered Li_9_S_3_N; investigation into the correlation of ion hops in disordered-Li_9_S_3_N; estimation of the attempt frequency ν_0_; discussion of the conceptual differences between jump-activation energies and energy barriers; uncertainty analysis on jump-*E*_a_ values; comparison of the jump-*E*_a_ values between ordered- and disordered-Li_9_S_3_N; additional discussion on the interpretation of percolation-energy diagrams; application of MD-percolation methodology on Li_6_PS_5_Br; discussion on the measured Arrhenius prefactor evolution of Li_2+*x*_S_1–*x*_N_*x*_; characterization of chemical and electrochemical stability of Li_2+*x*_S_1–*x*_N_*x*_ (PDF)
