# Supplementary material for: Disorder-Mediated Ionic Conductivity in Irreducible Solid Electrolytes
Source: J Am Chem Soc. 2025 May 26;147(22):18840–52. doi: 10.1021/jacs.5c02784 (PMC12147116; doi:10.1021/jacs.5c02784)
Supplement: Supplementary file 1 [file ja5c02784_si_001.pdf]

Supporting Information for

Disorder-mediated ionic conductivity in irreducible solid electrolytes

Victor Landgraf, Mengfu Tu, Wenxuan Zhao, Anastasia K. Lavrinenko, Zhu Cheng, Jef Canals, Joris de Leeuw, Swapna Ganapathy, Alexandros Vasileiadis , Marnix Wagemaker*, Theodosios Famprikis*

Faculty of Applied Sciences, Delft University of Technology, 2629 JB Delft, The Netherlands

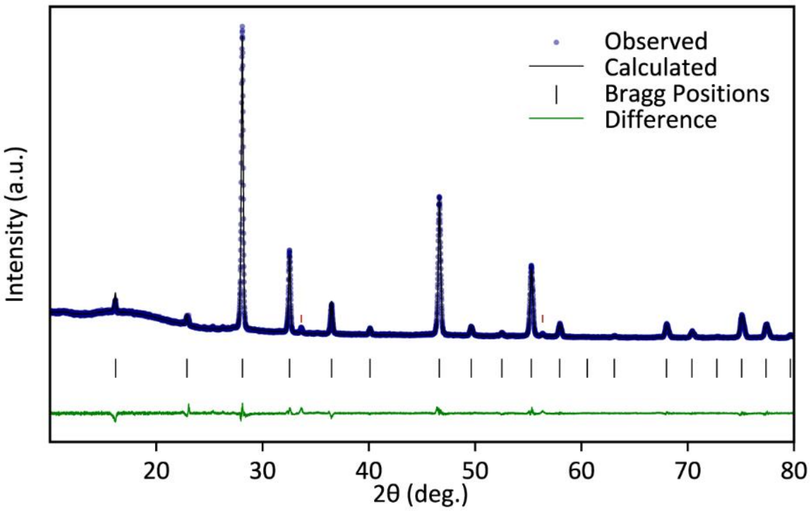

Figure S1. X-ray diffraction pattern of ordered- Li_9S_3N with a Rietveld fit based on the structure solution proposed in ref. [1] A small Li_2O impurity is present (~3 wt. %) indicated by red lines. Associated structural model in Table S1 and refinement details in Table S2.

Table S1: Structure solution for ampoule-synthesized ordered-Li_9S_3N. Fitted parameters in ***bold***. *Standard uncertainties multiplied by Berar's correction*[2] *in parentheses.* Space group: $\boldsymbol{Pm\overline{3}m}$, lattice parameter **a = 5.5187(3) Å**

Atom	x	y	z	Wyckoff	Occupancy	U_{iso} (Å^2)
Li1	**0.2136(12)**	**0.2136(12)**	**0.2136(12)**	8g	1	**0.004(4)**
Li2	½	½	½	1b	1	**0.040(12)**
N	0	0	0	1a	1	**0.0022(10)**
S	0	½	½	3c	1	**0.0022(10)**

Table S2. Details of Rietveld Structure Refinement of ordered-Li_9S_3N from X-ray Diffraction Measurements at 300K

empirical formula	Li_9S_3N
T/K	300
fw/ g mol^{-1}	172.7
space group (no.)	$Pm\bar{3}m$ (221)
unit cell params/ Å	a = 5.5187(3)
Z	1
V/Å^3	168.073(9)
ρ_{calc}/g cm^{-3}	1.706
diffracted beam	x-rays
λ/ Å	1.54187
2ϑ range/deg	23.76-80.00
R_p	4.27%
R_{wp}	6.23%
R_{exp}	3.73%
χ^2	2.79
GOF	1.67
R_{Bragg}	17.43%
R_f	7.61%
depository no.	CDC-2426484

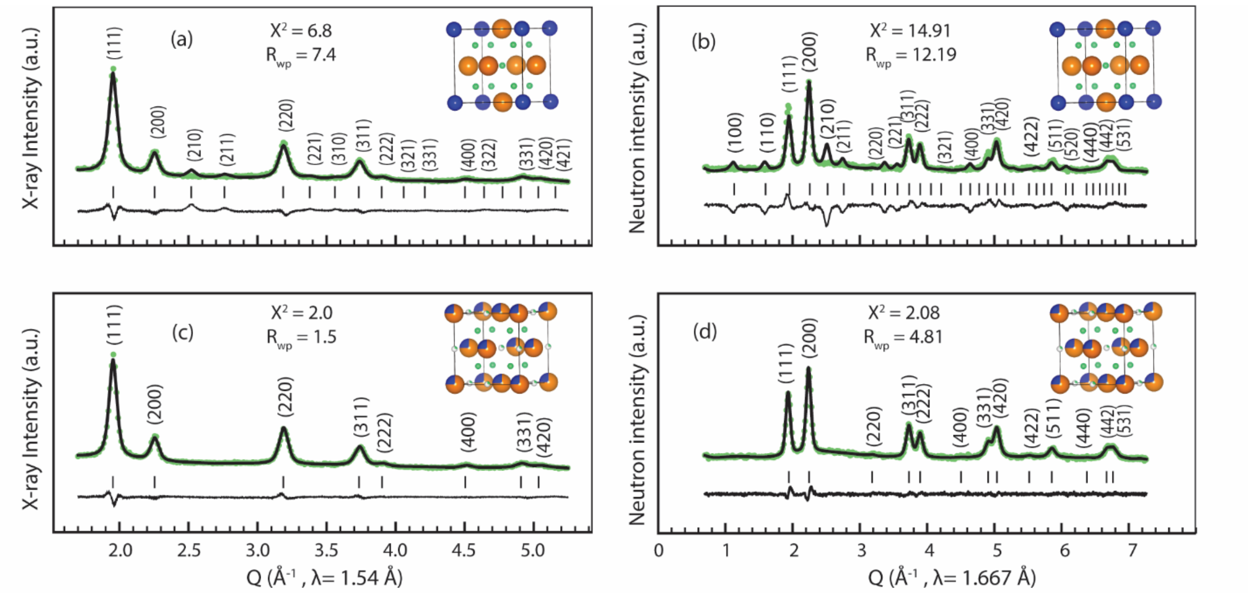

Figure S2. Powder diffraction on mechanochemically prepared Li_9S_3N. (a) X-ray diffraction pattern of mechanochemically prepared Li_9S_3N with an attempt to fit the $Pm\overline{3}m$ -Li_9S_3N crystal solution proposed by Marx et al. [1]. (b) Neutron diffraction pattern of mechanochemically prepared Li_9S_3N with an attempt to fit the $Pm\overline{3}m$ -Li_9S_3N crystal solution proposed by Marx et al. [1] (c) Same X-ray diffraction pattern as in (a) but now with the Rietveld fit of an improved structure solution with the higher-symmetry $Fm\overline{3}m$ space group (d) Same Neutron diffraction pattern as in (b) but now with the Rietveld fit of an improved structure solution with the higher-symmetry $Fm\overline{3}m$ space group. The vertical lines indicate the position of the Bragg reflections. Inset structural models with Li, S, N in green, orange, blue, respectively.

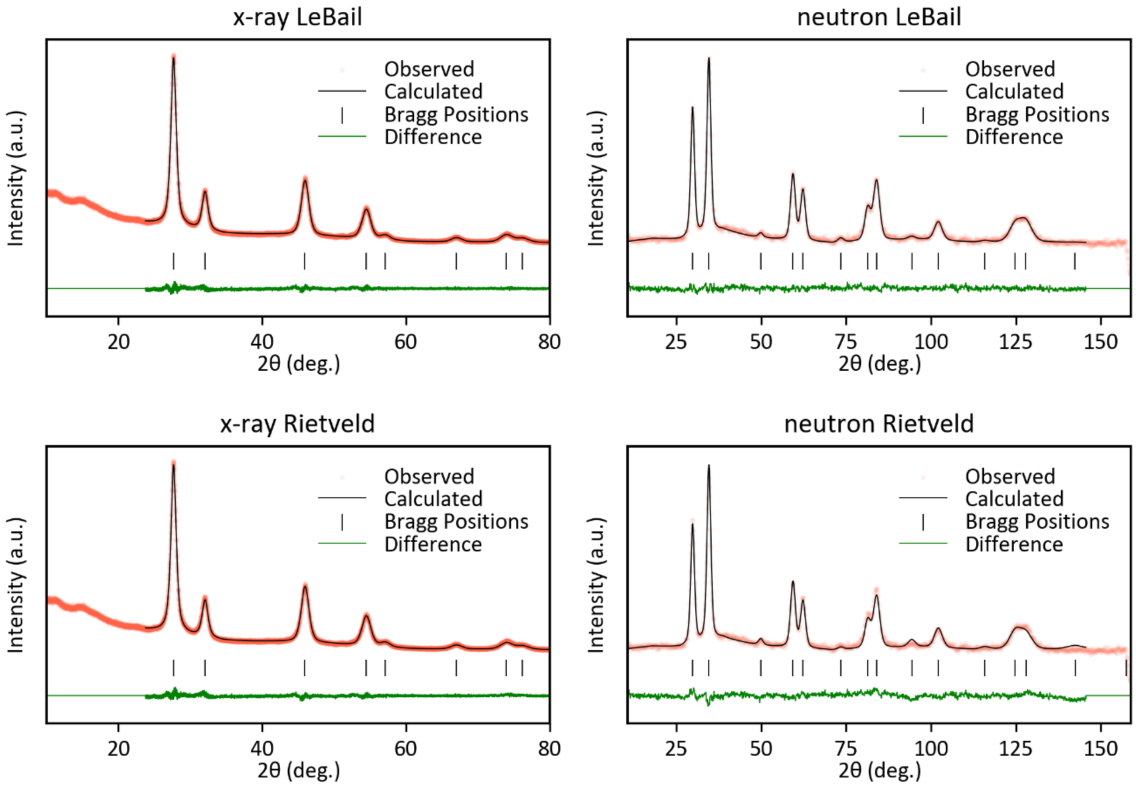

Figure S3. Combined fits of x-ray (Cu Kα) and neutron (λ=1.667 Å) diffractogram of disordered Li_9S_3N. LeBail x-rays wR_p = 4.70, GoF= 1.26 —LeBail neutron wR_p = 3.99, GoF= 1.25 — Rietveld x-ray wR_p = 4.91, GoF= 1.32 — Rietveld neutron wR_p = 5.19, GoF= 1.62. Associated structural model in Table S3 and refinement details in Table S4.

Table S3: Refined structure solution for $Fm\bar{3}m$-Li_9S_3N obtained from the combined Rietveld refinement of neutron and X-ray diffraction patterns shown in Figure S3. Fitted parameters in ***bold***. *Standard uncertainties in parentheses.* Lattice parameter **a = 5.5846(4) Å**

Atom	x	y	z	Wyckoff	Occupancy	U_{iso} (Å^2)
S	0	0	0	4a	¾	**0.0331(3)**
N	0	0	0	4a	¼	**0.0132(5)**
Li-tet	¼	¼	¼	8c	1	**0.0760(7)**
Li-oct	½	½	½	4b	¼	**0.55(2)**

Table S4. Details of Joint Rietveld Structure Refinement of disordered-Li_9S_3N from Neutron and X-ray Diffraction Measurements at 300K

empirical formula	$Li_{2.25}S_{0.75}N_{0.25}$	
T/K	300	
fw/ g mol^{-1}	43.17	
space group (no.)	$Fm\bar{3}m$ (225)	
unit cell params/ Å	a = 5.5846(1)	
Z	4	
V/Å^3	174.173(4)	
ρ_{calc}/g cm^{-3}	1.646	
diffracted beam	neutrons	x-rays
λ/ Å	1.667	1.54187
2ϑ range/deg	10.60-145.69	23.76-80.00
R_p	4.23%	3.77%
R_{wp}	5.19%	4.91%
R_{exp}	3.20%	3.72%
χ^2	2.62	1.74
GOF	1.62	1.32
R_{Bragg}	10.51%	4.48%
R_f	4.72%	2.25%
depository no.	CDC-2426483	

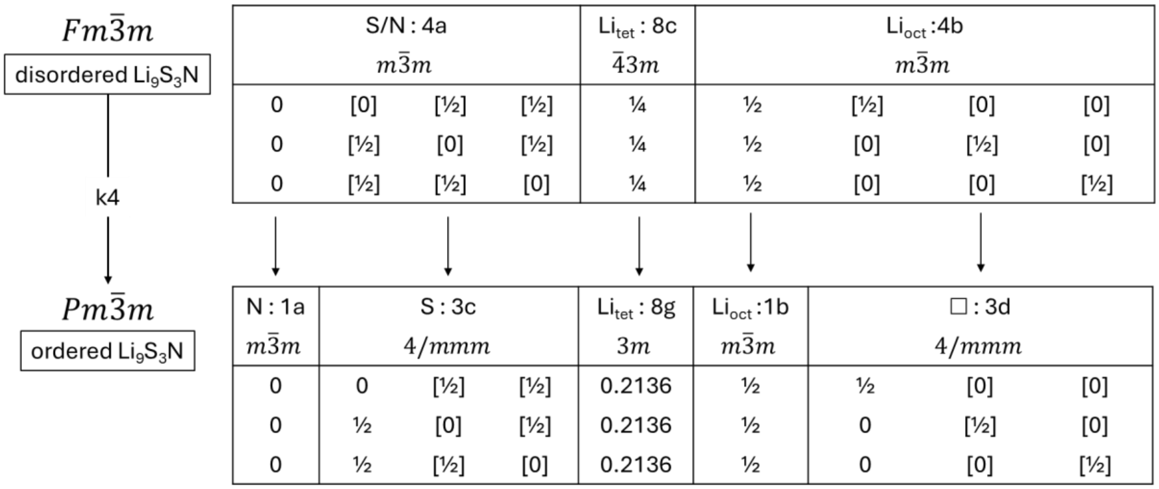

Figure S4: Bärnighausen tree of the group-subgroup relationship between ordered and disordered Li_9S_3N

Table S5. Average jump-Ea values used for anion-disordered $Li_{2+x}S_{1-x}N_x$ phases, their uncertainty ($\varepsilon_{jump-Ea}$) and the ε_{mean} and $\varepsilon_{convergence}$ values which are comprised in $\varepsilon_{jump-Ea}$. Additionally the table lists how many times each jump type is represented in the 8 supercells an how many jumps were recorded for each jump type.

Jump type	Average jump-Ea (eV)	ε_{mean} (eV)	$\varepsilon_{convergence}$ (eV)	$\varepsilon_{jump-Ea}$ (eV)	Occurrence of jump type	Number of jump events
S_6-N_1S_3(SSS)	0.417	0.013	0.014	0.027	49	397
N_4S_2-N_4(NNN)	0.2	0.018	0.024	0.042	3	44
N_3S_1-N_5S_1(NNS)	0.309	0.017	0.009	0.026	26	199
N_3S_1-N_1S_3(NS)	0.449	0.018	0.02	0.038	15	55
N_4-N_3S_1(NN)	0.357	0.015	0.014	0.029	31	146
N_1S_3-N_1S_3(SS)	0.494	0.007	0.01	0.017	52	73
N_5S_1-N_3S_1(NNN)	0.28	0.012	0.008	0.02	9	105
N_1S_3-N_3S_1(NS)	0.452	0.023	0.026	0.049	15	61
$0N_3S_3$-N_4(NNN)	0.157	0	0.011	0.011	1	36
S_4-N_1S_3(SS)	0.535	0.011	0.005	0.016	14	18
N_3S_1-N_4(NN)	0.359	0.012	0.009	0.021	31	139
N_3S_1-N_2S_4(NNS)	0.308	0.013	0.023	0.036	29	690
N_2S_4-N_2S_2(NSS)	0.274	0.008	0.021	0.028	73	1312
N_4S_2-N_2S_2(NSS)	0.266	0.016	0.003	0.019	16	273
S_4-S_6(SSS)	0.504	0.01	0.029	0.039	39	105
N_3S_1-N_3S_1(NN)	0.367	0.007	0.009	0.016	92	794
N_1S_3-N_4S_2(NSS)	0.342	0.028	0.012	0.04	9	155
N_2S_2-N_3S_3(NNS)	0.367	0.01	0.009	0.019	86	1213
S_4-S_4(SS)	0.538	0.012	0.019	0.031	13	16
N_1S_3-N_1S_5(NSS)	0.423	0.009	0.007	0.016	83	692
S_6-S_4(SSS)	0.366	0.024	0.042	0.066	42	99
N_1S_3-N_2S_4(NSS)	0.399	0.008	0.002	0.01	140	1541
N_3S_1-N_3S_1(NS)	0.415	0.011	0.008	0.019	48	165
N_2S_2-N_2S_4(NSS)	0.333	0.008	0.008	0.016	69	1318
N_4S_2-N_3S_1(NNN)	0.268	0.011	0.019	0.031	26	373
N_3S_1-N_4S_2(NNS)	0.291	0.01	0.018	0.028	61	1531
N_4-N_6(NNN)	0.316	0.013	0.016	0.029	64	317
N_3S_1-N_6(NNN)	0.348	0.027	0.02	0.047	8	37
N_3S_1-N_3S_3(NNN)	0.408	0.022	0.025	0.046	6	38
N_3S_3-N_2S_2(NSS)	0.271	0.008	0.022	0.03	68	1148
N_2S_2-N_2S_4(NNS)	0.365	0.012	0.01	0.022	29	356
N_2S_2-N_3S_3(NSS)	0.329	0.007	0.018	0.025	68	1151
N_2S_2-N_2S_2(NS)	0.464	0.005	0.011	0.016	164	532

N_2S_4-N_1S_3(SSS)	0.421	0.016	0.019	0.036	22	84
N_1S_3-N_2S_2(SS)	0.513	0.01	0.006	0.015	13	14
N_5S_1-N_3S_1(NNS)	0.288	0.013	0.006	0.019	25	211
N_2S_2-N_1S_3(NS)	0.467	0.009	0.023	0.032	78	286
N_2S_2-N_2S_2(SS)	0.451	0.023	0.028	0.051	12	45
N_3S_1-N_5S_1(NNN)	0.303	0.031	0.047	0.078	9	113
N_1S_3-S_6(SSS)	0.425	0.014	0.02	0.035	48	394
N_3S_3-N_2S_2(NNS)	0.28	0.01	0.028	0.037	88	1233
N_1S_3-N_3S_3(SSS)	0.48	0.028	0.04	0.068	4	10
N_4-N_4(NN)	0.331	0.008	0.008	0.016	106	482
N_2S_2-S_4(SS)	0.479	0.001	0.051	0.051	1	2
S_4-N_2S_4(SSS)	0.494	0.011	0.013	0.025	24	81
N_5S_1-N_2S_2(NNS)	0.299	0.02	0.028	0.047	5	19
N_3S_3-N_3S_1(NNS)	0.239	0.01	0.038	0.049	47	1014
N_4S_2-N_3S_1(NNS)	0.22	0.008	0.034	0.042	62	1541
N_1S_5-N_1S_3(NSS)	0.301	0.011	0.028	0.039	87	748
N_3S_3-N_1S_3(NSS)	0.254	0.008	0.02	0.028	74	977
N_4S_2-N_1S_3(NSS)	0.299	0.022	0.009	0.032	8	89
N_3S_3-N_1S_3(SSS)	0.393	0.012	0.004	0.016	5	17
N_4-N_5S_1(NNN)	0.307	0.014	0.011	0.026	27	135
N_4-N_2S_2(NN)	0.361	0.021	0.024	0.045	3	9
N_2S_2-N_5S_1(NNS)	0.295	0.049	0.005	0.054	4	18
N_4-N_3S_3(NNN)	0.243	0.004	0.009	0.013	1	42
N_2S_4-N_2S_2(NNS)	0.243	0.015	0.045	0.06	29	366
N_1S_3-N_2S_4(SSS)	0.459	0.019	0.015	0.034	20	79
N_1S_3-N_3S_3(NSS)	0.358	0.008	0.008	0.017	71	961
N_1S_3-N_1S_5(SSS)	0.427	0.01	0.009	0.019	67	455
N_1S_3-N_2S_2(NS)	0.472	0.009	0.027	0.035	85	299
N_2S_2-N_4S_2(NSS)	0.346	0.019	0.011	0.029	15	263
N_2S_2-N_4S_2(NNS)	0.334	0.011	0.017	0.028	41	609
N_1S_5-N_1S_3(SSS)	0.361	0.01	0.003	0.013	67	467
N_6-N_4(NNN)	0.231	0.009	0.035	0.044	59	309
N_2S_4-N_1S_3(NSS)	0.272	0.008	0.023	0.032	142	1343
N_4-N_4S_2(NNN)	0.341	0.027	0.026	0.054	5	49
S_4-N_2S_2(SS)	0.568	0	0	0.065*	1	1
N_1S_5-N_2S_2(NSS)	0.274	0.016	0.044	0.06	43	981
N_2S_4-S_4(SSS)	0.329	0.02	0.005	0.025	21	89
N_4S_2-N_2S_2(NNS)	0.265	0.008	0.007	0.015	40	607
N_3S_3-N_3S_1(NNN)	0.337	0.024	0.016	0.041	5	42
N_1S_5-S_4(SSS)	0.318	0.011	0.006	0.017	54	248
N_3S_1-N_2S_2(NS)	0.453	0.008	0.019	0.027	79	225

N_3S_1-N_2S_2(NN)	0.399	0.016	0.012	0.028	32	287
N_2S_2-N_2S_2(NN)	0.383	0.012	0.016	0.028	46	584
N_3S_1-N_4S_2(NNN)	0.332	0.015	0.016	0.031	26	372
N_6-N_3S_1(NNN)	0.265	0.025	0.031	0.056	7	27
S_4-N_1S_5(SSS)	0.489	0.011	0.041	0.052	57	254
N_2S_2-N_3S_1(NN)	0.402	0.016	0.011	0.028	31	262
N_5S_1-N_4(NNN)	0.273	0.015	0.02	0.035	27	142
N_2S_4-N_3S_1(NNS)	0.26	0.011	0.026	0.038	27	609
N_1S_3-N_1S_3(NS)	0.489	0.005	0.021	0.027	145	444
N_2S_2-N_1S_5(NSS)	0.31	0.011	0.017	0.028	42	980
S_4-N_3S_3(SSS)	0.472	0.03	0.02	0.05	2	8
N_1S_3-S_4(SS)	0.505	0.012	0.013	0.025	15	22
N_3S_1-N_3S_3(NNS)	0.307	0.01	0.019	0.029	47	1029
N_3S_3-S_4(SSS)	0.416	0	0.018	0.018	1	3
N_2S_2-N_1S_3(SS)	0.507	0.014	0.011	0.025	13	21
N_2S_2-N_3S_1(NS)	0.457	0.009	0.026	0.035	67	204

Table S6. Average jump-Ea values used for anion-ordered Li_9S_3N phases and their uncertainty ($\varepsilon_{jump\text{-}Ea}$) and the ε_{mean} and $\varepsilon_{convergence}$ values which are comprised in $\varepsilon_{jump\text{-}Ea}$.

Jump type	Average jump-Ea (eV)	ε_{mean} (eV)	$\varepsilon_{convergence}$ (eV)	$\varepsilon_{jump\text{-}Ea}$ (eV)
N_1S_3-N_2S_4(NSS)	0.431	0.002	0.003	0.005
N_2S_4-N_1S_3(NSS)	0.245	0.002	0.002	0.004
N_1S_3- S_6(SSS)	0.482	0.003	0.009	0.011
S_6- N_1S_3(SSS)	0.483	0.003	0.008	0.010
N_1S_3-N_1S_3(SS)	0.590*	n/a	n/a	0.015
N_1S_3-N_1S_3(NS)	0.472	0.002	0.009	0.011

* For N_1S_3-N_1S_3(SS) jump-type no jump was observed in the AIMD of ordered-Li_9S_3N. If one jump had occurred the jump-Ea would be 0.575 eV so that we know jump-Ea >0.575. We set the jump-Ea to 0.59 and accord an uncertainty of 0.015 which regarding the magnitude of uncertainties of the other jump-types is a cautious estimate.

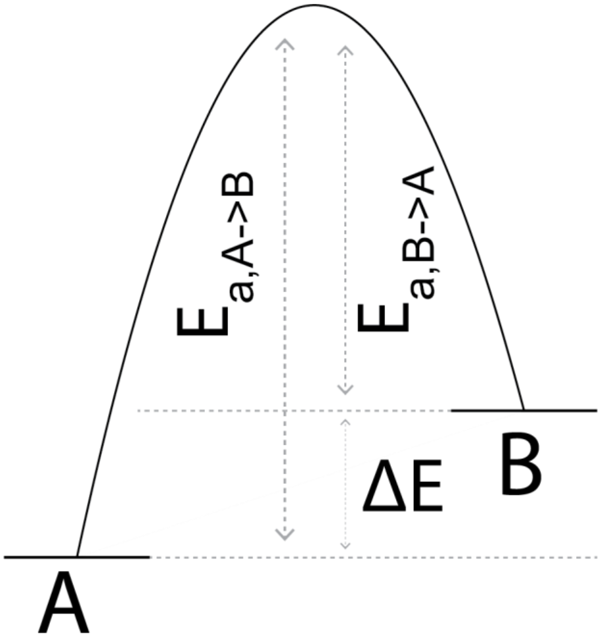

Figure S5. Schematic of the energy profile for a Li ion jump from site A to site B. This figure demonstrates that if $E_{a,\ A\text{->}B}$ > $Ea_{,\ B\text{->}A}$ then site A is more stable than site B by ΔE.

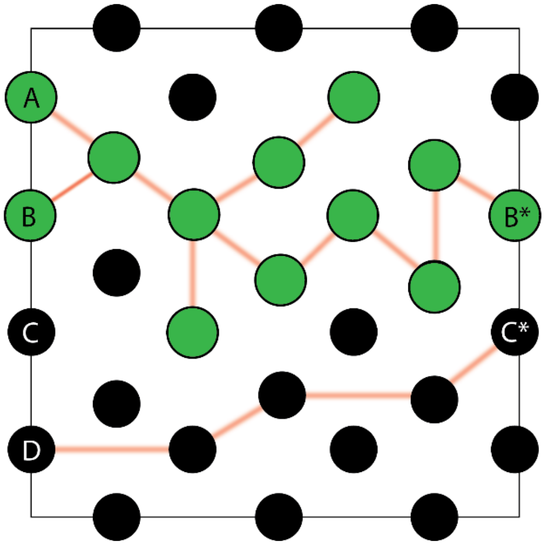

Figure S6. Schematic illustrating the percolation model used in this study. We define a path as percolating if it connects sites throughout one full side length of the (5x5x5) supercells and provided that the end-point of percolation is also the starting point of a percolating path. For example, in the schematic above the path leading from site A to site B* is percolating because site B is a starting point of a percolating path. In contrast, the path from D to C* is not percolating because C is not starting point of a percolating path. In this way periodic boundary conditions are respected. The green highlighting shows all sites which are connected to percolation paths.

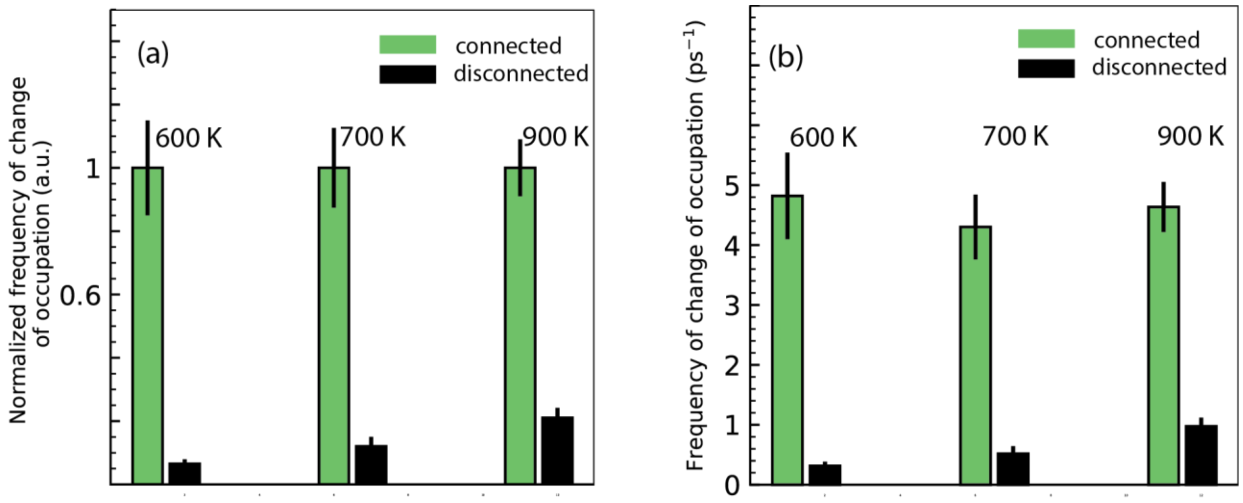

Figure S7. (a) Normalized frequency of occupation change for AIMD simulations at different temperatures for the same disordered-Li_9S_3N supercell. The connected sites were obtained with a jump-Ea cutoff of 0.4 eV. It becomes apparent that the frequency of occupation-change is much larger for connected sites than for disconnected sites. This discrepancy is more prominent at lower temperatures and at 300K likely even more pronounced than at 600 K. (b) Same as in (a) but not normalized. The frequency of occupation-change is obtained by tracking the occupation of sites throughout the AIMD simulation and the number of changes (i.e. change from one Li to another Li or from Li to vacancy) of occupation for individual sites.

Table S7. Ionic Radii used in this investigation for example for the calculations for Figure 5. All ionic radii are taken from ref [3] except for the ionic radius of phosphide ions P(-III) which was estimated to be ~1.89 Å from the average Li-P distance in Li_3P taken from the structure proposed in ref [4].

Atom	Oxidation state	Coordination number	Ionic Radius (Å)
Li	1	4	0.59
N	-3	4	1.46
S	-2	6	1.84
P	-3	5	~1.89

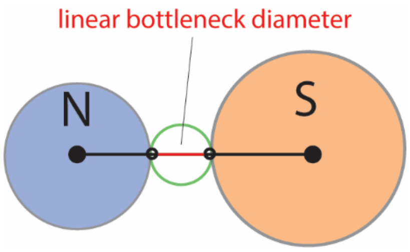

Figure S8. Illustration of the geometrical consideration to calculate the bottleneck diameter for linear bottlenecks between tet-tet jumps.

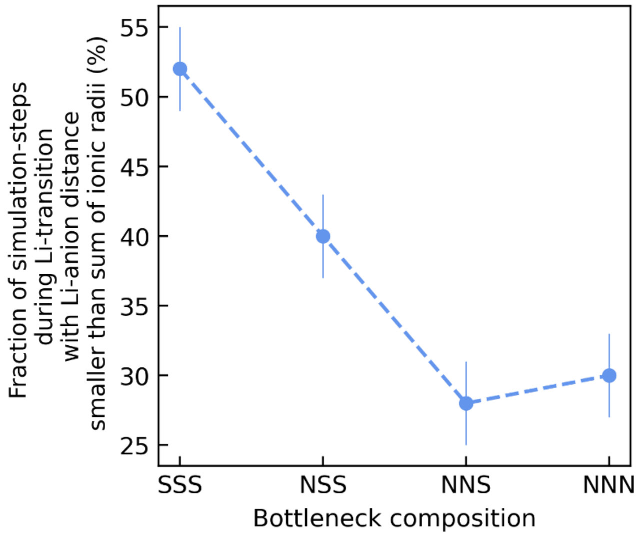

Figure S9. Correlation between bottleneck composition and the time spent during ion jumps (ion-"transitions") at distances short of the sum of the ionic radii which are energetically unfavourable, obtained from an AIMD simulation of a $Li_{2.25}S_{0.75}N_{0.25}$ supercell. The dotted line is a guide to the eye.

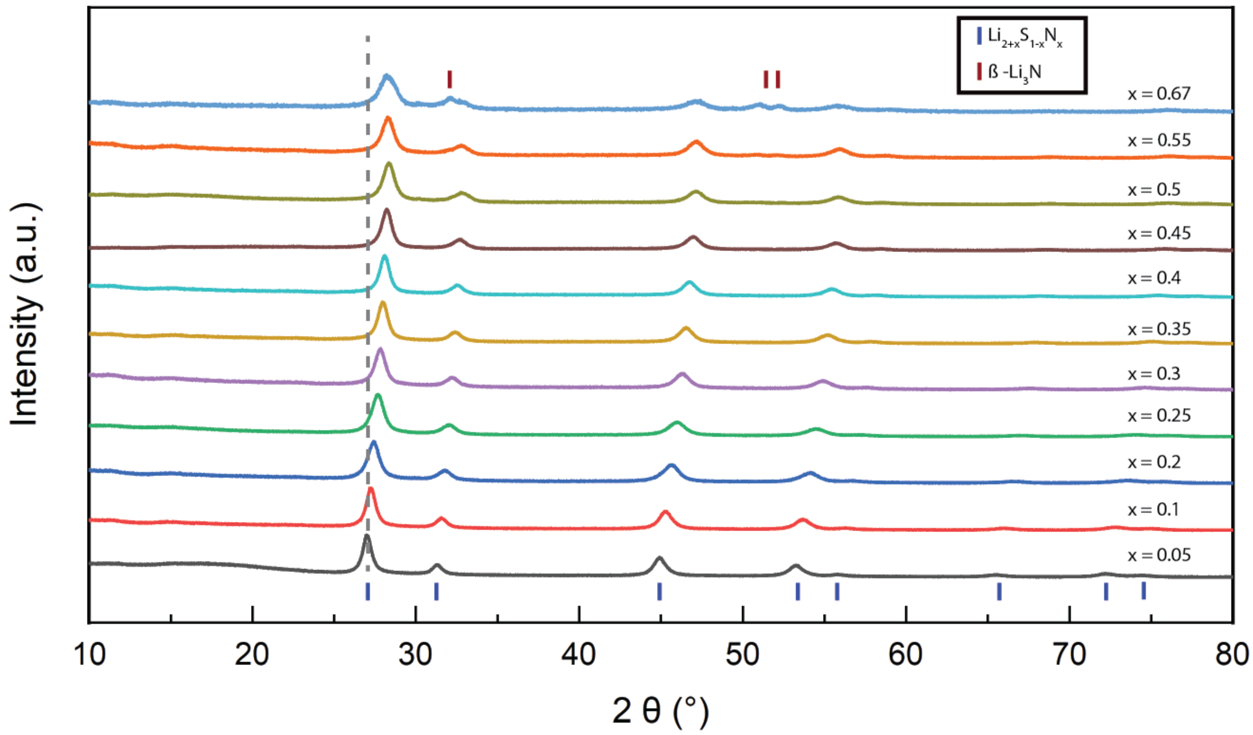

Figure S10. X-ray diffraction patterns of mechanochemically treated (1-x)Li_2S-xLi_3N samples for 0.05<x<0.67. Diffraction peaks for Fm-3m antifluorite-like $Li_{2+x}S_{1-x}N_x$ observed throughout. The grey vertical line is a guide to the eye to better visualize the peak shifts due to a decreasing lattice parameter with increasing nitrogen content. A synthesis attempted with the formal composition $Li_{2.67}S_{0.33}N_{0.67}$ resulted in a phase mixture of β-Li_3N and an $Li_{2+x}S_{1-x}N_x$ with the same lattice parameter as $Li_{2.55}S_{0.45}N_{0.55}$ indicating that x=0.55 is the solubility limit of nitrogen in in the $Li_{2+x}S_{1-x}N_x$ phases.

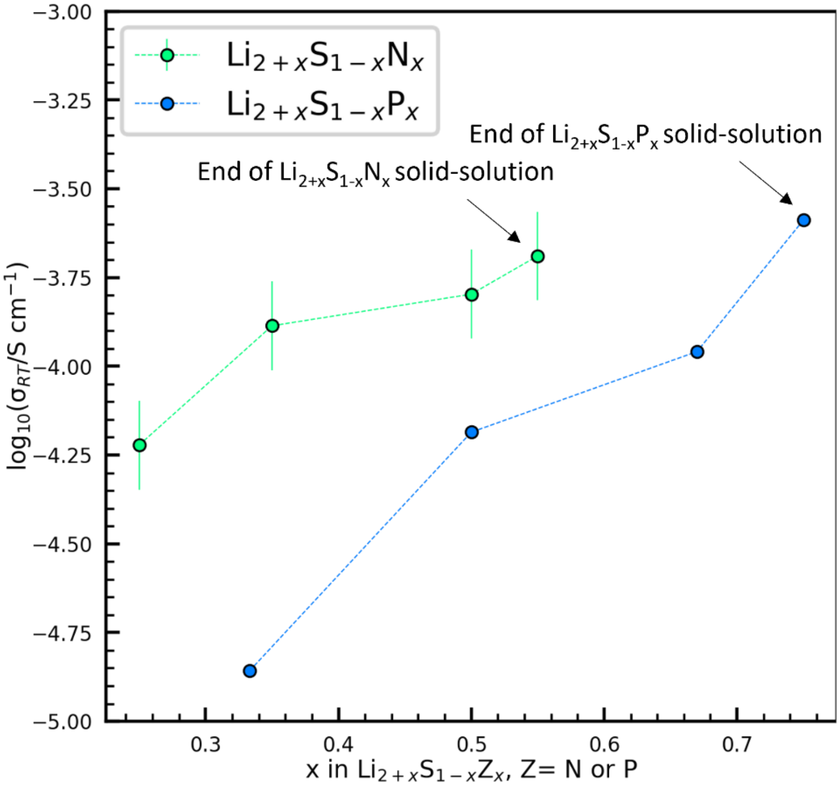

Figure S11. Comparison of the $Li_{2+x}S_{1-x}N_x$ and $Li_{2+x}S_{1-x}P_x$ solid-solutions. Data for the latter from Sczuka and coworkers. [5] For a given pnictide content x the $Li_{2+x}S_{1-x}N_x$ phases shows higher conductivities by factor ~3. The $L_{2i+x}S_{1-x}P_x$ solid solution extends to higher pnictide content likely because of the better ion-size match between P (~1.89 Å) and S (1.84 Å) than between S and N (1.46 Å).[3]

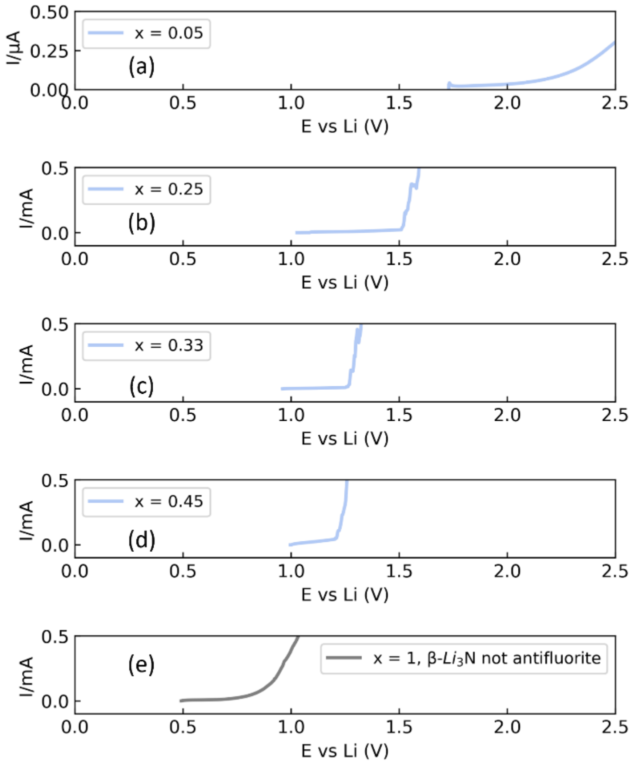

Figure S12. (a-d) Oxidative LSV sweeps of Li|LSN|LSN-C cells for LSN phases with different N content. (LSN: $Li_{2+x}S_{1-x}N_x$) (e) oxidative LSV sweep of a Li|Li_3N| Li_3N -C cell. It can be seen that the anodic limit of LSN phases decreases with increasing N content. For comparison the anodic limit of Li_3N was also measured. It can be seen that the oxidation limit of LSN phases is higher than that of Li_3N by more than 0.4 V.

Supporting Note 1: Estimation of amorphous fraction in mechanochemically-synthesized $Li_{2+x}S_{1-x}N_x$ phases

High-energy ball milled samples inherently result in products with small particle sizes which results in diffraction peak broadening and potential amorphization. This may result in obscured impurities in high-energy ball-milled samples. This section aims to estimate the fraction of amorphous phases and impurities by (1) using a crystalline Si standard and (2) annealing the samples so that they become fully crystalline. To investigate the amorphous fraction introduced by synthesis approach used for the $Li_{2+x}S_{1-x}N_X$ phases we further investigated the $Li_{2.2}S_{0.8}N_{0.2}$ as a representative of these phases.

The amorphous fraction was estimated as follows. 320 mg of mechanochemically-synthesized $Li_{2.2}S_{0.8}N_{0.2}$ and 42 mg of crystalline Si powder (Sigma 7440-21-3) were thoroughly hand-ground together. Rietveld refinements of the diffraction pattern (Figure S13) enable to estimate the weight ratio of crystalline $Li_{2.2}S_{0.8}N_{0.2}$ and crystalline Si.

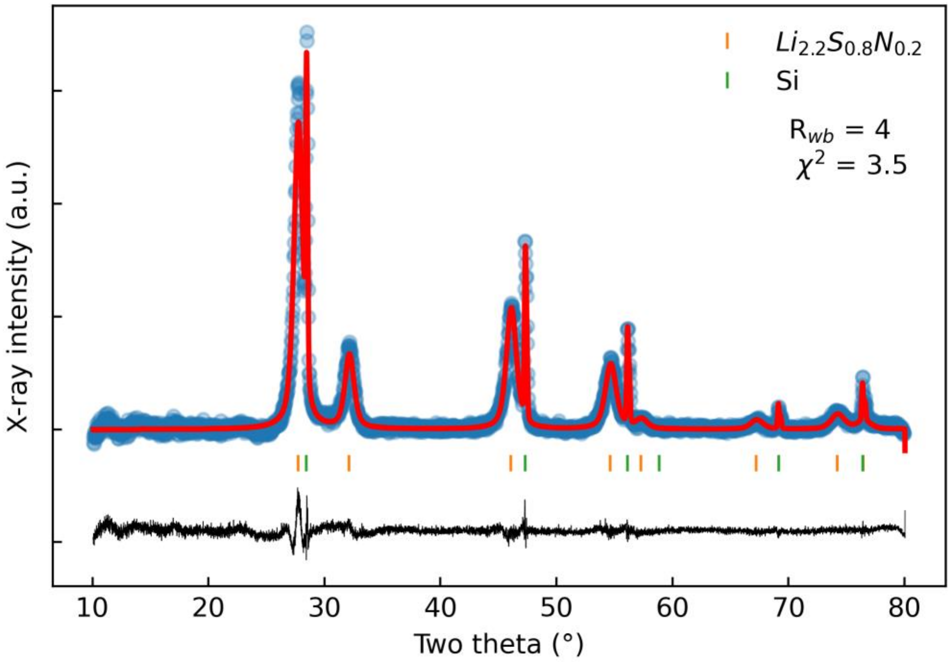

Figure S13. Rietveld refinement of mechanochemically-synthesized $Li_{2.2}S_{0.8}N_{0.2}$ with a crystalline Si standard. The $w_{Li2.2S0.8N0.2}$ /w_{Si} ratio is 7.62.

Since the amount of crystalline Si is known, the amorphous fraction of $Li_{2.2}S_{0.8}N_{0.2}$ may be determined. From the refined weight ratio of crystalline $Li_{2.2}S_{0.8}N_{0.2}$ and crystalline Si powder a table of the elemental composition can be obtained as:

$$\kappa = \frac{w_{S,sample} M_{Si}}{w_{Si,sample} M_S} \quad (S1)$$

$$a_f = \left(\kappa - \frac{w_{S,refinement} M_{Si}}{w_{Si,refinement} M_S} \right) * \frac{1}{\kappa} \quad (S2)$$

In the equations above a_f is the amorphous fraction, w_S and w_{Si} are the weight fractions of S and Si respectively (shown in Table S8) and M_{Si} and M_S are the molar weights of S and Si respectively.

Table S8. Elemental composition obtained from Rietveld refinement with crystalline Si standard

	Wt % Li	Wt % S	Wt % N	Wt % Si
Nominal	0.320	0.492	0.072	0.116
Calculated from refinement	0.318	0.490	0.071	0.120

Based on the above assumption a maximum amorphous fraction of the order of 4.3 wt% is determined.

To investigate whether amorphous impurities are present we heated the $Li_{2.2}S_{0.8}N_{0.2}$ samples to 600 °C for 48 h and cooled them slowly (20 °C h^{-1}) to room temperature. The resulting phases are the thermodynamically stable ordered-Li_9S_3N (which can also be written as ordered- $Li_{2.25}S_{0.75}N_{0.25}$) and Li_2S. This partial decomposition of the $Li_{2.2}S_{0.8}N_{0.2}$ into these two thermodynamic products is coherent with our understanding that the $Li_{2+x}S_{1-x}N_x$ phases are metastable. A small Li_2O impurity is also found (~3 wt. %). Besides no impurity could be identified. The Rietveld refinement of the diffraction pattern of the mechanochemically synthesized and annealed $Li_{2.2}S_{0.8}N_{0.2}$ is shown in Figure S14.

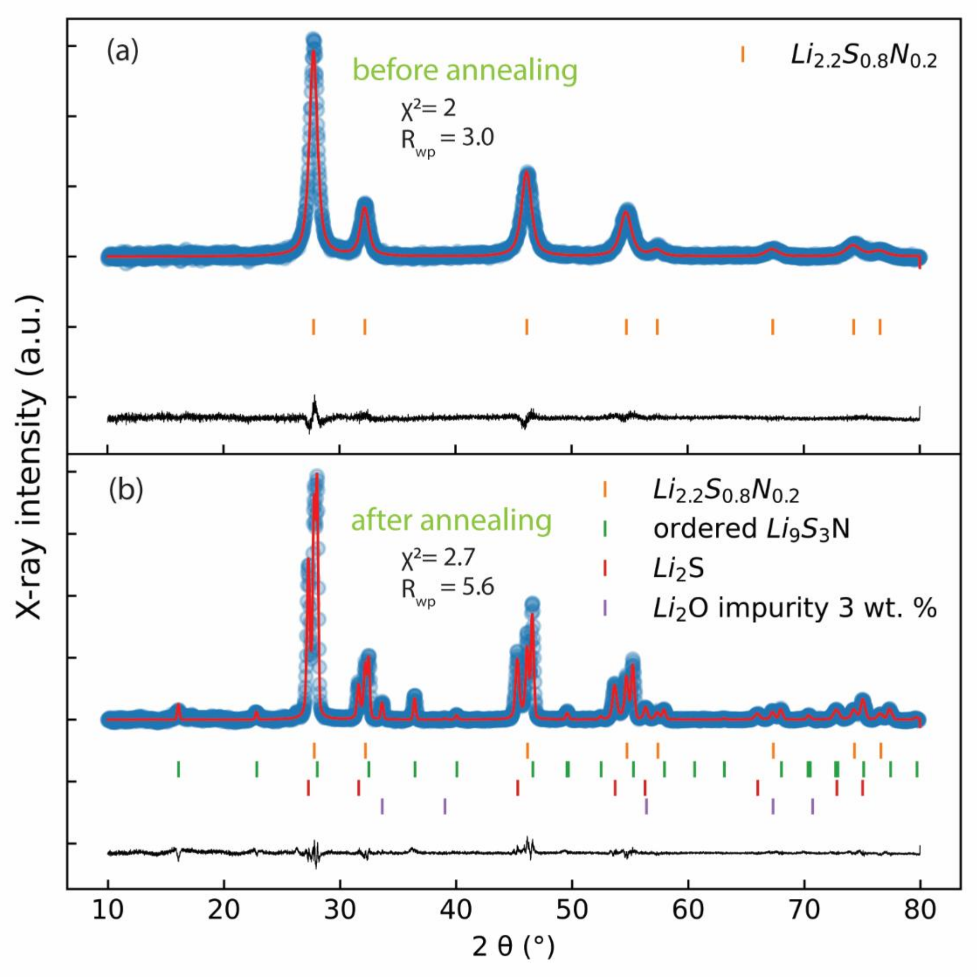

Figure S14. a) Rietveld refinement of pure $Li_{2.2}S_{0.8}N_{0.2}$ b) Rietveld refinement of mechanochemically-synthesized $Li_{2.2}S_{0.8}N_{0.2}$ heated to 600 °C for 2 h and cooled down slowly (20 °C h^{-1})

Supporting Note 2: Analysis of the lithium distribution in disordered-Li_9S_3N

The structure solution that we propose for disordered-Li_9S_3N feature large thermal parameters (see Table S3 with U_{iso} >0.5 Å^2 and >0.07 Å^2 for octahedral and tetrahedral sites). These U_{iso} values are larger than the ones for the Pm-3m ordered-Li_9S_3N phase which has U_{iso} values <0.04 Å^2 for tetrahedral and octahedral sites (see Table S1).

In the following we address why such large thermal parameters were necessary to obtain good Rietveld refinements for the disordered Li_9S_3N phase and we demonstrate that good Rietveld fits may be obtained with split-sites for the disordered Li_9S_3N phase which does not necessitate large thermal parameters and where the split-site coordinates are coherent with observations from molecular dynamics trajectories (vide infra).

Large U_{iso} values may either capture the large thermal displacements within a site (*dynamic disorder*) or a displacive relaxation away from the crystallographic centre due to local ordering (*static disorder*). To establish whether the large U_{iso} originate from large thermal displacements around the sites we studied the thermal motion of Li around its sites with AIMD. To study the thermal displacements of Li-ions around their equilibrium site positions we performed AIMD simulations at 300 K on two disordered-Li_9S_3N (2x2x2) supercells. We performed the simulations at 300 K to eliminate jump-events during simulations. The average square displacement of Li ions from their equilibrium site position may be used to estimate the U_{iso} value of sites. Figure S15 shows U_{iso} values estimated in this way for different tetrahedral and octahedral sites.

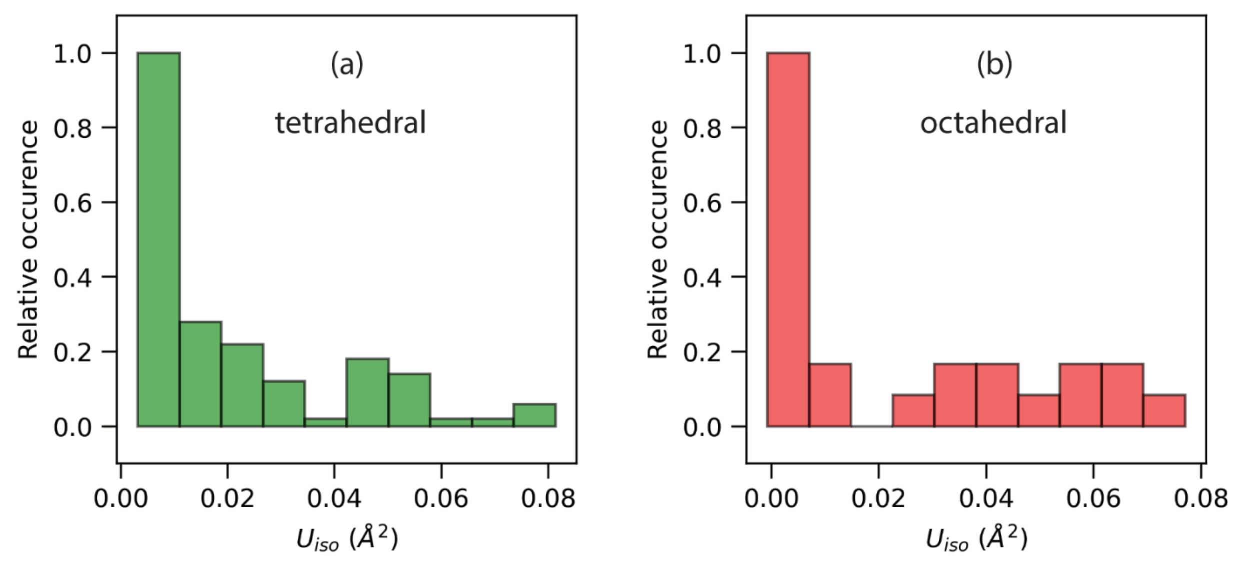

Figure S15. (a) U_{iso} values calculated from AIMD simulations at 300 K for different tetrahedral sites in disordered Li_9S_3N supercells. (b) U_{iso} values calculated from AIMD simulations for different octahedral sites in disordered Li_9S_3N supercells.

Figure S15 shows that if the U_{iso} values in the Rietveld refinements only captured thermal displacements, they should be significantly smaller than the values which were necessary for good Rietveld refinements with the structure solution proposed in Table S3.

The above argument suggests that the large U_{iso} values capture static disorder of the Li sites. We study the exact Li positions by analysing radial distribution functions of our AIMD simulations. The Li-anion radial distribution function of Figure S16 shows that Li is on average closer to N^{3-} than to S^{2-} anions. The closer proximity of Li ions and N^{3-} anions suggests that Li sites may be displaced from the centre of coordination polyhedra (i.e. tetrahedra and octahedra) towards N^{3-} anions. The N-N , S-S and S-N RDFs overlay entirely which underpins that it is clearly Li sites displaced from the centre of polyhedra towards N^{3-} rather than N^{3-} displaced from the Wyckoff (0,0,0) positions.

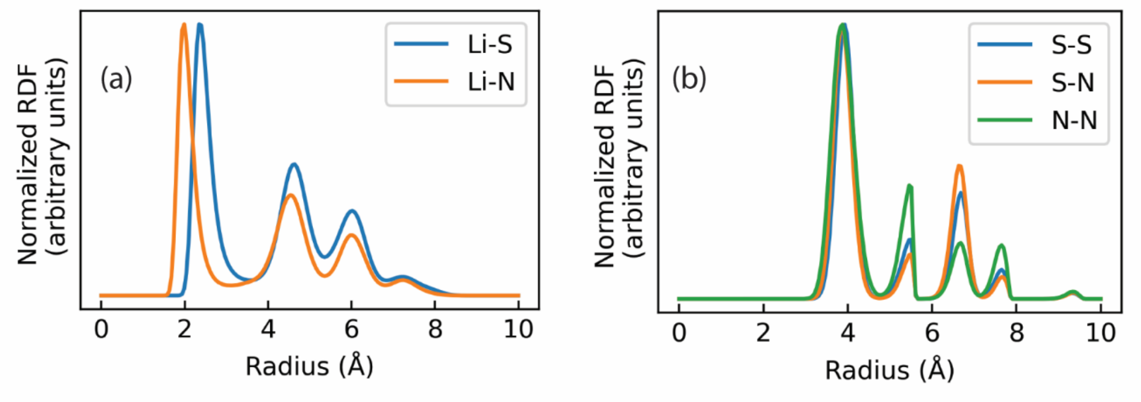

Figure S16. Radial distribution function (RDF) from an AIMD of a $Li_{2.25}N_{0.25}S_{0.75}$ supercell at 300 K. (a) Li-N and Li-S RDF. (b) N-N, N-S, S-S RDF.

Based on the above findings the large U_{iso} values needed for good Rietveld refinements may be explained as follows. The presence of different polyhedra in disordered Li_9S_3N cause displacements of the Li-sites away from the centre of polyhedra towards the surrounding N^{3-} anions. Good Rietveld refinements were obtained for disordered-Li_9S_3N with Li-sites at the centre of polyhedra and large U_{iso} values.

Based on the above observations, an alternative model is proposed, based on refinement against the neutron diffractogram which entails a 6-fold splitting of the octahedral site (Wyckoff 4b, (½, ½, ½)) in the [100] direction, i.e. Wyckoff 24e (x, ½, ½). This model, presented in Figure S17 and Table S9, results in a reasonable fit of the neutron diffractogram and a modest value of U_{iso} for the lithium sites, namely 0.0420 $Å^2$ which is altogether reasonable for the mobile ion in an ion conductor. The shift along the [100] is consistent with the observation from MD of the tendency of Li to displace towards the nitride ions, and the resulting shortest Li-N distance of ca. 2 Å is reasonable considering the ionic radii of N^{3-} and Li^{+}.

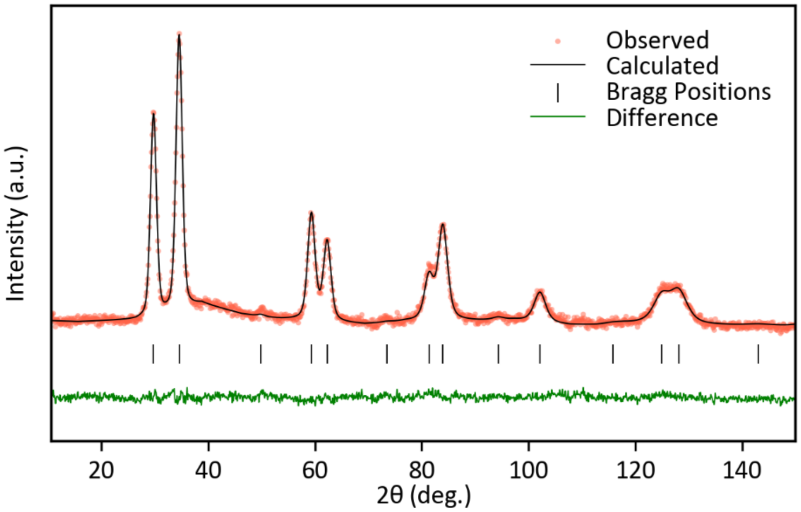

Figure S17: Rietveld fit of neutron diffractogram of disordered Li_9S_3N. λ=1.667 Å, *wRp* = 4.47%, *GoF*= 1.37. Associated structural model in Table S9 and refinement details in Table S10.

Table S9: Refined structure solution for $Fm\overline{3}m$-Li_9S_3N obtained from the Rietveld refinement of neutron pattern shown in Figure S3. Fitted parameters in ***bold***. *Standard uncertainties multiplied by Berar's correction*[2] *in parentheses.* Lattice parameter a = **5.5846(9)** Å

Atom	x	y	z	Wyckoff	Occupancy	U_{iso}
S	0	0	0	4a	**0.761(10)**	**0.0229(12)**
N	0	0	0	4a	**0.239(10)**	**0.0229(12)**
Li-tet	¼	¼	¼	8c	1	**0.042(4)**
Li-oct	½	½	½	4b	**0.15(6)**	**0.042(4)**
Li-oct-split	**0.64(7)**	½	½	24e	**0.015(11)**	**0.042(4)**

Constraints (occ: occupancy, U: U_{iso}, mult:multiplicity)

- occ[N] + occ[S] = 1
- occ[Li-tet] = 1 (*freely fitting led to values slightly >1)*
- U[N] = U[S]
- U[Li-tet] = U[Li-oct] = U[Li-oct-split]
- 2*occ[N]*mult[N] + 3*occ[S]*mult[S] = occ[Li-tet]*mult[Li-tet] + occ[Li-oct]*mult[Li-oct] + occ[Li-oct-split]*mult[Li-oct-split] (*electroneutrality*)

Table S10. Details of Rietveld Structure Refinement of disordered-Li_9S_3N from Neutron and X-ray Diffraction Measurements at 300K

empirical formula	$Li_{2.25}S_{0.75}N_{0.25}$
T/K	300
fw/ g mol^{-1}	43.17
space group (no.)	$Fm\bar{3}m$ (225)
unit cell params/ Å	a = 5.5846(9)
Z	4
V/Å^3	174.17(3)
ρ_{calc}/g cm^{-3}	1.651
diffracted beam	neutrons
λ/ Å	1.667
2ϑ range/deg	10.59-156.19
R_p	3.57%
R_{wp}	4.47%
R_{exp}	3.26%
χ^2	1.88
GOF	1.37
R_{Bragg}	13.16%
R_f	4.61%
depository no.	CSD-2426482

Supporting Note 3: Correlation of ion hops in disordered-Li_9S_3N

It was shown for some high-conductivity ion conductors, such as Li_6PS_5Cl (ref.[6]) and $Li_{10}GeP_2S_{12}$ (ref.[7]), that lithium hops do not occur independently but in a correlated fashion. Additionally, some ion conductors such as Li_6PS_5Cl feature flat energy landscapes with a lack of well-defined energy minima that make it difficult to well define Li-ion sites. In this section we set out to investigate how *well-defined* sites are in disordered-Li_9S_3N and to what extent Li jumps are correlated in disordered-Li_9S_3N and ordered-Li_9S_3N.

Particle motion may be described in terms of hopping when diffusion through the solid occurs as a series of distinct events. One may test for this by verifying whether the conditions listed in Table S11 are met for the material of interest. We find the conditions to describe diffusion in terms of hopping met for disordered-Li_9S_3N (Table S12).

Table S11. Requirements to describe diffusion processes as "hops" adapted from [8]

$\tau_r >> \tau_h$	The hopping time, τ_h, associated with the event when compared with the residence time, τ_r, spent by a given particle between hops (i.e. in sites).
$\tau_r >> \nu_0^{-1}$	For ν_0 the mean thermal vibrational frequency, i.e. the attempt frequency
d>>a	For d the hopping distance (i.e. distance between sites) and a the mean amplitude of thermal motions
Ea>>kT	For E_a the maximum change in potential energy during a hopping even

Table S12. Verifying the conditions for describing ion diffusion as hopping in disordered Li_9S_3N. τ_r residence time, τ_h, the time during the hop, ν_0 attempt frequency (≈10^{13} Hz), a, vibration amplitude, d distance between sites, Ea/kT assuming Ea = 0.2 eV which is on the order of the lowest jump-activation energies* in $Li_{2+x}S_{1-x}N_x$ phases.

T_r/T_h	T_r/ν_0^{-1}	d/a	Ea/kT (T=900K)	Ea/kT (T=300K)	Condition met
5.35	1684.98	5.26	2.67	8	yes

* Catlow explicitly refers to ΔE as the change in potential energy for this criterion, accessible by static techniques. We do not have access to the *static* potential energy per se based on our current data but we believe the jump-Ea values provide a reasonable estimation, averaged in space and time as discussed in *Supplementary Note 5*.

In addition to the above criteria Catlow et al (ref.[8]) described 2 exact criteria to validate whether the hopping model applies:

The 1st criterion requires a highly structured pair correlation function of the mobile species. The 2nd criterion requires a non-gaussian self-correlation function of the mobile species. Figure S18a and b show the pair correlation functions and the self-correlation of the Li ions. The pair Li-Li correlation function is highly structured and the self-correlation functions is non-Gaussian as may be quantitatively determined from evaluating $3^*\langle r^4(t)\rangle/5^*\langle r^2(t)\rangle$ which is of the order of 30 (unity would indicate liquid-like diffusion). $\langle r^n(t)\rangle$ is defined in equation S3 as :

$$< r^n(t) > = \int dr\, r^n\, G^S(r,t) \qquad (S3).$$

Additionally, the clearly visible gap in Li-population visible in Figure b between ~0 Å and ~3 Å shows that Li ions favorably reside in their initial site (0 Å) or in a neighboring site (3 Å) which is approximately the distance between octahedral and tetrahedral sites. At higher radii such clear gaps are not visible as jumps do not occur in a straight line i.e. the distance from the second site to the initial site is not necessarily twice the distance from the first site to the initial site.

We now concern ourselves with the extent to which motion is *correlated* in disordered-Li_9S_3N. Previously correlated motion has been detected by two means: (i) by demonstrating *bundling in time* of jump events and (ii) by identifying a distribution of *strings of jumps* that does not follow a Poisson distribution. A string consists of at least two jumps where the second jump departs from the end-site of the first jump. The start-time of this second jump needs to be after the start time of the first jump and before the end-time of the first jump. Generally a string of length n is given if all the below conditions are met where i represents jump-event indices of jumps that are part of the string; $t_{i,start}$ and $t_{i,end}$ relate to the time in the simulation where event i starts and ends, respectively. $Site_{i,start}$ / $Site_{i,end}$ stand for start-site and end-site of jump event i, respectively:

$$\mathrm{t_{i-1,start}} \leq \mathrm{t_{i,start}} \cap \mathrm{site_{i-1,end}} = \mathrm{site_{i,start}} \cap \mathrm{t_{i-1,end}} \geq \mathrm{t_{i,start}} \text{ for i in } [2,3,4,5 \ldots, \mathrm{n}] \qquad (S4)$$

In Figure S18c we show that no bundling in time of the hop events as observed by Mo et al.[7] and Morgan[6] is observed for AIMD simulations of the $Li_{2+x}S_{1-x}N_x$ phases suggesting that the mechanism described by Mo et al. [7] is not dominant in disordered-Li_9S_3N phases.

To analyse the *poissonicity* of the probability distribution of string lengths for each AIMD simulation we performed the following analyses.

1) We compared the probability distribution of string lengths obtained in one AIMD simulation and the probability distribution of an *ideal* Poisson distribution with λ=average-string-length of the AIMD simulation (*ideal* meaning a Poisson distribution with a very large distribution size, $>10^6$ samples). Figure S18d shows that the distribution of string lengths follows a Poisson distribution.

2) While the distribution of string lengths closely follows a Poisson distribution deviations can still be made out. Such deviations may represent a deviation from Poisson behaviour or be a consequence of a limited size of distributions. In order to quantify the magnitude of the deviations

caused by limited distribution size we calculated the Cramer-von-Mises statistic for the distribution of string lengths in different AIMD simulations vis-à-vis an *ideal* Poisson distribution. Additionally we calculated the Cramer-von-Mises statistic for 100 control Poisson distribution with λ=average-string-length, each of these control Poisson distributions had the same distribution-size as the distribution of string lengths obtained from the AIMD simulations. We find that the deviations from an *ideal* Poisson distributions that we observe can be accounted for by the limited AIMD-string-length distribution's size because similar Cramer-von-Mises statistics are obtained for the string-length distributions obtained from AIMD and the 100 control Poisson distributions of equivalent size vis-à-vis an *ideal* Poisson distribution with distribution size $>10^6$ (Table S10).

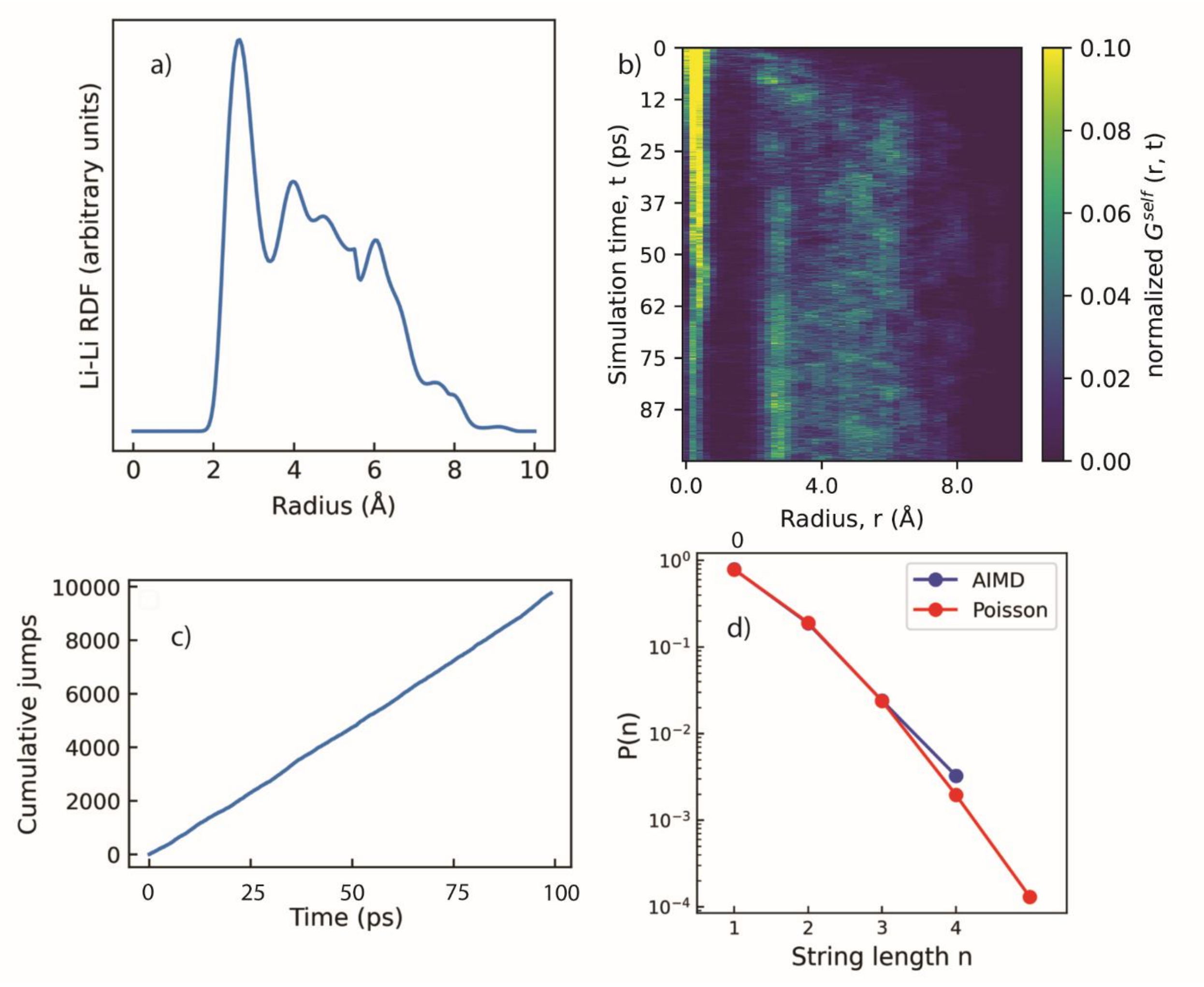

Figure S18. Validity of the hopping model and the correlation of hops in disordered-Li_9S_3N. (a) Li-Li RDF of the AIMD of disordered Li_9S_3N at 900K. The RDF is *well-structured* suggesting well-defined sites. (b) Self correlation function of the Li-ions in the AIMD. (c) Cumulative jumps over the AIMD simulation time. No *cascades* of jumps are observable the number of jumps increases continuously. (d) Distribution of the string lengths together with an *ideal* Poisson distribution with λ = average-string-length from AIMD.

In conclusion, the diffusion in disordered-Li_9S_3N clearly occurs via hops between well-defined sites and in an uncorrelated fashion.

The same investigation of the Li-diffusion was performed for ordered-Li_9S_3N and we equally find that diffusion in ordered Li_9S_3N occurs via hops between well-defined sites in an uncorrelated fashion Figure S18.

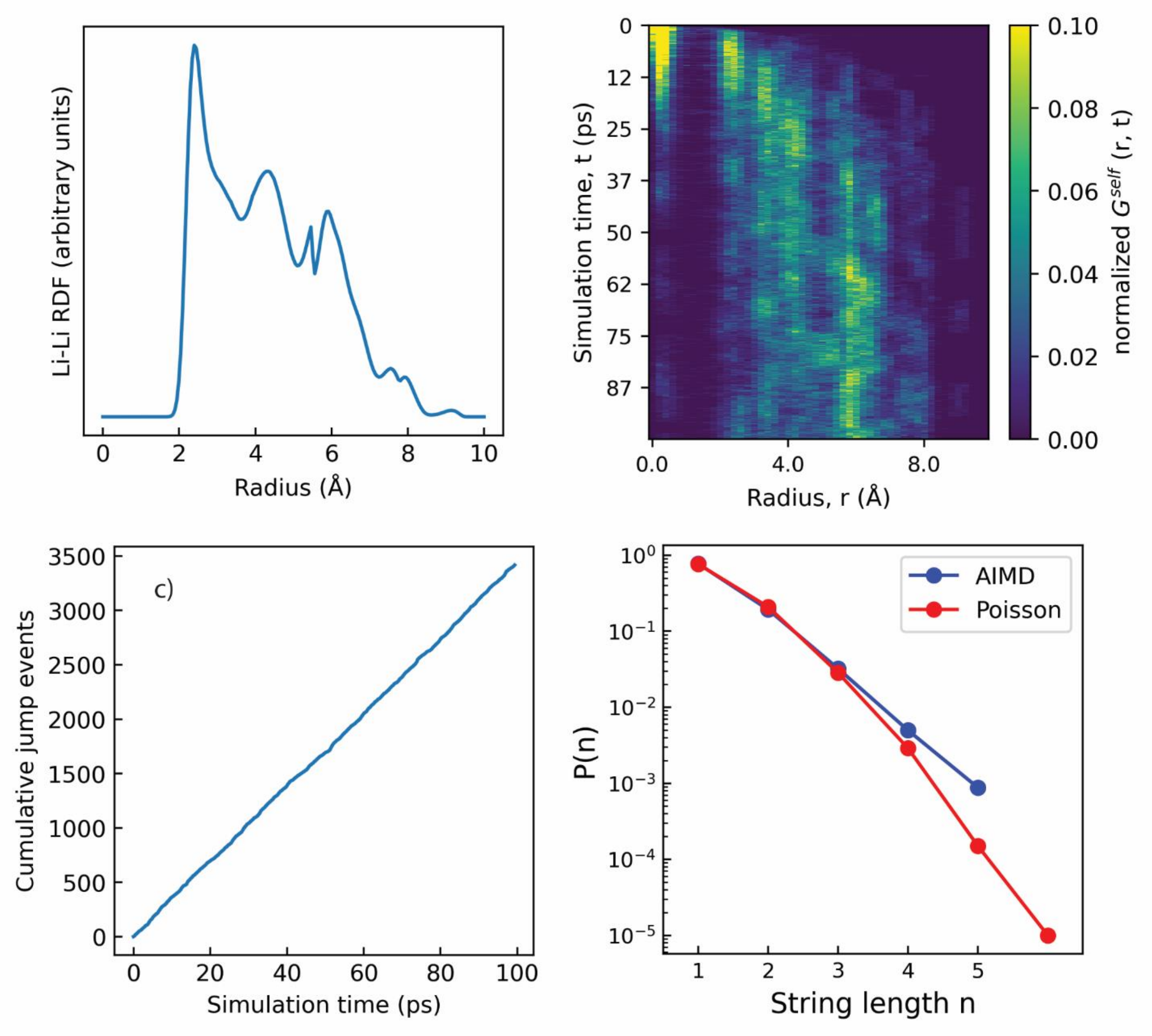

Figure S19. This Figure addresses the validity of the hopping model and the correlation of hops in ordered-Li_9S_3N. (a) Li-Li RDF of the AIMD of ordered Li_9S_3N at 900K. The RDF is *well-structured* suggesting well-defined sites. (b) Self correlation function of the Li-ions in the AIMD. (c) Cumulative jumps over the AIMD simulation time. No *cascades* of jumps are observable the number of jumps increases continuously. (d) Distribution of the string lengths together with an *ideal* Poisson distribution with λ = average-string-length from AIMD.

Table S13: Results of Cramér-von Mises analysis for the data in Figures S18d and S19d

Phase	Cramér-von Mises statistic for string length distribution obtained from AIMD simulations	Average Cramér-von Mises statistic of 100 Poisson distributions of equivalent length as the string length distribution obtained from AIMD
Disordered-Li_9S_3N	946.245	946 ± 1
Ordered-Li_9S_3N	508.001	509 ± 1

Supporting Note 4: Estimation of the *attempt* frequency v_0

In the field of solid-state ionics it is commonly accepted that 10^{13} Hz is a reasonable *a priori* assumption for the attempt frequency v_0 as explored e.g. in references [9–12].

We support our assumption of $v_0 = 10^{13}$ Hz for the $Li_{2+x}S_{1-x}N_x$ phases by computationally estimating v_0 using a previously published approach [13–15] briefly described as follows: The oscillations of the Li-ions around the site centers are obtained from AIMD simulations. A Fourier transform of this oscillatory signal in time yields a distribution of the frequencies of the underlying oscillations. The average oscillatory frequency serves as an estimate for the attempt frequency v_0. The average oscillatory frequencies for different $Li_{2+x}S_{1-x}N_x$ phases obtained in this way are shown in Table S14. Clearly the estimates for the attempt frequency v_0 obtained from this method are on the order of $1*10^{13}$ s^{-1}.

Table S14. Average frequency of oscillatory motion in Li sites obtained from 900K AIMD simulations of different $Li_{2+x}S_{1-x}N_x$ supercells.

Supercell composition x in $Li_{2+x}S_{1-x}N_x$	Average frequency of oscillatory motion in Li sites (10^{13} Hz). Error bar is the standard deviation
0.25 (i.e. disordered-Li_9S_3N)	1.09 ± 0.04
0.34	1.09 ± 0.05
0.44	1.11± 0.03
0.5	1.12± 0.02

Additionally, Equation S5 derived from transition theory allows to estimate the average attempt frequency from the experimental activation energy as done in ref. [16]:

$$v_0 = \frac{1}{a_0}\sqrt{\frac{2\,E_a}{M_{Li}}} \qquad (S5)$$

where v_0 is the attempt frequency α_0 the average (jump) distance between sites, E_a the activation energy for a jump between sites and M_{Li} the mass of a Li-ion. This approach is approximate as Equation S5 is derived for a parabolic potential-well (ref. [17]**)** but serves to estimate the order of magnitude of the average attempt frequency. Table S15 shows the attempt frequencies calculated in this way for different $Li_{2+x}S_{1-x}N_x$ phases. (2.5 Å was used as an average distance between jumps i.e. average of the 2.7 Å tet-tet distance and the average oct-tet 2.3 Å distance.) This second approach based on experimental conductivity-activation energies also yields estimates for $v_0 \approx 1*10^{13}$ Hz

Table S15. Attempt frequency obtained by applying Equation S5 to different $Li_{2+x}S_{1-x}N_x$ phases using the experimental conductivity-Ea.

Composition, x in $Li_{2+x}S_{1-x}N_x$	Experimental Ea (eV)	Attempt frequency v_0/ 10^{13} Hz
0.05	0.47 ± 0.01	1.02 ± 0.011
0.1	0.45 ± 0.01	1.00 ± 0.010
0.2	0.40 ± 0.01	0.94 ± 0.012
0.25	0.39 ± 0.01	0.93 ± 0.012
0.45	0.38 ± 0.01	0.91 ± 0.012

The two independent approaches to estimate the order of the attempt frequencies yield values of the order of 10^{13} Hz justifying the order of magnitude of the assumed $v_0 = 10^{13}$ Hz .

Small deviations of the attempt frequency about this order of magnitude would cause a small systematic shift of the individual jump-Ea values and so of the percolation-energy curves. We demonstrate this in SI Figure S20 by recalculating the percolation-energy diagram for three values of $0.9 \cdot 10^{13}$ Hz ≤ v_0 ≤ $1.1 \cdot 10^{13}$ Hz resulting in deviations of < 0.01 eV. The shape of the percolation diagram is not affected and so we conclude that the exact value of v_0 would not affect any of the conclusions in our study.

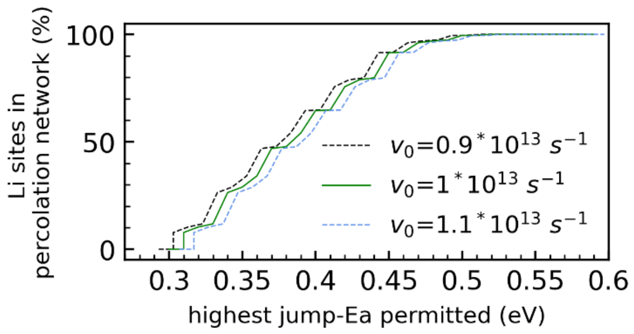

Figure S20. Percolation energy diagram for disordered-Li_9S_3N with jump-Ea values calculated with $v_0 = 0.9 \cdot 10^{13}$ Hz , $v_0 = 1 \cdot 10^{13}$ Hz, $v_0 = 1.1 \cdot 10^{13}$ Hz

To the best of our knowledge, it is typically assumed that the attempt frequency is a material property, constant in all sites. [9–12] It is however, conceivable that the difference in coordination environment in disordered materials and/or materials with multiple distinct sites effects a significant difference in effective attempt frequency per site in a given material.Finally, to investigate the effect on our conclusions of hypothetical variations of the attempt frequency in different sites in $Li_{2+x}S_{1-x}N_x$ we recalculate the jump-Ea values using v_0 with a 30% uncertainty that is $(1\pm0.3)*10^{13}$ Hz instead of $v_0 = 1*10^{13}$ Hz. The corresponding uncertainty on the jump-Ea values amounts to 40-50 meV (rather than 10-20 meV). Key figures of the main text are reproduced in Figure S21 below with the increased uncertainty in the attempt frequency. Small shifts of the percolation onsets are observed and the distribution of possible fraction of sites in percolation network is increased as would be expected. Overall, however, none of the conclusions of our study are affected.

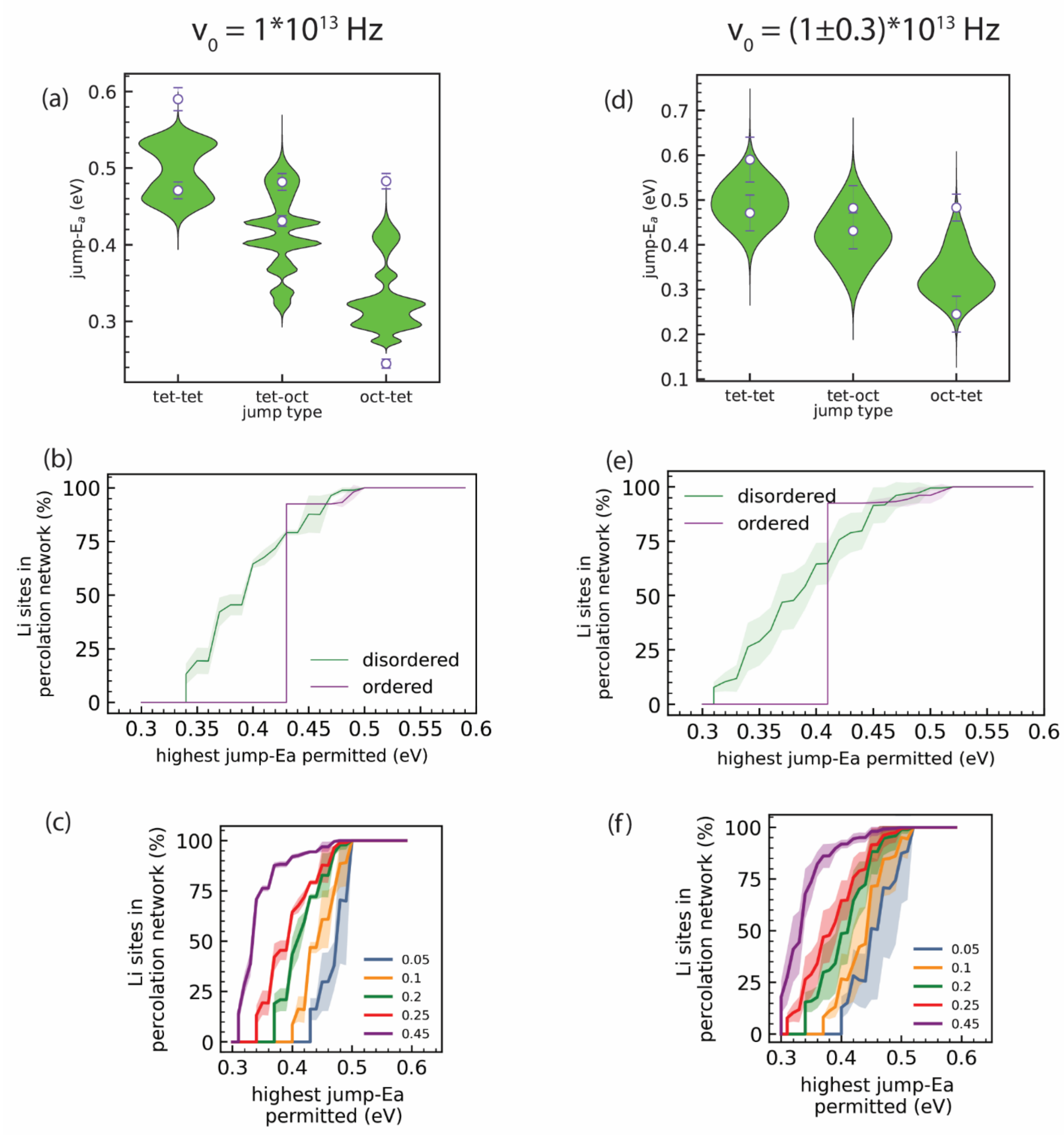

Figure S21. Key figures from the main text reproduced with $v_0=(1±0.3)\cdot10^{13}$ Hz to account for hypothetical 30% variations in the attempt frequency between sites, resulting in larger uncertainty on the jump-Ea (a) Figure 4a from main text. (b) Figure 4b from main text. (c) Figure 7b from main text

Supporting Note 5: Conceptual differences between the jump activation energies from AIMD and the energy barriers obtained from NEB

Migration barriers obtained by the Nudged-elastic band approach (NEB) require an a priori knowledge or intuition of the jump event. The researcher will have to make a choice on whether the ion to be investigated takes part in an independent jump or a correlated jump and, in the latter case, how many ions will be involved. Structural disorder —whether compositional, occupational, displacive, rotational etc.— needs to be defined explicitly. For example, in the case of $Li_{2+x}S_{1-x}N_x$, the local coordination by S/N and the occupation or not of adjacent tetrahedral and octahedral sites (i.e. *defect*/*charge-carrier* concentration and distribution) needs to be explicitly defined. Finally, by the nature of the NEB methodology, the quantitative barrier can only be determined statically (i.e. at 0 K), that is any contribution from any thermally activated dynamics is normally excluded.

In contrast, jump-activation energies are obtained from AIMD simulations at finite temperatures by determining the jump frequency $\nu_{A\text{->}B}$ for jumps going from site A to site B (count of jumps divided by the time of site occupation of site A). Provided that the jump frequency exhibits Arrhenius behavior and that the attempt frequency ν_0 is known (or an appropriate estimate used), a jump activation energy can be calculated as shown in equation (1) from the main text:

$$jump\text{–}\, E_{a,A\rightarrow B} = -\mathrm{k_b T} * \ln\left(\frac{\nu_{\mathrm{A\rightarrow B}}}{\nu_0}\right) \quad (1)$$

where k_b is the Boltzmann constant, T the temperature in K, $\nu_{A\text{->}B}$ the observed frequency of jumps between sites A and B and jump-$E_{a,\, A\text{->}B}$ the jump-activation energy of a jump event from site A to site B. Across the simulation time different jumps between the sites of interest occur, certain jump events may be part of correlated “strings” and other jump-events may be “independent”. Additionally these jump-events occur with different arrangements of the surrounding mobile species which inherently changes during the AIMD simulations. In contrast to NEB migration barriers, jump-Ea values from AIMD can thus be interpreted as *time-averaged* migration barriers associated with all occurring types of jump events (i.e. independent and correlated) and associated with a *time-averaged* occupation of the surrounding mobile-species sites rather than a specific arrangement of the mobile species. In case no appropriate ν_0 value can be identified and/or jumps between sites do not exhibit Arrhenius behavior, the jump-Ea values can still be interpreted as rescaled jump-frequencies which are still highly informative and enable to identify diffusion-promoting and diffusion-hampering local environments.

Supporting Note 6: Uncertainty on jump-Ea values

Jump-Ea values are calculated between a pair of sites (i.e. two neighbouring sites) A and B via equation 1 of the main text:

$$jump\text{-}\,E_{a,A\rightarrow B} = -\mathrm{k_b T} * \ln\left(\frac{\nu_{\mathrm{A\rightarrow B}}}{\nu_0}\right)$$

where k_b is the Boltzmann constant, T the temperature in K, $\nu_{A\text{->}B,}$the observed frequency of jumps between sites A and B i and jump-$E_{a,\ A\text{->}B}$ the average jump-activation energy of a jump event from site A to site B. Jump types are defined based on the composition of site A and site B, as well as the bottleneck connecting the two sites. Specific jump types, for instance N_3S_1-N_3S_3(NNN), are represented by numerous pairs of sites through the multiple supercells simulated. The average jump-Ea of a jump type is the arithmetic mean of the jump-Ea values of all pairs of sites representing that jump type and its standard deviation as the uncertainty on the mean (ε_{mean}). An additional uncertainty on the average jump-Ea values arises from convergence. To estimate the uncertainty arising from convergence we evaluate the average jump-Ea value over different stretches of the AIMD simulation time i.e. from 20 ps to 200 ps in steps of 20 ps as demonstrated for the example of N_3S_1-N_3S_3(NNN) jump-type in SI Figure 22. The uncertainty on the average-jump-Ea arising from convergence ($\varepsilon_{convergence}$) is estimated as the largest difference between jump-Ea values existing between 140 ps and 200 ps simulation time:

$$\epsilon_{convergence} = \max\left(|E_{200ps} - E_{180ps}|, |E_{200ps} - E_{160ps}|, |E_{200ps} - E_{140ps}|, |E_{180ps} - E_{160ps}|, |E_{180ps} - E_{140ps}|, |E_{160ps} - E_{140ps}|\right)$$

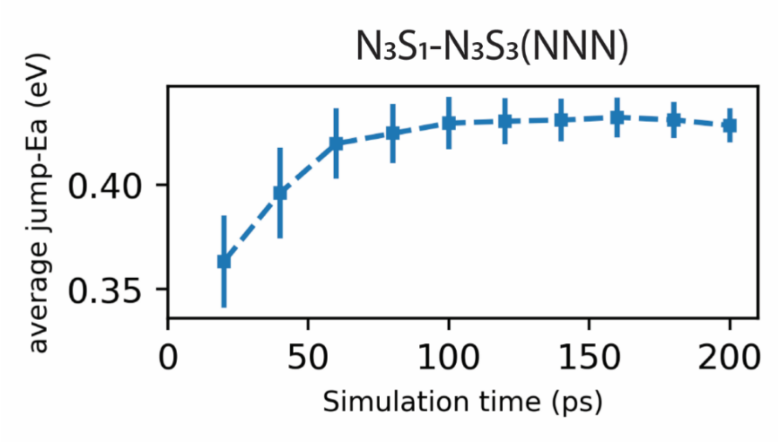

Figure S22. Average jump-Ea of the N_3S_1-N_3S_3(NNN) jump evaluated for different AIMD simulation times.

The uncertainty on the average jump-Ea value for a jump type $\varepsilon_{jump\text{-}Ea}$ is defined as:

$$\epsilon_{jump-Ea} = \epsilon_{convergence} + \epsilon_{mean}$$

and is typically of the order of 10-20 meV. The $\varepsilon_{jump\text{-}Ea}$ and its components ε_{mean} and $\varepsilon_{convergence}$ are tabulated for every jump type SI Table S5. The exact same procedure was applied to extract the jump-Ea values for ordered-Li_9S_3N from an AIMD simulation of an ordered-Li_9S_3N supercell (SI Table S6).

Supporting Note 7: Jump-Ea values in ordered- versus disordered-Li_9S_3N

Differences in the jump activation energies for the same jump types in ordered and disordered Li_9S_3N suggest that the long-range anion-ordering in ordered-Li_9S_3N has an effect on the jump activation energy values in addition to the local N/S occupation of polyhedra and bottlenecks.

Table S16. Comparison of the jump-Ea values obtained for the jump types observable in ordered-Li_9S_3N from AIMD on ordered-Li_9S_3N and disordered-$Li_{2+x}S_{1-x}N_x$ supercells.

	ordered		disordered	
Jump type	Average jump-Ea (eV)	$\varepsilon_{jump\text{-}Ea}$ (eV)	Average jump-Ea (eV)	$\varepsilon_{jump\text{-}Ea}$ (eV)
N_1S_3- N_2S_4 (NSS)	0.431	0.005	0.402	0.004
N_2S_4- N_1S_3 (NSS)	0.245	0.004	0.297	0.008
N_1S_3- S_6(SSS)	0.482	0.011	0.414	0.014
S_6- N_1S_3(SSS)	0.483	0.010	0.409	0.011
N_1S_3-N_1S_3(SS)	0.59*	0.015	0.488	0.01
N_1S_3-N_1S_3(NS)	0.472	0.011	0.477	0.011

Jump-Ea values depend on the occupancy of the 'start' and 'end' sites. Ordered-Li_9S_3N features 3 Li sites S_6, N_1S_3, N_2S_4. The former two sites are highly occupied, the latter N_2S_4 site is highest in energy and essentially unoccupied. The effect of long-range anion ordering on jump-Ea values will be discussed on the example of S_6 - N_1S_3(SSS) and N_1S_3-S_6(SSS) jumps.

The jump frequency for S_6 - N_1S_3(SSS) jumps depends on the low probability of N_1S_3 sites being vacant. In ordered Li_9S_3N vacant N_1S_3 sites may only be obtained by the unfavourable occupation of N_2S_4 sites[†] and this effect increases the jump-Ea values of S_6 - N_1S_3(SSS) jumps —and by the same token increases jump-Ea values of N_1S_3-S_6(SSS) jumps since vacancies on S_6 may also only be obtained by N_2S_4 site occupation. In contrast in disordered Li_9S_3N supercells N_1S_3 sites are more likely to be vacant due to the presence of lower-energy octahedra that are more favourably occupied than N_2S_4 sites. This increased likelihood of a vacancy on a N_1S_3 site is reflected in higher S_6 - N_1S_3(SSS) jump-frequencies and thus lower jump-Ea values.

The diffusion-hampering effect of long-range anion-ordering on jump-Ea values may be observed by performing percolation analysis for ordered-Li_9S_3N with the jump-Ea values from disordered-Li_9S_3N. In Figure S23 the results from the main text (where the effect of long-range anion ordering is taken into account) are compared with the same analysis but applying the jump-Ea values from disordered-Li_9S_3N to ordered-Li_9S_3N. As shown in Figure S23d the onset is shifted up by 0.03 eV when considering the effect of long-range anion-ordering on jump-Ea values highlighting a Li-diffusion hampering effect of the anion-ordering in ordered-Li_9S_3N.

[†] That is, essentially an energetically costly Frenkel-defect formation.

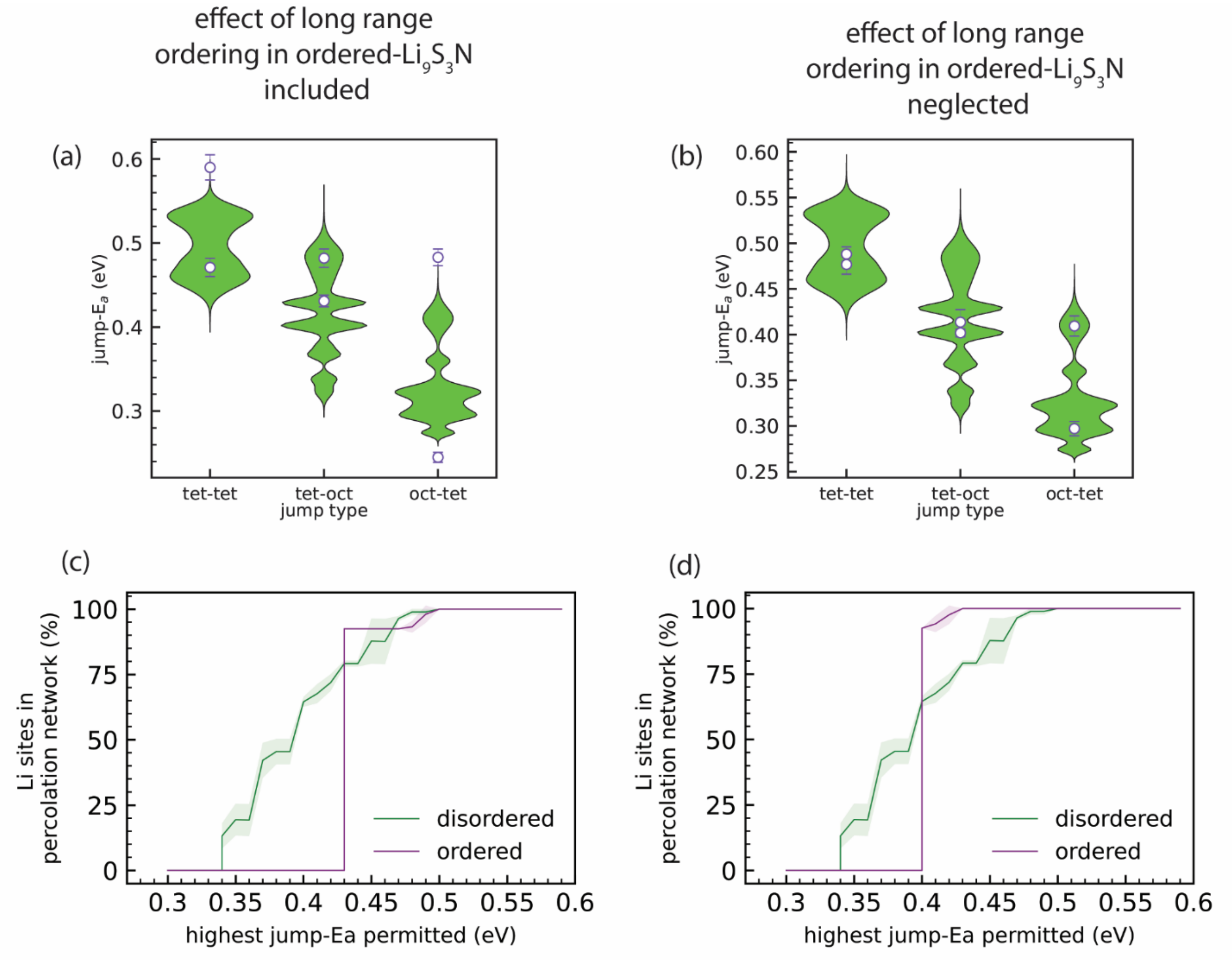

Figure S23. Effect of different jump-types existing in ordered and disordered Li_9S_3N. (a) and (b) Comparison of the observable jump-activation energies in ordered and disordered-Li_9S_3N. Purple markers indicate jump-Ea of six distinct jump types possible in ordered-Li_9S_3N, labelled with the *start-end(bottleneck)* notation explained in the main text. In disordered-Li_9S_3N 91 different jumps are observable, listed in SI Table S5 and shown here as density plots (*violins*). The horizontal scale of the violins represent the relative occurrence of jump types at that energy. In (b) the jump-Ea values obtained for disordered-Li_9S_3N were used. (c) and (d) Percolation-energy diagram showing the fraction of Li sites that are connected to percolating networks (averaged over 50 disordered 5x5x5 supercells) for ordered- and disordered-Li_9S_3N. Shading is the standard deviation over 50 supercells. The vertical lines indicate the experimental activation energy $E_{a,EIS}$ obtained experimentally from EIS for ordered- and disordered-Li_9S_3N (see Figure 2). In (d) the jump-Ea values obtained for disordered-Li_9S_3N were used.

Supporting Note 8: Additional details on the interpretation of percolation-energy diagrams

Energy-percolation diagrams enable to estimate a range within which the bulk-activation energy of a solid-electrolyte will lie provided two conditions are given:

(1) The jump frequency between sites A and B exhibits Arrhenius behaviour to ensure validity of equation 1 from the main text

(2) An accurate estimate of the v_0 value is employed in equation 1 from the main text, and it should be estimated to what extent v_0 varies across different sites in the phase.

If the above conditions are fulfilled the range of the bulk activation energy may be obtained as follows:

The experimental activation energy will be at the percolation-onset or above since the percolation onset presents the minimum energy threshold to be overcome for percolation. So far we can thus say that energy-percolation diagrams predict that the bulk activation energy will lie in the range [percolation-onset, ∞]. But the upper limit for the predicted bulk activation energy may potentially be further reined in as follows:

Diffusion through sites connected by 0.55 eV is negligible compared to diffusion through sites connected by 0.35 eV as diffusion through the latter is >10^3 times faster. However, diffusion through sites connected by jump-activation energies of 0.35 eV is only twice as fast as through sites connected by 0.36 eV. In case a percolation onset was at 0.35 eV, diffusion through sites connected by 0.36 eV would not be negligible and may contribute to the overall diffusion which in turn may increase the overall activation energy (i.e. the experimental activation energy) beyond the percolation onset. Accordingly the experimental activation energy may be above the percolation onset. However, contribution of sites diffusing 1 order of magnitude slower than the sites at the percolation-onset is likely negligible. At 300 K it takes an activation energy increase of 0.06 eV before the jump-frequency reduces by a factor 10 (this is independent of the attempt frequency v_0 used, see Figure S24). Based on this analysis we propose that the experimental activation energy will likely be in the range [percolation-onset, percolation-onset+0.06 eV]. **We highlight that this is only rough approach to obtain an estimate of the upper limit**. However, the experimental activation energies obtained with EIS for different $Li_{2+x}S_{1-x}N_x$ phases fall well into the ranges that may be gauged from percolation-energy diagrams in this way:

Table S17. Predicted range of activation energy by percolation-energy analysis. The experimentally measured activation energies from EIS are in the said ranges.

x in $Li_{2+x}S_{1-x}N_x$	Experimental activation-energy-range predicted from percolation-energy diagrams (eV)	Measured activation energy (eV)
0.05	[0.43, 0.49]	0.47 ± 0.01
0.1	[0.40, 0.46]	0.45 ± 0.01
0.2	[0.37, 0.43]	0.40 ± 0.01
0.25	[0.34, 0.4]	0.39 ± 0.01
0.45	[0.31, 0.37]	0.38 ± 0.01

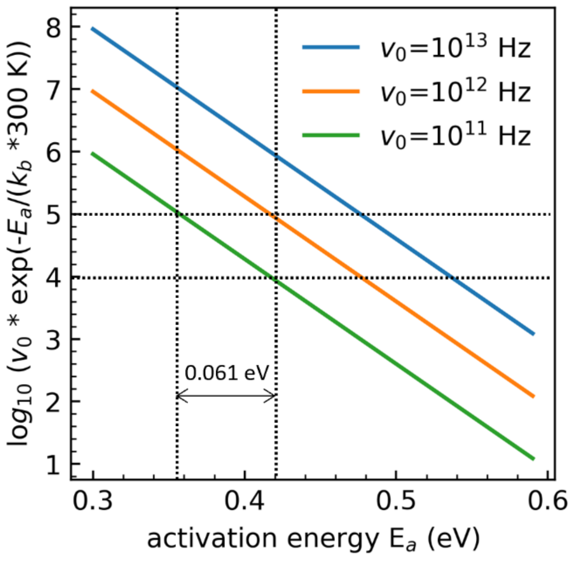

Figure S24. Evaluation of jump frequencies at different activation energies for different attempt frequencies v_0. The point of this figure is to illustrate that the jump-frequency drops by a factor 10 if the activation energy is increased by 0.061 independent of the choice of the attempt frequency.

Supporting Note 9: Application of MD-percolation methodology on Li_6PS_5Br

To establish the wide applicability of the methodology developed to analyse disordered solid electrolytes, we investigate the Li_6PS_5Br argyrodite system. Li_6PS_5Br crystallizes in the cubic $F\bar{4}3m$ space group (216), as depicted in Figure S25. In this structure, lithium ions typically occupy the T5 (Wyckoff 48h), T2 (Wyckoff 48h), and T4 (Wyckoff 16e) ref([18–23]) sites. Here, for the sake of simplicity, only the T5 (Wyckoff 48h) and T4 (Wyckoff 16e) sites were considered.

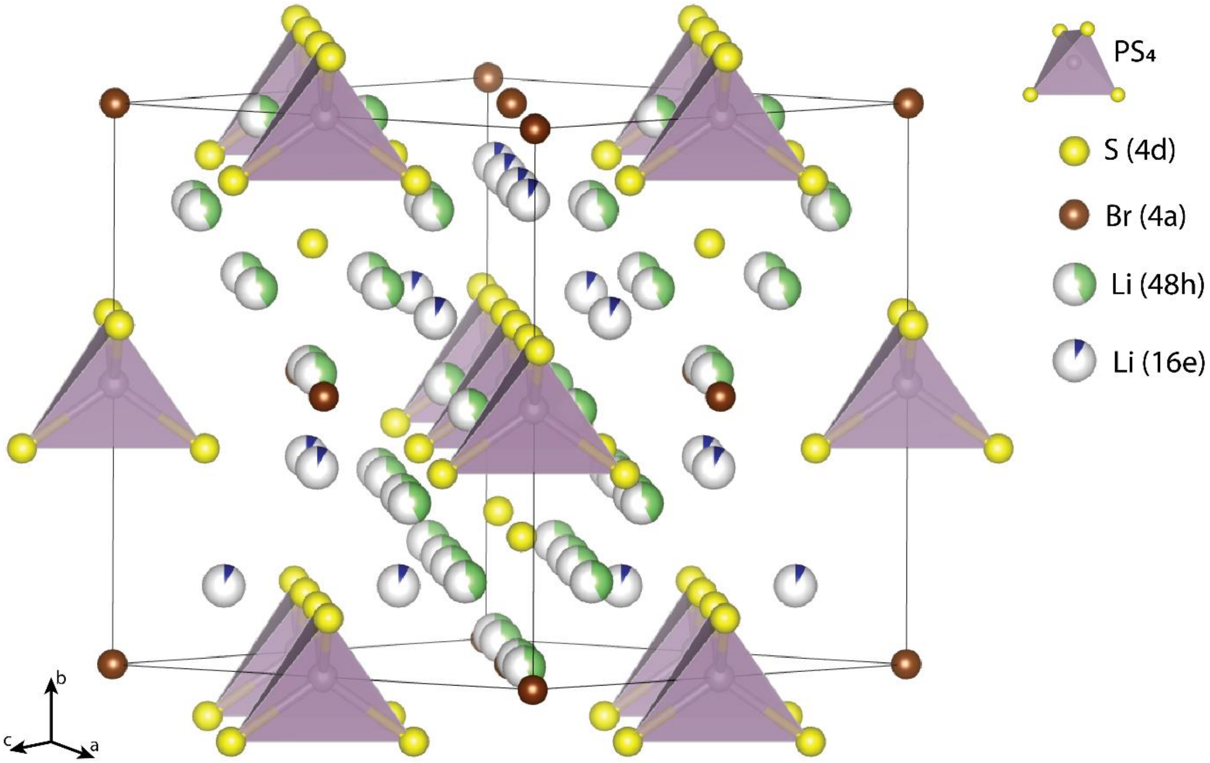

Figure S25. Ordered crystal structure of Li_6PS_5Br (0% S/Br-site inversion), where Br^- ions are located at the Wyckoff 4a positions and S^{2-} ions (not bonded to P^{5+}) occupy the Wyckoff 4d positions. The anion framework forms 136 interstitial tetrahedral voids per unit cell, suitable for cation occupancy. Four of these voids are filled by P^{5+} cations at the Wyckoff 4b site, forming PS_4^{3-} tetrahedra. The remaining 132 tetrahedral voids can accommodate lithium[9]. Lithium sites Wyckoff 48h and Wyckoff 16e, which are considered in the current study, are presented.

Lithium ions distributed across the Wyckoff 48h positions form a cage-like substructure encircling the 4d site, while the Wyckoff 16e positions are located between these cages (Figure S25). This configuration defines three types of Li-ion movements: doublet, intracage, and intercage jumps.[21] Doublet jumps occur between paired 48h sites (distance 1.9 Å), and intracage jumps occur between pairs of 48h sites (distance 2.25 Å) within a cage. The intercage jumps, which are essential for long-range lithium-ion diffusion, occur between the cages via the 48h–16e–48h pathways.

The coordination of 48h and 16e tetrahedra for each jump type is depicted in Figure S26a. The 48h tetrahedra (in green) are formed by two S^{2-} ions (corner-shared with PS_4 tetrahedra in violet) and two anions at the 4a and 4d sites, respectively. 16e sites (in blue) are coordinated by three S^{2-} ions (also corner-shared with PS_4 tetrahedra in violet) and one anion at the 4a site. Due to the similar ionic radii of bromine and sulfur ions, they can exchange positions and occupy both 4a and 4d sites (Figure S26a). This introduces site disorder in the structure and creates different local environments that affect lithium mobility.

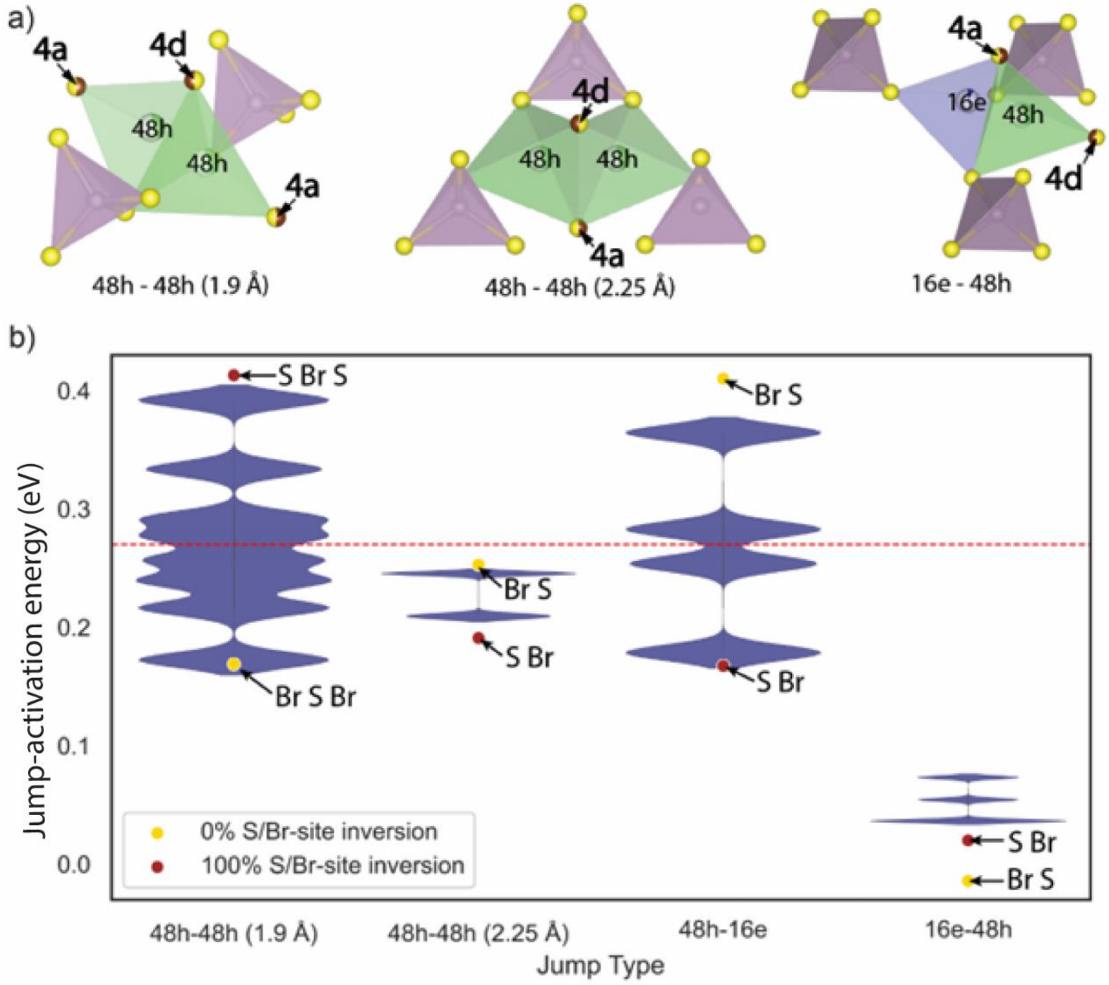

Figure S26. Impact of sulfur/bromine local environments on jump activation energy. a) Illustration of the coordination of 48h and 16e tetrahedra for each jump type, where the 4a and 4d sites used for jump environment nomenclature are shown as partially occupied by sulfur and bromine (half-yellow, half-red dots) and highlighted by arrows. b) Comparison of jump activation energies per possible jump environment within Li_6PS_5Br composition, calculated from AIMD at 900 K. Yellow and red solid dot markers indicate the jump activation energies for Li_6PS_5Br (0% S/Br-site inversion) and Li_6PS_5Br (100% S/Br-site inversion), respectively. Jump local environments are labelled with the “4a 4d 4a” notation for 48h–48h (1.9 Å) doublet jumps and “4a 4d” notation for48h–48h (2.25 Å) intracage, 48h–16e, and 16e–48h jumps, as explained in the main text. The average jump activation energy, calculated across eight Li_6PS_5Br 2x1x1 supercells with different site disorder (Table S18), is shown in blue as “violins”. The width of the violins represents the relative occurrence of each jump local environment at that energy in Li_6PS_5Br (50% S/Br-site inversion) structure.

In analogy to the study of $Li_{2+x}S_{1-x}N_x$, we calculate jump-Ea from the jump-frequency $\nu_{A\text{->}B}$ between sites, segregated by local environment:

$$jump\ E_{a,A\rightarrow B} = -\mathrm{k_b T ln}\left(\frac{\nu_{\mathrm{A\rightarrow B}}}{\nu_0}\right) \quad (S6)$$

where k_b is the Boltzmann constant, T the temperature in K, $\nu_{A\text{->}B}$ the observed frequency of jumps between sites A and B, jump-$E_{a,\ A\text{->}B}$ the jump-activation energy[‡] of a jump event from site A to site B and ν_0 the attempt frequency.

We investigated the impact of the local jump environment created by the distribution of bromine and sulfur across the 4a and 4d sites on the Li-ion transport and jump activation energy by examining AIMD simulations of eight configurations of the Li_6PS_5Br composition, each having different S and Br distributions on these sublattices (Table S18). The structure set was chosen to ensure multiple repetitions of each possible anionic environment and to obtain reliable statistics. The average activation energy for each type of jump across the different local environments is depicted in Figure S26b. To characterize a local jump environment, we label the anions occupying the 4a and 4d positions of the start site and the 4a position of the end site in 48h–48h (1.9 Å) doublet jumps, as both 48h sites share the same 4d position but differ in 4a positions (Figure S26a). For 48h–48h (2.25 Å) intracage, 48h–16e, and 16e–48h jumps, the labels include the anions occupying the 4a and 4d positions since both the start and the end sites share the same environment (Figure S26a).

In the ordered configurations of Li_6PS_5Br (0% and 100% S/Br-site inversion), distinct jump activation energies were observed, with only one possible jump local environment per jump type. Similar to the ordered-Li_9S_3N structure, both ordered configurations of Li_6PS_5Br do not exhibit low-energy percolating paths[§]. To illustrate this, we take the example of a jump activation energy of 0.3 eV (Figure S26b). Li_6PS_5Br with 0% S/Br-site inversion does not feature 48h-16e jump with activation energy < 0.3 eV, while Li_6PS_5Br

---

[‡] Note: Care needs to be taken on how to interpret the jump-Ea. If the jump frequency exhibits Arrhenius behavior and if the exact attempt frequency is known then the jump-Ea represents the 'time-averaged' energy-barrier for the transitions between sites A and B. If the jump frequencies do not follow an Arrhenius law and/or a good estimate of ν_0 cannot be obtained then the jump-Ea is "merely" an arbitrary rescaling of the jump-frequency. In such cases jump-Ea values are not time-average energy barriers but still allow to identify diffusion-promoting and diffusion-hampering local environments. In both cases, percolation-energy diagrams may be calculated. In both cases percolation onsets at lower energies indicate the possibility of a phase to feature faster Li-diffusion typically correlated with higher Li-conductivities. In Li_6PS_5Br, the jump-frequencies in Li-argyrodites are likely governed by an Arrhenius law since the experimental bulk conductivity (which consists of individual Li-transitions/"jumps") follows an Arrhenius law. [24] For the present example we will assume $\nu_0 = 1*10^{13}$ Hz in all sites and will not further investigate possibly existing variations of ν_0 in different sites. We will interpret the jump-Ea values in this study as "rescaled jump-frequencies" which enable to identify diffusion-promoting and diffusion-hampering local environments and are thus not concerned with the exact value of ν_0. Nonetheless we would like to highlight that the choice of $\nu_0 = 1*10^{13}$ Hz is not unreasonable and may be a good approximation: The attempt frequency for a material may be estimated from the oscillations of the Li-ions and for the 8 Li_6PS_5Br supercells on which we performed AIMD simulations ν_0 values of 8.67, 8.63, 8.4, 8.23, 8.39, 8.29, 8.39 and 7.96 GHz were obtained.

[§] *Note:* **Figure S21** contains negative jump-Ea values which may at first be perplexing but we would like to highlight again that for this study we interpret the jump-Ea "merely" as rescaled jump-frequencies. A negative jump-Ea thus signifies a high jump-frequency for the 16e-48h jump. As a general note, such high jump-frequencies between positions may occur if the start position is not a site (i.e. not a minimum on the energy landscape). For the present study this would suggest that the 16e position is not a site for the cases of 100% and 0% inversion of occupation between 4a and 4d sites.

with 100% S/Br-site inversion does not feature 48h-48h (1.9 Å) jump with activation energy < 0.3 eV. The inability to perform either a 48h–16e or a 48h–48h (1.9 Å) jump prevents macroscopic diffusion of lithium. Thus, in both cases, no percolating path exists with an activation energy below 0.3 eV.

In contrast to ordered configurations, the disordered arrangement of S/Br creates a variation of jump local environments, enabling low-energy pathways. To investigate the effect of S/Br-site disorder on lithium percolation network, we generated Li_6PS_5Br with 50% S/Br-site inversion which encounters every possible jump local environment with equal probability, as depicted by the width of the violin plot in Figure S26b. Li_6PS_5Br with 50% S/Br-site inversion exhibits local environments with jump activation energy < 0.3 eV for each jump type. Thus, similar to disordered-Li_9S_3N, in disordered Li_6PS_5Br with 50% S/Br-site inversion, percolating paths with jump activation energy < 0.3 eV may exist.

To further investigate the role of S/Br local arrangements in the macroscopic diffusion of lithium, we employed a percolation model as was done in the main text for disordered-Li_9S_3N. We created structures of Li_6PS_5Br with 0% S/Br-site inversion and Li_6PS_5Br with 100% S/Br-site inversion, both without site disorder, as well as Li_6PS_5Br with 50% S/Br-site inversion, with bromine equally distributed across the 4a and 4d sites, in a 5x5x5 supercell. The results of our percolation analysis are demonstrated in Figure S27.

Figures S27a and b show that for Li_6PS_5Br with 0% and 100% S/Br-site inversion, respectively, no percolating path exists when the cutoff jump energy is set to 0.3 eV. Connections are formed only within the isolated cages with no possibility of long-range lithium diffusion. In contrast, in the case of Li_6PS_5Br with 50% S/Br-site inversion, a percolation network is observed with the same cutoff jump energy of 0.3 eV (Figure S27c). Approximately 70% of sites participate in percolation, while some sites remain disconnected from the percolation network and require higher energy to become accessible.

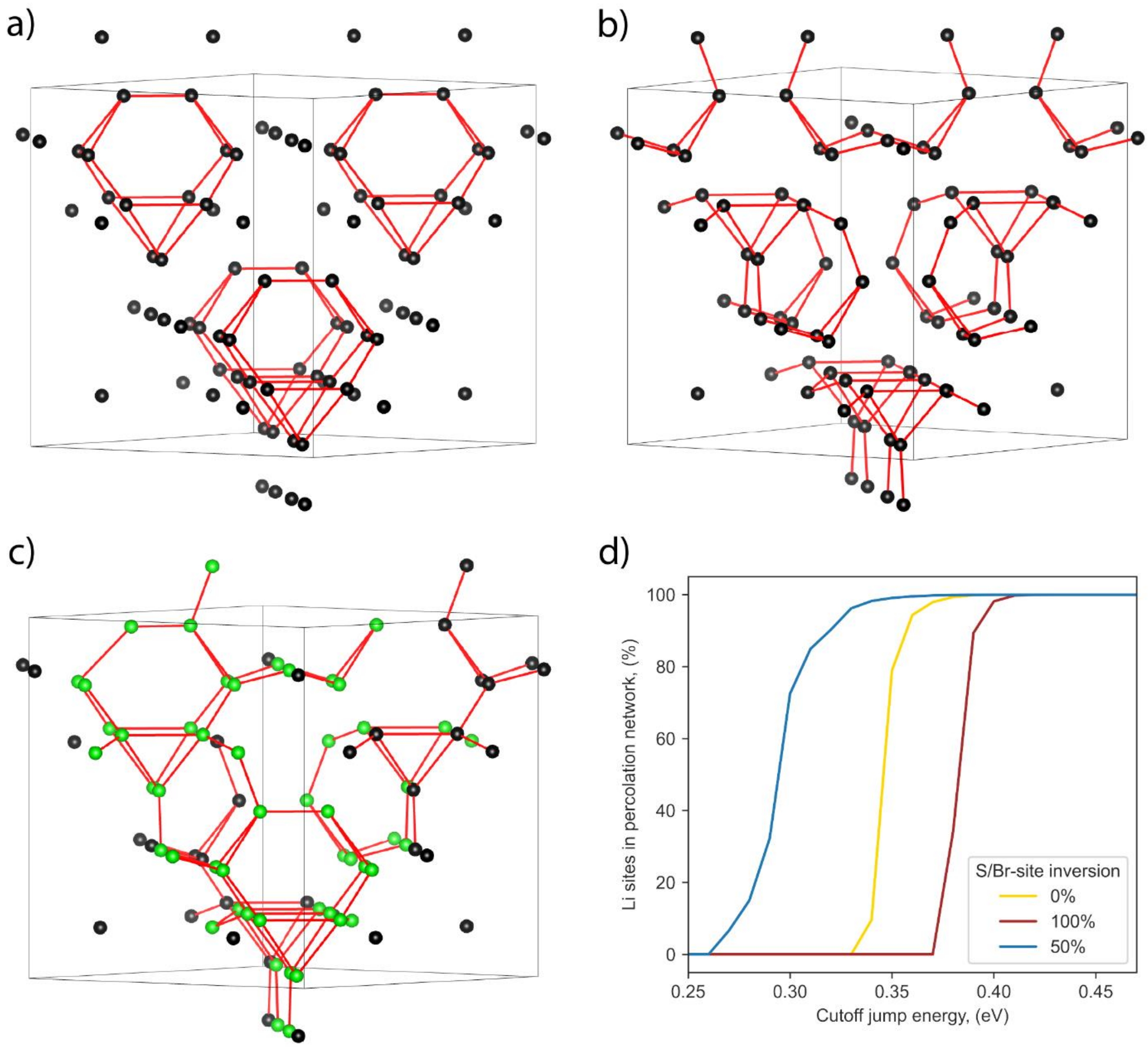

Figure S27. Analysis of percolation network in Li_6PS_5Br. Connected sites within the 0.3 eV cutoff jump energy (in the forward and backward direction) are indicated in red. The sites highlighted in green participate in percolation, while the sites highlighted in black are not connected to the percolation network. a) Every possible lithium jump in Li_6PS_5Br (0% S/Br-site inversion) with a cutoff jump energy of 0.3 eV. No percolation observed. b) Every possible lithium jump in Li_6PS_5Br (100% S/Br-site inversion) with a cutoff jump energy of 0.3 eV. No percolation observed. c) Every possible lithium jump in Li_6PS_5Br (50% S/Br-site inversion) with a cutoff jump energy of 0.3 eV. Around 70% of sites are connected to the percolation network. d) Energy-percolation diagram showing the fraction of Li sites connected to a percolating network for Li_6PS_5Br (0% S/Br-site inversion) in yellow, Li_6PS_5Br (100% S/Br-site inversion) in red, and Li_6PS_5Br (50% S/Br-site inversion) in blue (averaged over twenty 5x5x5 supercells with sulfur and bromine randomly distributed), as detailed in the Computational Details section.

Figure S27d shows the fraction of lithium sites connected to a percolating network as a function of cutoff energy value. For both ordered structures Li_6PS_5Br (0% S/Br-site inversion) and Li_6PS_5Br (100% S/Br-site inversion), percolation becomes possible only with cutoff energy values higher than the corresponding average activation energy of the rate-limiting jumps: 48h–16e at 0.36±0.04 eV and 48h–48h (1.9 Å) at 0.39±0.04 eV, respectively (Table S18). In contrast, for the Li_6PS_5Br (50% S/Br-site inversion) structure, percolation is possible with a cutoff energy of 0.27 eV, where all types of jumps (48h–48h, 1.9 Å; 48h–48h, 2.25 Å; 48h–16e; and 16e–48h) are available according to local environment-specific jump activation energies (Figure 26b, highlighted by the red dashed line).

The findings from the percolation model suggest that while both low-energy and high-energy local environments appear in the ordered variants, S^{2-}/Br^{-} disorder across both sublattices enables percolation through Li_6PS_5Br by striking a balance between the three jump-types needed for percolation. This disorder introduces a range of activation energies, thereby facilitating the formation of percolating pathways at lower energy thresholds. This investigation thus reveals what local environments enable fast Li-diffusion and how these local environments need to be connected to enable long-scale fast diffusivity. The lower onset for 50% site-inversion suggests that faster Li-diffusivity is possible compared to 0% site-inversion which correlates with the larger conductivity of Li_6PS_5Br phases with increasing site-inversion (i.e. increasing Br occupation on 4d sites) shown e.g. in ref.[24]. The percolation energy diagrams however suggest, that this trend is not monotonous, the percolation-energy diagrams predict that in the case of 100% sites inversion the ability of Li_6PS_5Br is similar to 0% site-inversion (this may however not be verified experimentally as samples with 100% site-inversion cannot be synthesized).

Table S18. The activation energies associated with different lithium ion jump types and environments within Li_6PS_5Br structures, showcasing variations across samples with different degrees of site disorder and ordering. These values are calculated from AIMD simulations conducted at 900 K.

**Jump Type**	**Jump Environment**			**0% S/Br-site inversion**	**12% S/Br-site inversion**		**38% S/Br-site inversion**				**100% S/Br-site inversion**	**Average**	**Standard Deviation**
	4a	4d	4a	(1)	(1)	(2)	(1)	(2)	(3)	(4)	(1)		
**48h–48h** (1.9 Å)	Br	S	Br	0.169	0.176	0.172	0.167	0.176	0.171	0.176		0.172	0.003
	Br	S	S		0.214	0.219	0.203	0.215	0.218	0.216		0.214	0.005
	S	S	Br		0.238	0.241	0.230	0.237	0.240	0.239		0.237	0.004
	Br	Br	Br		0.282	0.244	0.245	0.261	0.247	0.263		0.257	0.013
	S	S	S				0.278	0.279	0.252	0.284		0.274	0.012
	Br	Br	S		0.284		0.311	0.293	0.282	0.274		0.289	0.013
	S	Br	Br		0.334		0.347	0.336	0.321	0.321		0.332	0.010
	S	Br	S				0.383	0.411	0.367	0.390	0.413	0.393	0.018
**48h–48h** (2.25 Å)	Br	Br			0.199	0.217	0.202	0.206	0.216	0.209		0.208	0.007
	S	Br			0.211		0.205	0.213	0.221	0.219	0.191	0.210	0.010
	Br	S		0.253	0.257	0.253	0.238	0.244	0.243	0.245		0.247	0.007
	S	S			0.251	0.247	0.251	0.252	0.241	0.245		0.248	0.004
**48h–16e**	S	Br			0.181		0.181	0.178	0.191	0.176	0.167	0.179	0.007
	Br	Br			0.278	0.263	0.242	0.268	0.245	0.256		0.259	0.013
	S	S			0.300	0.302	0.257	0.275	0.277	0.289		0.283	0.016
	Br	S		0.410	0.400	0.383	0.342	0.346	0.342	0.360		0.369	0.026
**16e–48h**	S	Br			0.041		0.048	0.042	0.048	0.027	0.020	0.038	0.010
	Br	Br			0.059	0.042	0.022	0.047	0.032	0.030		0.039	0.012
	Br	S		-0.014	0.033	0.030	0.058	0.056	0.084	0.059		0.044	0.029
	S	S			0.065	0.005	0.074	0.092	0.068	0.083		0.064	0.028

Computational details for the Li_6PS_5Br example

Density functional theory (DFT) calculations based on the Perdew–Burke–Ernzerhof functional for solid-state systems (PBEsol)[25] within the Vienna Ab initio Software Package (VASP 6.3.2)[26] were utilized. Projector augmented wave (PAW)[27] potentials were used with cores of [He] for Li, [Ne] for P and S, and [Ar] for Br. Structure optimizations were conducted with an energy cutoff of 340 eV in 2x1x1 argyrodite supercells.

Ab initio molecular dynamics (AIMD) simulations in the canonical (NVT) ensemble using the Nosé-Hoover thermostat[28,29] were performed at 900 K. The energy cutoff was reduced to 300 eV, and gamma-only k-point mesh was used. The selected time step was 2 fs for a total computational time of 150 ps. Site-sensitive properties such as site-specific jump frequencies, and energy barriers were obtained using the analysis tools developed in our group. [14,30] To analyze the individual jumps, we defined two types of Li positions in the crystal lattice, namely, Wyckoff 48h and Wyckoff 16e (ref. [31]), counting the times Li resides in these positions throughout the simulation as well as the number of hops between these positions.

Four argyrodite structures Li_6PS_5Br were generated labelled by percentage of site inversion. Among these, Li_6PS_5Br (12% S/Br-site inversion) and Li_6PS_5Br (38% S/Br-site inversion) structures reflect the sublattice disorder, corresponding to experimentally determined site occupancies of sulfur and bromine[24]. These specific structures were selected to validate computational results by published experimental data. Since multiple orderings of S and Br in the sublattices can result in the same site occupancy, several unique configurations with random distribution of sulfur and bromine across 4a and 4d were optimized for both structures. Additionally, two configurations of Li_6PS_5Br exhibiting perfect order in the anionic sublattice, where bromine fully occupies 4a (0% S/Br-site inversion) or 4d (100% S/Br-site inversion) sites, were studied.

To analyze Li-ion jump activation energy specific to the local environments of sulfur and bromine, we examined AIMD simulations at 900 K for eight selected supercells: Li_6PS_5Br (0% S/Br-site inversion), two configurations of Li_6PS_5Br (12% S/Br-site inversion), four configurations of Li_6PS_5Br (38% S/Br-site inversion), and Li_6PS_5Br (100% S/Br-site inversion). These configurations had different S and Br distributions on the 4a and 4d sublattices, encompassing every possible local environment for each type of jump. Local environment-specific activation energies for all eight configurations are presented in Table S18.

For percolation analysis, we generated structures of Li_6PS_5Br (0% S/Br-site inversion), Li_6PS_5Br (100% S/Br-site inversion), and Li_6PS_5Br (50% S/Br-site inversion) in a 5x5x5 supercell using the pymatgen library[32] (version 2023.11.12). For Li_6PS_5Br (50% S/Br-site inversion), twenty configurations with random arrangements of sulfur and bromine across the 4a and 4d sublattices were generated. For each configuration, the percolation model was applied with twenty iterations. In each iteration, an environment-specific activation energy was randomly selected within ±0.04 eV from the average values listed in Table S16. This random selection accounts for the uncertainty in the activation energy values. The average results across all iterations and configurations per structure are analysed.

The results described in this Supplementary note have been published separately in [33].

Supporting Note 10: Discussions on the Arrhenius prefactor of $Li_{2+x}S_{1-x}N_x$ phases

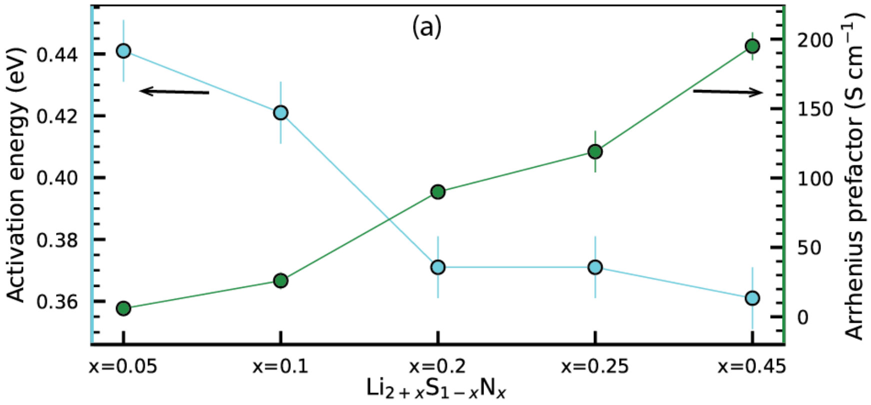

Figure S28 . Evolution of the Arrhenius prefactor and the activation energy obtained from EIS at variable temperatures for $Li_{2+x}S_{1-x}N_x$ phases.

With increasing Nitrogen content the experimental activation energy decreases in $Li_{2+x}S_{1-x}N_x$ phases but the Arrhenius prefactor increases. In the following we will discuss the possible origin of this observation

Based on the Nernst-Einstein relation and the definition of hop diffusivity, the Arrhenius prefactor (σ_0) can be written as shown in Equation S6[34].

$$\sigma_0 = \frac{\gamma a_0{}^2 v_0 (Ze)^2}{k} c_{ion} exp\left(\frac{-\Delta S_m}{k}\right) \quad (S6)$$

where γ is a geometrical factor, a_0 the average jump distance between sites, (Ze) the charge of the diffusing ion, v_0 the attempt frequency, k the Boltzmann constant, ΔS_m the entropy of migration and c_{ion} the charge-carrier concentration.

Our analysis reported in Supplementary Note 4 indicate that changes in the attempt are unlikely to explain the **18-fold** increase in σ_0 that we observe experimentally. Similarly a_0 which only changes slightly with nitrogen content.

Different physical expressions have been proposed for ΔS_m. [35–37] While ΔS_m remains ill-defined and no consensus is established on which relation is correct, the **existing** expressions for ΔS_m **unanimously** suggest that ΔS_m decreases with decreasing activation energy (ΔH_m), which amount to the enthalpy-entropy *compensation rule*, aka the *Meyer-Neldel rule* in the context of ion conduction.[37–39] The latter is typically demonstrated as a linear correlation between the measured (logarithm of the) conductivity prefactor ($\ln(\sigma_0) \propto \Delta S_m$) and activation energy ($Ea \propto \Delta H_m$) in a given structural framework; which is exactly the opposite of the trend we are observing in $Li_{2+x}S_{1-x}N_x$.

Thus, either the Meyer-Neldel rule does not hold in the $Li_{2+x}S_{1-x}N_x$ sample series, or there are differences in the mobile charge-carrier concentration that could explain the evolution of the prefactor with nitrogen content (x). The latter could be linked to the idea of active (percolating) versus inactive (non-percolating) lithium sites, explored in the manuscript.

Supporting Note 11: Characterization of Chemical and Electrochemical Stability of $Li_{2+x}S_{1-x}N_x$

To prove the stability of $Li_{2+x}S_{1-x}N_x$ against lithium metal, we added lithium metal to an excess of $Li_{2.3}S_{0.7}N_{0.3}$ and ground both substances together vigorously for 20 minutes. We use $Li_{2.3}S_{0.7}N_{0.3}$ as a representative of the antifluorite-like $Li_{2+x}S_{1-x}N_x$ solid solution. Figure S29a shows the X-ray diffraction pattern of $Li_{2.3}S_{0.7}N_{0.3}$ and Figure S29b shows the X-ray diffraction pattern of the $Li_{2.3}S_{0.7}N_{0.3}$ -lithium metal mixture. $Li_{2.3}S_{0.7}N_{0.3}$ was chosen as a representative of the antifluorite-like $Li_{2+x}S_{1-x}N_x$ ($0<x<0.55$) solid solution. No change in the diffraction pattern indicative of reductive decomposition of $Li_{2.3}S_{0.7}N_{0.3}$ may be observed in the $Li_{2.3}S_{0.7}N_{0.3}$ -lithium metal mixture. Diffraction peaks attributable to lithium metal are observed in the diffraction pattern of the $Li_{2.3}S_{0.7}N_{0.3}$ -lithium metal mixture indicating that that Li metal does not chemically react with $Li_{2.3}S_{0.7}N_{0.3}$.

To exclude the presence of amorphous decomposition products, we measured XPS (Figure S29c, d) of $Li_{2.3}S_{0.7}N_{0.3}$ and of the $Li_{2.3}S_{0.7}N_{0.3}$ -lithium metal mixture. No new peaks or peak-shifts to lower energies (as would be expected for a reductive decomposition of $Li_{2.3}S_{0.7}N_{0.3}$) can be observed in the S 2p and N 1s XPS spectra of the $Li_{2.3}S_{0.7}N_{0.3}$ -lithium metal mixture again indicating the stability of $Li_{2.3}S_{0.7}N_{0.3}$ against lithium metal. These experiments prove the stability of $Li_{2+x}S_{1-x}N_x$ phases against Li metal.

We repeated the same experiments with Li_6PS_5Cl instead of $Li_{2.3}S_{0.7}N_{0.3}$, to highlight the contrast between the intrinsic stability of fully reduced (irreducible) $Li_{2+x}S_{1-x}N_x$ phases and state of the art argyrodites solid electrolytes Figure S30**.** During grinding of Li_6PS_5Cl with lithium metal a vigorous reaction occurred, including observations of a flame (in the glovebox). The XRD of the Li_6PS_5Cl-lithium mixture clearly shows reduction products of Li_6PS_5Cl (LiCl, Li_2S) and the XPS of the Li_6PS_5Cl-lithium mixture shows clear reduction peaks.

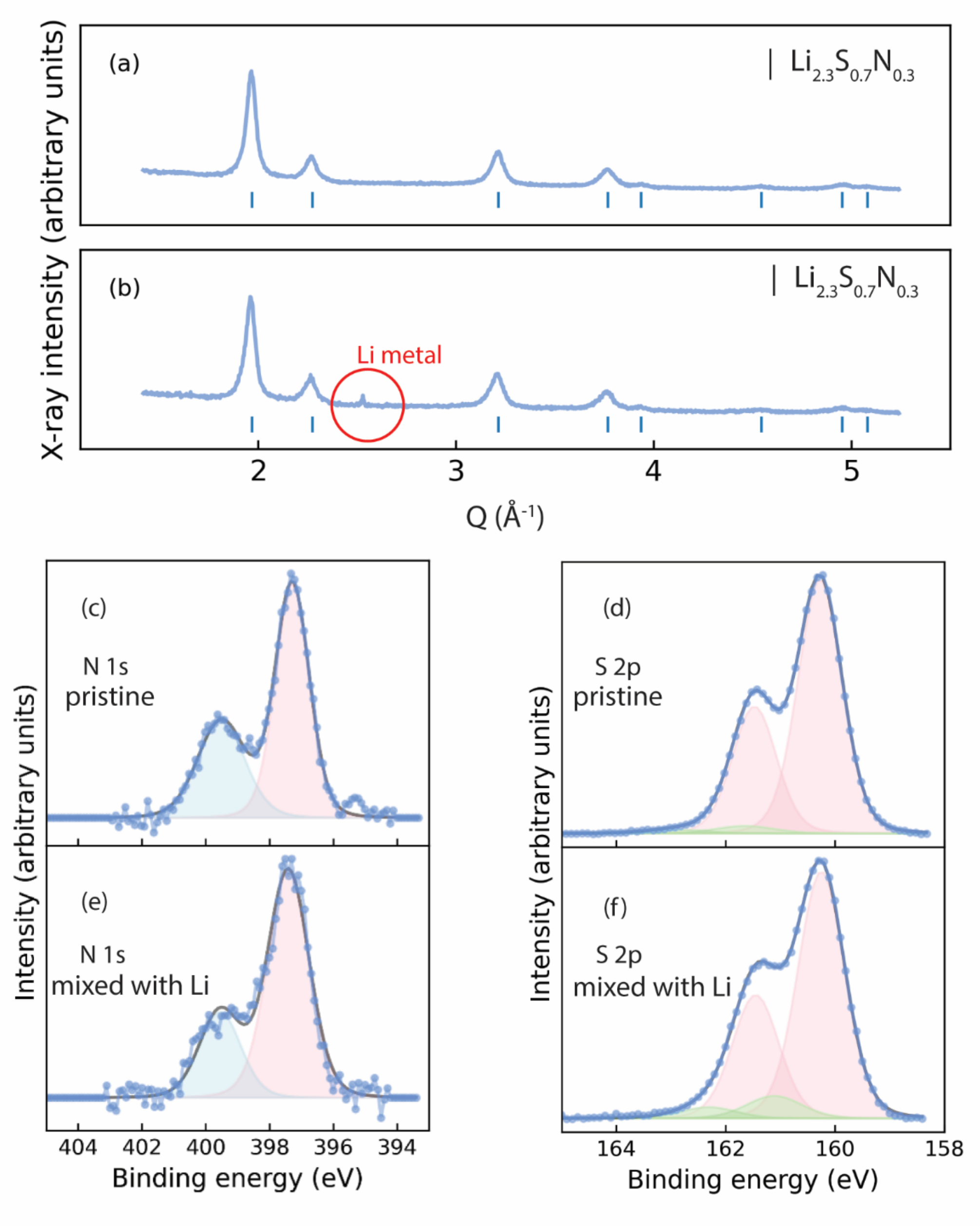

Figure S29. Investigation of chemical stability of the $Li_{2.3}S_{0.7}N_{0.3}$ phases against Li metal as a representative of the $Li_{2+x}S_{1-x}N_x$ phases. (a) X-ray diffraction pattern of $Li_{2.3}S_{0.7}N_{0.3}$ phases. (b) X-ray diffraction pattern of $Li_{2.3}S_{0.7}N_{0.3}$ vigorously mixed with Li metal. No decomposition product visible. Red circle indicates the strongest peak of the Li-metal diffraction pattern and thus indicates the presence of Li-metal. (c) N1s XPS of $Li_{2.3}S_{0.7}N_{0.3}$. (d) N1s XPS of $Li_{2.3}S_{0.7}N_{0.3}$ vigorously mixed with Li metal. *No* new peak or increase in relative peak intensities at lower energies which would be indicative of reductive decomposition. (e) S2p XPS of $Li_{2.3}S_{0.7}N_{0.3}$ (f) S2p XPS of $Li_{2.3}S_{0.7}N_{0.3}$ vigorously mixed with Li metal. *No* new peak or increase in relative peak intensities at lower energies which would be indicative of reductive decomposition.

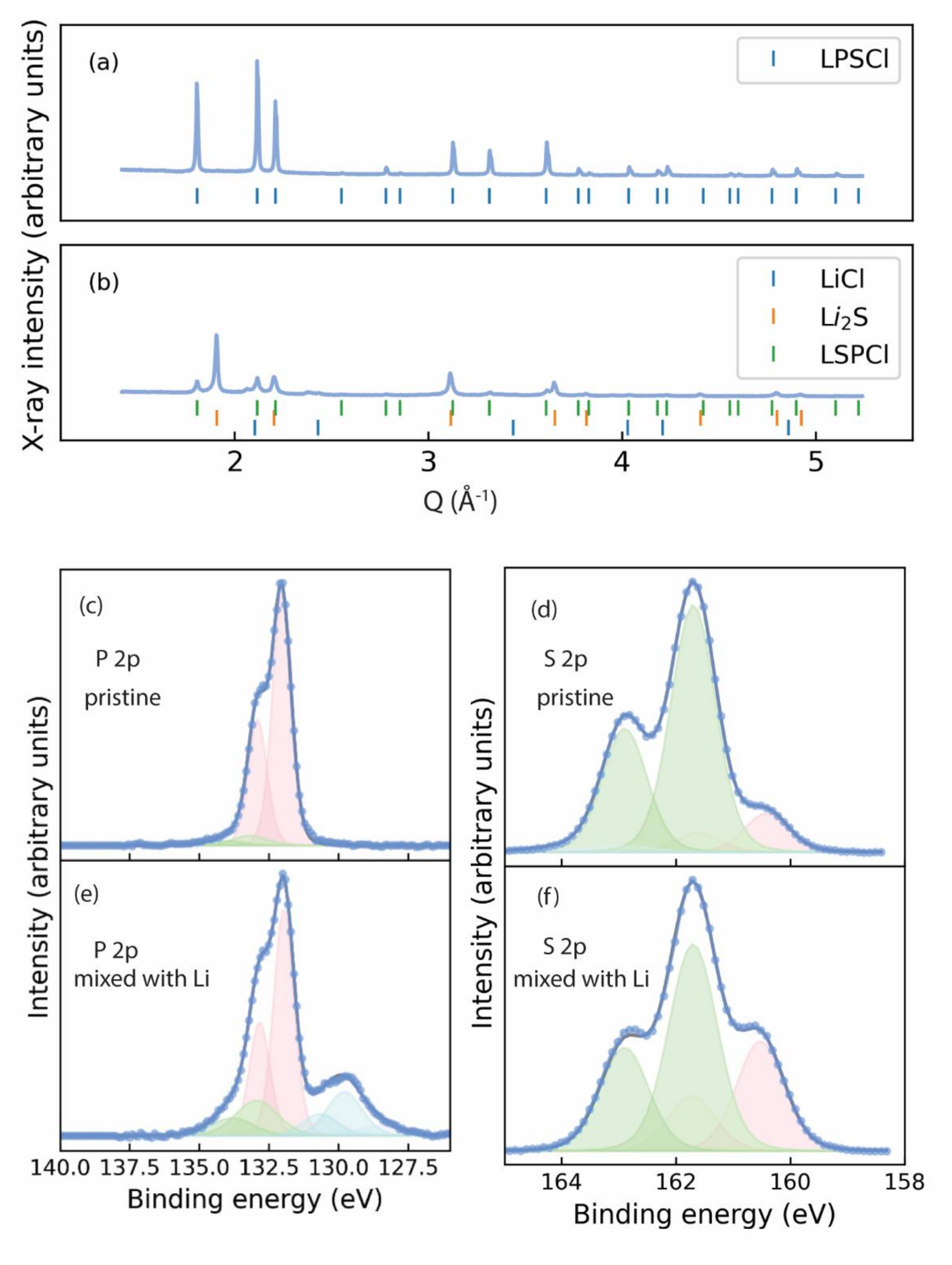

Figure S30. (a) Investigation of chemical stability of the LPSCl phases against Li metal to prove effectiveness of the approach to detect decomposition against Li metal. (a) X-ray diffraction pattern of LPSCl phases. (b) X-ray diffraction pattern of LPSCl vigorously mixed with Li metal. No decomposition product visible. Red signal indicates the strongest peak of the LI-metal diffraction pattern. (c) P2p XPS of LPSCl. (d) P2p XPS of LPSCl vigorously mixed with Li metal. Clear reductive peaks at low energies can be observed. (e) S2p XPS of LPSCl (f) S2p XPS of LPSCl vigorously mixed with Li metal. Clear reductive peaks at low energies can be observed.

So far we proved chemical stability of the $Li_{2+x}S_{1-x}N_x$ phases against metallic Li. Chemical stability against Li metal is equivalent to electrochemical stability at 0V (i.e. electrochemical stability against Li-metal) for the following reason based on ref [40]:

Chemical stability means that no reaction exists where

$$a * \Delta G_{f,LSN} + n * \ \mu_{Li,Li-metal} = \sum c_i \Delta G_{f,i} \qquad \Delta_r G < 0 \quad (S7)$$

In equation (S7) $\Delta G_{f,LSN}$ stands for the Gibbs free energy of formation of an arbitrary $Li_{2+x}S_{1-x}N_x$ (0<x<0.55) phase, $\mu_{Li,\ Li\text{-}metal}$ is the chemical potential of Li in Li metal, a, n and c_i are arbitrary stoichiometric factors and *i* is a possible reaction product between an arbitrary $Li_{2+x}S_{1-x}N_x$ (0<x<0.55) phase and Li metal.

Electrochemical stability means that no reaction exists between the material of interest and a lithium repository where lithium is at a chemical potential of

$$\mu_{Li,repository} = \ \mu_{Li,Li-metal} - \Phi\mathrm{e} \qquad (S8)$$

where ϕ is an arbitrary potential (vs Li/Li^+). In other words electrochemical stability at a potential ϕ (vs Li/Li^+) means that no reaction exists where

$$a * \Delta G_{f,LSN} + n * \ (\mu_{Li,Li-metal} - \Phi\mathrm{e}) = \sum c_i \Delta G_{f,i} \qquad \Delta_r G < 0 \quad (S9)$$

It can be seen that equation S7 and equation S9 are the same at a potential ϕ = 0 V vs Li/Li^+. It follows from this that electrochemical stability at 0 V vs Li is equivalent to chemical stability against Li-metal and *vice versa*.

Moreover we proved the stability of $Li_{2+x}S_{1-x}N_x$ (0<x<0.55) against Li metal by cycling Li|$Li_{2.25}S_{0.75}N_{0.25}$|Li symmetric cells showing no voltage increase that would suggest decomposition for 1000 h of cycling (Figure S31a). Additionally, we show that $Li_{2+x}S_{1-x}N_x$ (0<x<0.55) phases may be used to protect solid-electrolytes such as the Li_2ZrCl_6 electrolyte for example against Li-metal (Figure S31b). Figure S31b shows a rapid increase of the voltage in Li| Li_2ZrCl_6|Li cells indicative of solid-electrolyte decomposition against lithium. No such increase is seen when the Li_2ZrCl_6 is protected with $Li_{2.25}S_{0.75}N_{0.25}$.

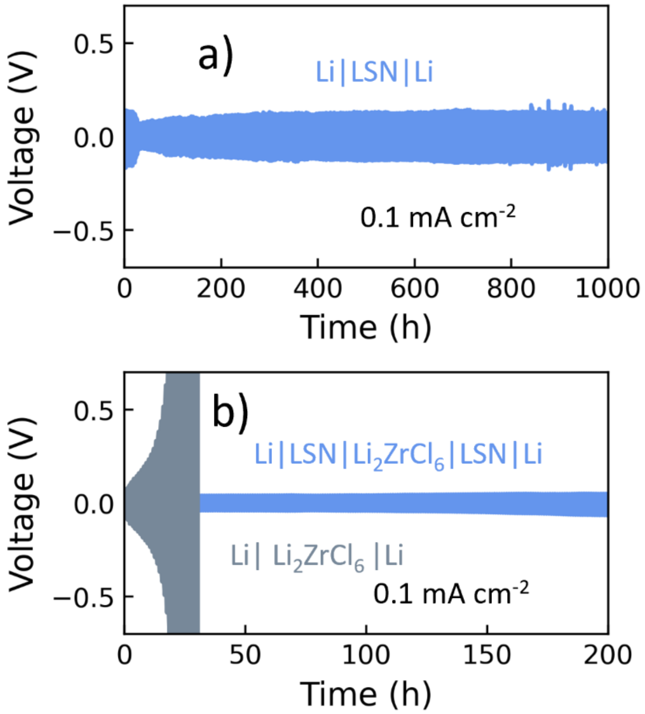

Figure S31. Cycling profiles of symmetric cells. (a) Li|$Li_{2.25}S_{0.75}N_{0.25}$|Li symmetric cell 0.01 mA cm^{-2} for 0.01 mA h cm^{-2}. (b) A protected cell Li|$Li_{2.25}S_{0.75}N_{0.25}$|$Li_2ZrCl_6$| $Li_{2.25}S_{0.75}N_{0.25}$|Li cell and an unprotected cell (grey trace) Li|Li_2ZrCl_6|Li cell 0.01 mA cm^{-2} for 0.01 mA h cm^{-2}. Protecting Li_2ZrCl_6 against Li with $Li_{2.25}S_{0.75}N_{0.25}$ highly improves the cycling performance and eliminates the catastrophic voltage increase caused by the decomposition of Li_2ZrCl_6. LSN in the legend stands for $Li_{2.25}S_{0.75}N_{0.25}$.

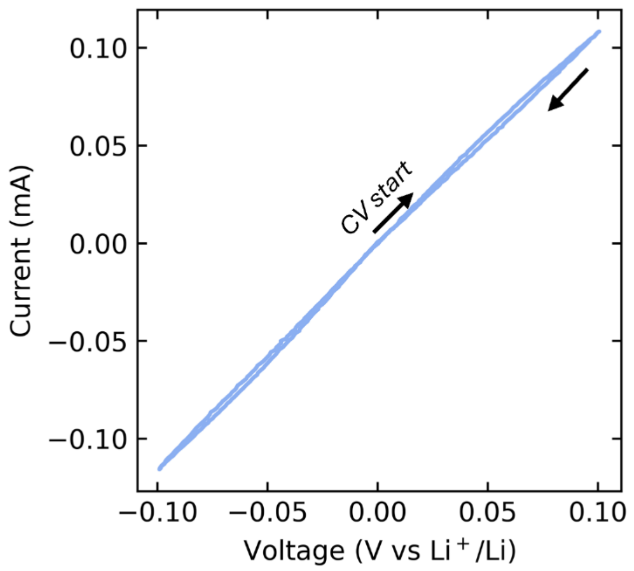

Figure S32. Cyclic voltammogram (CV) of a Li/$Li_{2.45}S_{0.55}N_{0.45}$/Li symmetric cell. Cycling was initiated from open circuit voltage (0 V). Scanning was first started in the oxidative direction (i.e. from 0 V to 0.1 V as indicated by arrows and the words "CV start"). Scanning rate was 0.1 mV/s. No reductive current above 0 V (vs Li) was observed further supporting that the antifluorite-like $Li_{2+x}S_{1-x}N_x$ electrolytes are irreducible and thus stable against reduction at low potentials.

Methodology for Supplementary Note 11: ***XPS:*** Measurements were performed with a ThermoFisher K-Alpha spectrometer to investigate the chemical state of the elements present. The spectrometer is equipped with a focused monochromatic Al kα source (1486.6eV) anode operating at 36W (12 kV, 3mA), a flood gun operating at 1V, 100µA, and the base pressure in the analysis chamber is approximately 2×10^{-9} mbar. The spot-size is approximately 800 x 400 μm^2. The pass energy of the analyzer was set to 50 eV. In the analysis, the binding energy was corrected for the charge shift by taking the done relative to the primary C1s hydrocarbon peak at *BE* = 284.8 eV as a reference. The peaks were fitted using 70% Gaussian and 30% Lorentzian line shapes (weighted least-squares fitting method) and nonlinear Shirley-type background using the ThermoFisher Avantage software. ***X-ray Diffraction:*** Powder diffraction patterns were collected using Cu Kα X-rays (1.54 Å) on a PANalytical X'Pert Pro X-ray diffractometer. The air sensitive $Li_{2+x}S_{1-x}N_x$ probes were loaded into air-tight holders in an Ar-filled glovebox prior to the measurements***. Assembly of symmetric cells:*** Symmetric cells were assembled custom-made slid state cells by pressing 60 mg of Li_2ZrCl_6 at 1 ton (on 10 mm diameter). Subsequently 20 mg LSN were added on each side and pressed at 2.7 tons. Subsequently 100 µm Li disks were placed on each side. The custom made solid state cells were then tightened by hand which applies ~5 MPa of pressure during cycling.***Cyclic Voltammetry***: 10 mm $Li_{2.45}S_{0.55}N_{0.45}$ pellets were pressed at 2.5 tons. Subsequently, 8 mm Li discs were placed on each side of the pellet. The custom-made solid state cells were then closed and tightened by hand. Scanning was initiated from open-circuit voltage (0 V) and the direction of scanning was 0 V to 0.1 V, 0.1V to 0 V, 0 V to -0.1 V, -0.1 V to 0 V. Scanning rate was 0.1mV/s

Author Contributions

The study was conceptualized by V.L. Simulation data were acquired by V.L ($Li_{2-x}S_{1-x}N_x$), A.V. and A.L. ($Li_{6-x}PS_{5-x}Br_{1+x}$). Experimental data were acquired by V.L, M.T., J.d.L., W.Z. and Z.C. (synthesis, x-ray diffraction, electrochemistry), J.C. (x-ray photoelectron spectroscopy), T.F. (neutron diffraction). Data analysis and interpretation were done by V.L., T.F , M.T. and M.W. Writing and editing of the draft were done by V.L., T.F., M.W., A.V. and S.G. The funding for this study was acquired by M.W and T.F. The work was supervised by T.F. and M.W. All authors have approved the submitted version of the manuscript.

References

1. Marx, R., Lissner, F. & Schleid, T. Li_9NS_3: Das erste Nitridsulfid der Alkalimetalle in einer Li_2O-Typ-Variante. *Zeitschrift für Anorg. und Allg. Chemie* **632**, 2151 (2006).

2. Bérar, J. F. & Lelann, P. E.s.d.'s and estimated probable error obtained in Rietveld refinements with local correlations. *J. Appl. Crystallogr.* **24**, 1–5 (1991).

3. Shannon, R. D. Revised effective ionic radii and systematic studies of interatomic distances in halides and chalcogenides. *Acta Crystallogr. Sect. A Cryst. physics, diffraction, Theor. Gen. Crystallogr.* **32**, 751–767 (1976).

4. Dong, Y. & DiSalvo, F. J. Reinvestigation of trilithium phosphide, Li_3P. *Acta Crystallogr. Sect. E Struct. Reports Online* **63**, i97–i98 (2007).

5. Szczuka, C. *et al.* Forced Disorder in the Solid Solution Li_3P–Li_2S: A New Class of Fully Reduced Solid Electrolytes for Lithium Metal Anodes. *J. Am. Chem. Soc.* **144**, 16350–16365 (2022).

6. Morgan, B. J. Mechanistic Origin of Superionic Lithium Diffusion in Anion-Disordered Li_6PS_5X Argyrodites. *Chem. Mater.* **33**, 2004–2018 (2021).

7. He, X., Zhu, Y. & Mo, Y. Origin of fast ion diffusion in super-ionic conductors. *Nat. Commun.* **8**, 1–7 (2017).

8. Catlow, C. R. A. Static lattice simulation of structure and transport in superionic conductors. *Solid State Ionics* **8**, 89–107 (1983).

9. Van der Ven, A., Ceder, G., Asta, M. & Tepesch, P. D. First-principles theory of ionic diffusion with nondilute carriers. *Phys. Rev. B* **64**, 184307 (2001).

10. Almond, D. P., Duncan, G. K. & West, A. R. The determination of hopping rates and carrier concentrations in ionic conductors by a new analysis of ac conductivity. *Solid State Ionics* **8**, 159–164 (1983).

11. Wang, Z. *et al.* Kinetic Monte Carlo Simulations of Sodium Ion Transport in NaSICON Electrodes. *ACS Mater. Lett.* **5**, 2499–2507 (2023).

12. Deng, Z. *et al.* Fundamental investigations on the sodium-ion transport properties of mixed polyanion solid-state battery electrolytes. *Nat. Commun.* **13**, 4470 (2022).

13. Li, W. *et al.* Lithium-compatible and air-stable vacancy-rich Li9N2Cl3 for high–areal capacity, long-

cycling all–solid-state lithium metal batteries. *Sci. Adv.* **9**, eadh4626 (2023).

14. De Klerk, N. J. J., Van Der Maas, E. & Wagemaker, M. Analysis of Diffusion in Solid-State Electrolytes through MD Simulations, Improvement of the Li-Ion Conductivity in β-Li3PS4 as an Example. *ACS Appl. Energy Mater.* **1**, 3230–3242 (2018).

15. Li, X. *et al.* Hopping Rate and Migration Entropy as the Origin of Superionic Conduction within Solid-State Electrolytes. *J. Am. Chem. Soc.* **145**, 11701–11709 (2023).

16. Kraft, M. A. *et al.* Influence of lattice polarizability on the ionic conductivity in the lithium superionic argyrodites Li_6PS_5X (X= Cl, Br, I). *J. Am. Chem. Soc.* **139**, 10909–10918 (2017).

17. Rice, M. J. & Roth, W. L. Ionic transport in super ionic conductors: a theoretical model. *J. Solid State Chem.* **4**, 294–310 (1972).

18. Gautam, A. *et al.* Engineering the Site-Disorder and Lithium Distribution in the Lithium Superionic Argyrodite Li_6PS_5Br. *Adv. Energy Mater.* **11**, (2021).

19. Zhao, E. *et al.* New insights into Li distribution in the superionic argyrodite Li_6PS_5Cl. *Chem. Commun.* **57**, 10787–10790 (2021).

20. Zhou, L., Minafra, N., Zeier, W. G. & Nazar, L. F. Innovative approaches to Li-argyrodite solid electrolytes for all-solid-state lithium batteries. *Acc. Chem. Res.* **54**, 2717–2728 (2021).

21. De Klerk, N. J. J., Rosłoń, I. & Wagemaker, M. Diffusion Mechanism of Li Argyrodite Solid Electrolytes for Li-Ion Batteries and Prediction of Optimized Halogen Doping: The Effect of Li Vacancies, Halogens, and Halogen Disorder. *Chem. Mater.* **28**, 7955–7963 (2016).

22. Zhou, L., Zhang, Q. & Nazar, L. F. Li-rich and halide-deficient argyrodite fast ion conductors. *Chem. Mater.* **34**, 9634–9643 (2022).

23. Hogrefe, K. *et al.* Opening diffusion pathways through site disorder: the interplay of local structure and ion dynamics in the solid electrolyte $Li_{6+x}P_{1-x}Ge_xS_5I$ as probed by neutron diffraction and NMR. *J. Am. Chem. Soc.* **144**, 1795–1812 (2022).

24. Gautam, A., Al-Kutubi, H., Famprikis, T., Ganapathy, S. & Wagemaker, M. Exploring the Relationship Between Halide Substitution, Structural Disorder, and Lithium Distribution in Lithium Argyrodites ($Li_{6-x}PS_{5-x}Br_{1+x}$). *Chem. Mater.* **35**, 8081–8091 (2023).

25. Perdew, J. P., Burke, K. & Ernzerhof, M. Generalized gradient approximation made simple. *Phys. Rev. Lett.* **77**, 3865 (1996).

26. Kresse, G. & Furthmüller, J. Efficiency of ab-initio total energy calculations for metals and semiconductors using a plane-wave basis set. *Comput. Mater. Sci.* **6**, 15–50 (1996).

27. Blöchl, P. E. Projector augmented-wave method. *Phys. Rev. B* **50**, 17953 (1994).

28. Nosé, S. A unified formulation of the constant temperature molecular dynamics methods. *J. Chem. Phys.* **81**, 511–519 (1984).

29. Hoover, W. G. Canonical dynamics: Equilibrium phase-space distributions. *Phys. Rev. A* **31**, 1695 (1985).

30. Azizi, V., Smeets, S., Lavrinenko, A. K., Ciarella, S. & Famprikis, T. GEMDAT. at https://doi.org/10.5281/zenodo.12748848 (2024).

31. Kong, S. *et al.* Lithium argyrodites with phosphorus and arsenic: order and disorder of lithium atoms, crystal chemistry, and phase transitions. *Chem. Eur. J.* **16**, 2198–2206 (2010).

32. Ong, S. P. *et al.* Python Materials Genomics (pymatgen): A robust, open-source python library for materials analysis. *Comput. Mater. Sci.* **68**, 314–319 (2013).

33. Lavrinenko, A. K. *et al.* Optimizing Ionic Transport in Argyrodites: A Unified View on the Role of Sulfur/Halide Distribution and Local Environments. *J. Mater. Chem. A* 26596–26611 (2024) doi:10.1039/d4ta04628e.

34. Tilley, R. J. D. *Defects in Solids*. (John Wiley & Sons, 2008).

35. Wert, C. & Zener, C. Interstitial atomic diffusion coefficients. *Phys. Rev.* **76**, 1169–1175 (1949).

36. Dienes, G. J. Frequency factor and activation energy for the volume diffusion of metals. *J. Appl. Phys.* **21**, 1189–1192 (1950).

37. Almond, D. P. & West, A. R. The activation entropy for transport in ionic conductors. *Solid State Ionics* **23**, 27–35 (1987).

38. Gao, Y., Li, N., Wu, Y., Yang, W. & Bo, S. H. Rethinking the Design of Ionic Conductors Using Meyer–Neldel–Conductivity Plot. *Adv. Energy Mater.* **11**, 1–9 (2021).

39. Du, P., Zhu, H., Braun, A., Yelon, A. & Chen, Q. Entropy and Isokinetic Temperature in Fast Ion Transport. *Adv. Sci.* **11**, 1–8 (2024).

40. Zhu, Y., He, X. & Mo, Y. First principles study on electrochemical and chemical stability of solid electrolyte-electrode interfaces in all-solid-state Li-ion batteries. *J. Mater. Chem. A* **4**, 3253–3266 (2016).
